# Near‐Infrared Triggered Anion Transport Induces Cancer Cell Death

**DOI:** 10.1002/anie.202523734

**Published:** 2025-12-12

**Authors:** Manzoor Ahmad, Ríona M. Devereux, Angela J. Russell, Matthew J. Langton

**Affiliations:** ^1^ Chemistry Research Laboratory University of Oxford Mansfield Road Oxford OX1 3TA UK; ^2^ Department of Pharmacology University of Oxford Mansfield Road Oxford OX1 3QT UK

**Keywords:** Anion transport, Anions, Hydrogen bonding, Membranes, Stimuli‐responsive

## Abstract

Artificial transmembrane anion carriers have shown potential in biological research and medicine, such as chemotherapeutics to treat channelopathies and anticancer agents. Stimuli‐responsive systems, controlled by triggers such as light, pH, redox, enzymes, or membrane potential, offer the potential for targeted activation. Photoactivation of ion transport is particularly advantageous due to the possibility of achieving spatiotemporal control, remote addressability, and reduced cytotoxicity. However, poor tissue penetration and undesired cytotoxicity are significant drawbacks to many photo‐activated ionophores reported to date, which are mostly triggered by UV or violet light. Here, we report BODIPY‐caged photo‐responsive anionophores activated with NIR light, which utilize dynamic hydrogen bonding interactions of a 4‐hydroxyisophthalamide motif. Caging of the hydroxyl group of the anionophore with BODIPY‐photocages locks the amide proton through six‐membered intramolecular hydrogen bonding, rendering it unavailable for anion recognition and transport. Decaging with 730 nm NIR irradiation reverses the hydrogen bonding pattern to switch on binding, with efficient off‐on activation profiles observed in anion transport experiments in vesicles. Analogous experiments in cancer cells revealed turn‐on transmembrane chloride transport and a dramatic, dose‐dependent decrease in cell viability following NIR decaging of the anionophore, demonstrating the potential for NIR‐triggered ionophores as an alternative to existing photodynamic therapies for cancer.

## Graphical abstract

Photo‐responsive transmembrane anion transporters that are activated with NIR light are reported. Experiments in cancer cells revealed that NIR activation of the ionophore triggered near‐IR light‐dependent chloride influx and triggered cell death.

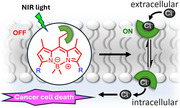

## Introduction

Ion transport across lipid bilayer membranes is essential for life, and is mediated by a range of protein channels, pumps, and carriers.^[^ ^1^, ^2^, ^3^, ^4^ ^]^ Their behavior is, in turn, typically regulated in response to external stimuli, including small molecules, light, or membrane potential.^[^ ^5^, ^6^ ^]^ Their role is to maintain and regulate ion concentrations necessary for cell homeostasis, and dysregulation of their function is implicated in numerous diseases.^[^ ^7^, ^8^ ^]^ For instance, dysfunction of Cl^−^ channels is associated with cystic fibrosis.^[^ ^9^, ^10^ ^]^ Artificial ion transport systems have emerged as an important class of compounds that may, in the future, be used to treat these conditions via “channel replacement therapy”.^[^ ^11^, ^12^, ^13^ ^]^

Synthetic chloride transporters have also been shown to induce chloride‐mediated cancer cell death.^[^ ^14^, ^15^, ^16^ ^]^ Such systems may offer an alternative to existing therapies by operating via disruption of cellular ion gradients, a mechanism that is independent of drug interactions with proteins that may be readily mutated, potentially minimizing the development of drug resistance.^[^ ^17^ ^]^ For example, Sessler et. al. have shown that prodigiosin‐based transporters show effective anticancer capabilities and deacidify acidic organelles through H^+^/Cl^−^ co‐transport to induce cell death in A549 human lung cancer cells.^[^ ^18^ ^]^ Similarly, perenosins,^[^ ^19^ ^]^ and thiophene‐based anionophores^[^ ^20^ ^]^ developed by Gale, as well as benzimidazole‐based anionophores developed by Chen,^[^ ^21^ ^]^ Manna,^[^ ^22^ ^]^ and Talukdar^[^ ^23^ ^]^ exhibit efficient anticancer activities in a range of cancer cells, again through H^+^/Cl^−^‐mediated deacidification in cell organelles.

Anionophores that function primarily as Cl^−^ antiporters also exhibit anticancer activity. For example, calix‐pyrrole and squaramide‐based transporters developed by Shin, Gale, Sessler, and co‐workers induce caspase‐dependent apoptosis in various cancer cells by increasing intracellular chloride and sodium ion concentrations.^[^ ^24^, ^25^ ^]^ Ultimately, the transporter‐mediated decrease in lysosome chloride concentrations and increase in pH block the autophagy function and induce apoptosis.^[^ ^26^ ^]^ Similarly, a range of TREN‐based tris‐(thio)ureas,^[^ ^27^ ^]^ 1,2‐diphenylethylenediamine‐bis‐thioureas,^[^ ^28^ ^]^ and bis(sulfonamide)‐based chloride anionophores^[^ ^29^ ^]^ have been shown to exhibit anticancer activities by perturbing cellular chloride ion concentrations.

Spatio‐temporal targeting or activation of anticancer agents to tumors is a key consideration for minimizing off‐target effects.^[^ ^30^ ^]^ Photodynamic therapy (PDT) is one such approach that uses light to activate a photosensitizer and destroy abnormal cells. Typically, near‐infrared (NIR) light within the “therapeutic window” is used for enhanced tissue penetration and is used to excite a photosensitizer to generate reactive oxygen species (ROS).^[^ ^31^ ^]^This approach can, however, be impeded by hypoxia, which frequently occurs in tumor microenvironments.^[^ ^32^ ^]^ Replacing photosensitizers with photo‐responsive anionophores whose activity is independent of tumor oxygen concentrations may provide an alternative approach to existing PDT methods. To date, a wide variety of photo‐responsive ion transport systems have been developed. These include reversibly gated systems that employ molecular photoswitches (including azobenzene, acylhydrazone, phenylhydrazone, stiff‐stilbene, and spiropyran)^[^ ^33^, ^34^, ^35^, ^36^, ^37^, ^38^, ^39^, ^40^ ^]^ or irreversibly gated systems based on photocages.^[^ ^41^, ^42^, ^43^ ^]^ Achieving a high level of control over transport activity using photoswitches is often hampered by background transport activity in their “off” state due to incomplete photo‐switching or fast thermal relaxation of one of the isomers. In contrast, irreversibly controlled transporters that are triggered by photo‐decaging reactions enable very effective off‐on control over transport.^[^ ^44^, ^45^ ^]^ Such systems have been utilized for anticancer applications in which light was used to trigger chloride‐mediated apoptosis in cancer cells. The reported systems, however, are triggered either by UV light or in the violet region, due to the use of *ortho*‐nitrobenzyl (ONB) photocages, ultimately limiting further application due to poor tissue penetration.^[^ ^46^, ^47^, ^48^, ^49^ ^]^

Here, we report—to the best of our knowledge— the first example of an ionophore activated using NIR light. We use an extended π‐conjugated BODIPY‐caged photo‐responsive chloride transporter that is activated by decaging with 730 nm light. This triggers a rearrangement of the hydrogen bonding framework in the anionophore and switches on chloride binding and transport. Photo‐triggered chloride transport is demonstrated in model large unilamellar vesicle (LUV) experiments, as well as the ability to trigger chloride transport across cellular membranes and cancer cell death upon NIR irradiation.

## Results and Discussion

### Approach

We recently reported a 4,6‐dihydroxyisophthalamide‐based stimulus‐responsive ion transport system that utilized dynamic hydrogen bonding interactions to trigger off‐on anion transport activity in lipid bilayer vesicles. Alkylation of the hydroxy groups with various caging moieties responsive to light, redox, and enzymes locks the anion‐binding amide protons through intra‐molecular hydrogen bonding, inhibiting anion binding and hence anion transport. Decaging of both hydroxy groups using the respective stimulus reverses the hydrogen bonding pattern such that the amide NH protons are available for anion binding and transport. Moreover, we observed that caging of only one hydroxy moiety was sufficient to inhibit the anion binding and transport.^[^ ^44^ ^]^

**Figure 1:**
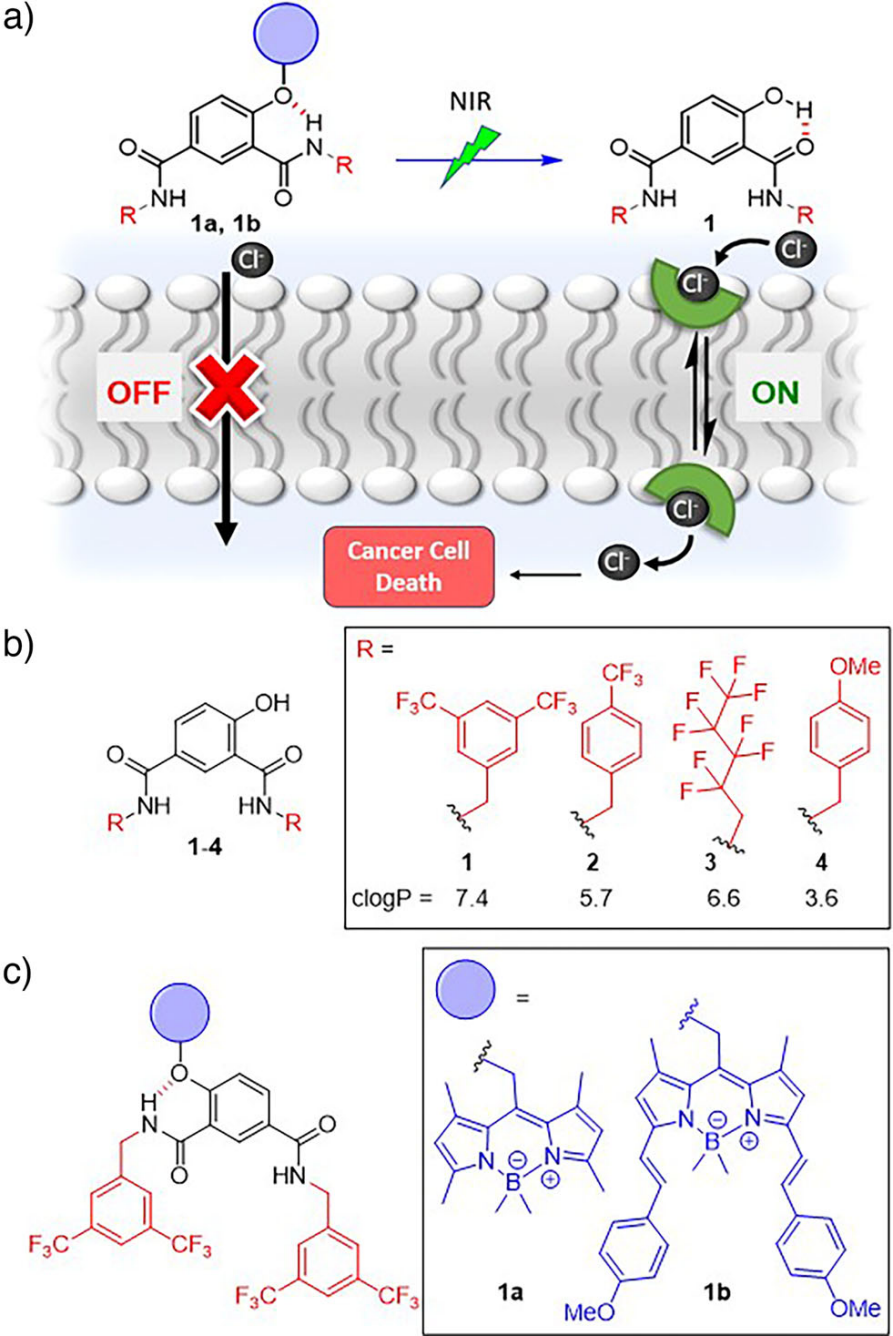
a) Schematic representation of NIR‐responsive chloride anion transport. b), c) Chemical structures of chloride transporters **1–4**, and caged pro‐transporters **1a** and **1b**.

To access a NIR activated anionophore, we identified BODIPY photo‐cages with highly extended π‐conjugation as suitable NIR responsive groups. However, we anticipated that incorporating two of these bulky lipophilic derivatives into a 4,6‐dihydroxyisophthalamide anion receptor would bring complications in chemical synthesis as well as poor membrane uptake and water solubility profiles. We therefore designed 4‐hydroxyisophthalamide anionophores requiring only one photo‐cage to deactivate transport (**1**–**4**). Similar to the previous dihydroxy‐derivatives, these function by locking the proton of the adjacent amide through six‐membered intramolecular hydrogen bonding to the ether O atom, suppressing anion recognition and transport. Decaging with light reverses the hydrogen bonding pattern to switch on anion binding and transport (Figure 1a). A library of four different transporters **1**–**4** containing variable aromatic moieties was designed and synthesized, varying the nature of the aromatic groups to tune lipophilicity (clogP values) and optimize the permeability and transport affinity of these anionophores (Figure 1b). Green light and NIR activated BODIPY cages were introduced to provide photocontrol (Figure 1c).

### Synthesis and Anion Binding Experiments of Anionophores **1–4**

**Scheme 1:**
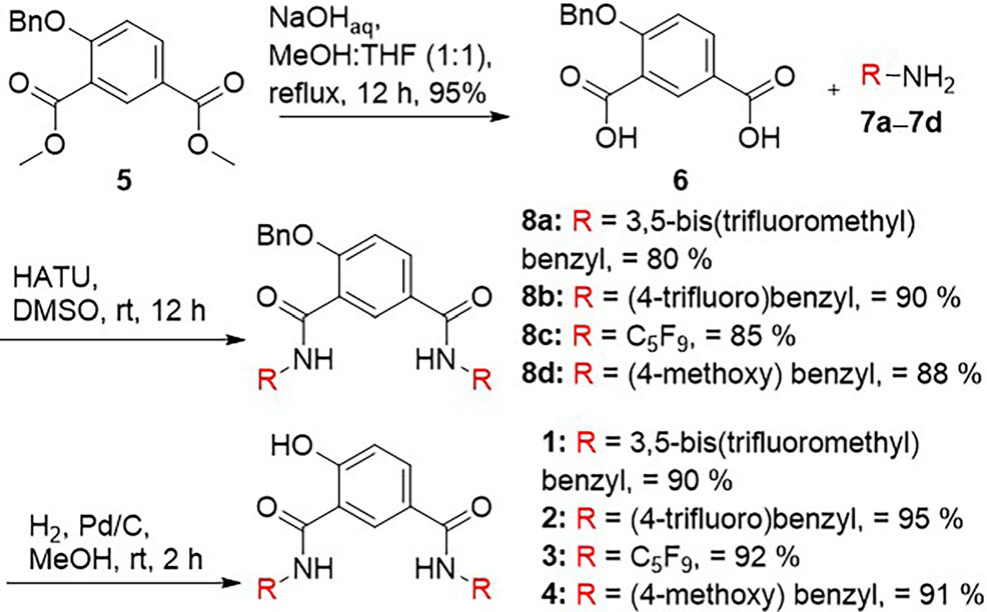
Chemical synthesis of active transporters **1–4**.

In order to synthesize chloride transporters **1**–**4**, benzylated‐ester **5** ^[^ ^50^ ^]^ was first hydrolyzed using aqueous sodium hydroxide to form **6**. This was coupled with amines **7a**‐**7d** using a HATU‐mediated coupling reaction to give amide derivatives **8a**‐**8d**. Finally, **8a**‐**8d** were debenzylated using hydrogen over Pd/C to furnish anionophores **1**–**4** in excellent yield (Scheme 1).

**Figure 2:**
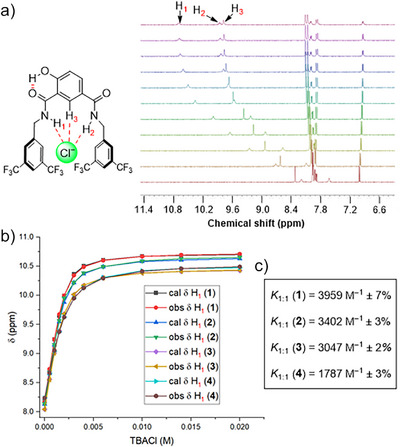
a) ^1^H NMR titration of **1** with tetrabutylammonium chloride (TBACl) in acetonitrile‒d_3_. b) Plot of chemical shift (*δ*) of H_1_ proton versus concentration of TBACl added, fitted to 1:1 binding isotherm for anionophores **1–4**. c) Binding constant values of transporters **1–4**.

With anionophores **1**–**4** in hand, their anion binding capabilities were investigated using ^1^H NMR titration experiments in acetonitrile‒*d*_3_. Addition of increasing equivalents of tetrabutylammonium chloride (TBACl) solution to receptors **1–4** led to a significant downfield chemical shift of the amide N‒H_1_, amide N‒H_2_, and CAr‒H_3_ protons (Figures 2a, S50, S52, S54, and S56), indicative of N‒H_1_···Cl^‒^, N‒H_2_···Cl^‒^, and CAr‒H_3_···Cl^‒^ hydrogen bonding interactions. Analysis of the generated binding isotherms using Bindfit^[^ ^51^ ^]^ determined 1:1 host:guest association constants (*K*_a_(1:1)/Cl^‒^) of 3959 M^‒1^ ± 7% for **1**, 3402 M^‒1^ ± 3% for **2**, 3047 M^‒1^ ± 2% for **3**, and 1787 M^‒1^ ± 3% for **4**, respectively (Figures 2b,c, S51, S53, S55, and S57), and hence the overall chloride binding affinity sequence was found to be **1**>**2**>**3**>**4**. Incorporation of amide linkages with benzyl substituents was expected to enhance the binding affinity due to aromatic CH─anion hydrogen bonding interactions, as we have previously observed for related systems.^[^ ^44^ ^]^ Here, we postulate that compound **1** with two trifluoromethyl groups exhibits enhanced anion affinity due to such intermolecular interactions. Evidence for **1** adopting the conformation pre‐organized for chloride binding as depicted in Figure 2a was obtained from ^1^H–^1^H NOESY NMR spectroscopy, in which through‐space correlations were observed between protons H_1_–H_3_ and H_2_–H_3_ (Figure S36).

### Anion Transport Experiments in Vesicles

**Figure 3:**
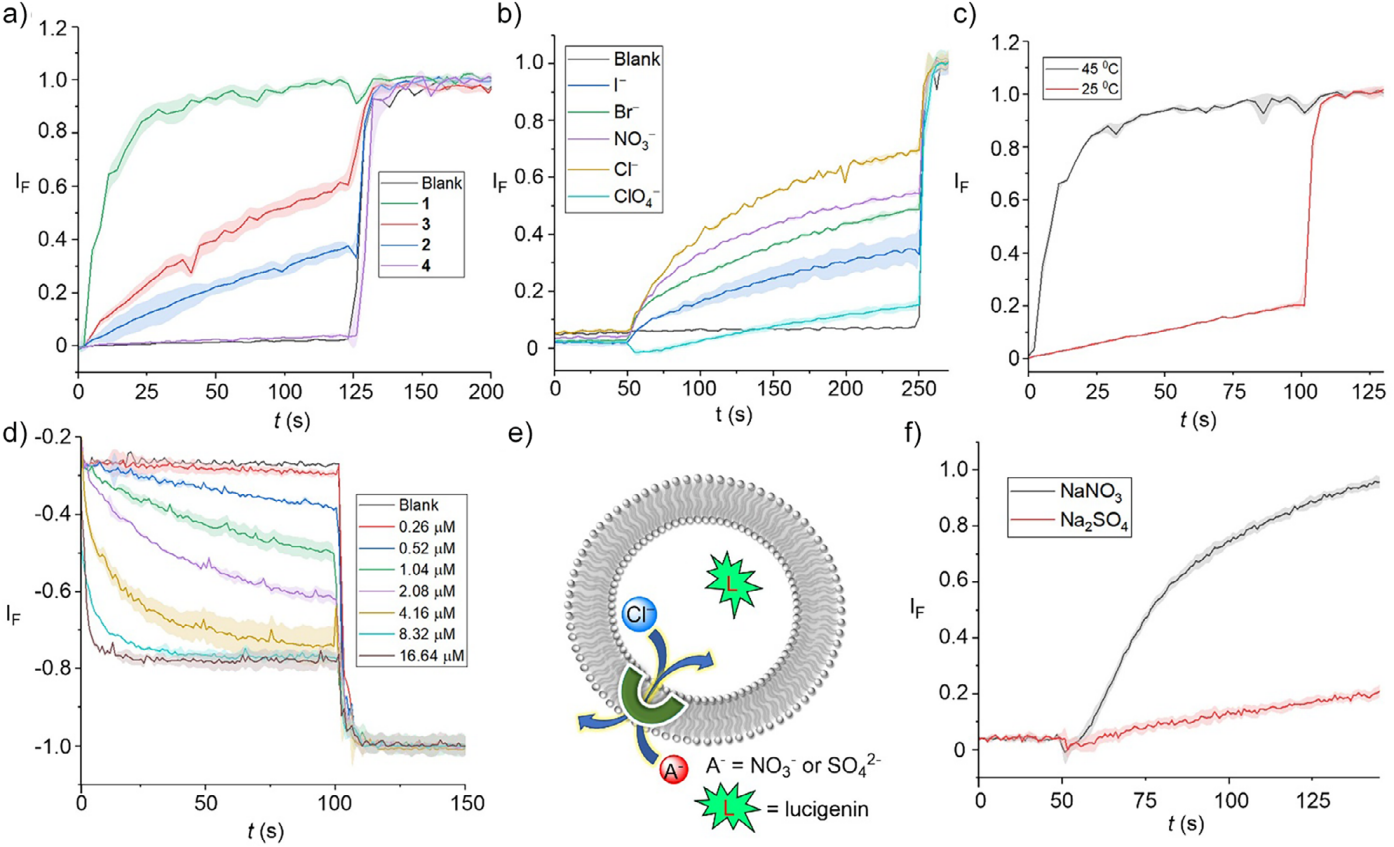
a) Activity comparison of **1‒4** (2.67 mol%) across POPC‒LUVs⊃HPTS. b) Anion selectivity of **1** (0.67 mol%) by using different extravesicular Na^+^/X^‒^ salts (X^‒^ = Cl^‒^, Br^‒^, I^‒^, NO_3_^‒^ and ClO_4_^‒^) across POPC–LUVs⊃HPTS. c) Ion transport activity of **1** (2.67 mol%) across DPPC‐based vesicles at 25 °C and 45 °C temperatures, respectively. d) Concentration‐dependent activity of compound **1** across POPC–LUVs⊃lucigenin. e) Schematic representation of lucigenin‐based chloride efflux using either extravesicular SO_4_^2−^ or NO_3_^−^ ions. F. Ion transport activity of **1** (7.63 mol%) in the presence of external SO_4_^2−^ and NO_3_^−^. Error bars represent standard deviations (*n* = 3).

Having determined the chloride binding efficacy of the transporters **1**–**4**, their anion transport capabilities were then evaluated. Initially, we performed pH‐dissipation anion transport experiments in large unilamellar vesicles (LUVs) containing the ratiometric pH‐sensitive fluorophore 8‐hydroxypyrene‐1,3,6‐trisulfonate (HPTS). LUVs containing HPTS (1 mM) were prepared, containing 100 mM NaCl and 10 mM HEPES buffer at pH 7. Subsequently, a pH gradient (pH_in_ = 7.0 and pH_out _= 7.8) was generated across the LUV lipid bilayer membrane upon addition of a pulse of NaOH (5 mM) to the extravesicular solution. After addition of compounds **1**–**4** in DMSO (<0.5 % v/v), the dissipation of the pH gradient through OH^−^/Cl^−^ antiport (or the functionally equivalent H^+^/Cl^−^ symport) was monitored by recording the change in fluorescence intensity *I*_rel_ (*λ*_em_ = 510 nm) with time, following excitation at *λ*_ex _= 405/460 nm. At the end of each experiment, excess detergent (Triton X‐100) was added to lyse the vesicles for calibration of the emission intensity. Significant ion transport was observed for all transporters with the activity sequence of **1**>**3**>**2**>**4**. The transport activity of all the transporters at 0.83 µM (2.67 mol% loading with respect to lipid) is shown in Figure 3a, and the corresponding dose‐response curves are given in the ESI (Figures S62, S64, S66, and S68). Hill analysis afforded the *EC*_50_ values of 0.16 µM ± 0.0105 (0.52 ± 0.01 mol%) for **1**, 1.37 µM ± 0.08 (4.41 ± 0.25 mol%) for **2**, and 0.87 µM ± 0.11 (2.81 ± 0.35 mol%) for **3**, respectively (Figures S63, S65, and S67). Hill analysis could not be performed for compound **4** because of precipitation at higher concentrations. Hill coefficients (*n*) were found to be ∼1 for compounds **1**–**3**, suggesting the involvement of a 1:1 receptor‐anion complex in the transmembrane anion transport process. The above activity sequences of **1**>**3**>**2**>**4** is presumably determined by a combination of both the respective lipophilicities and anion binding affinities of these transporters. Compound **1** with an optimum clogP value and chloride binding affinity exhibits the highest transport activity.

Chloride transport by the best‐performing transporter **1** was subsequently studied using lucigenin‐based LUV assays. LUVs entrapping 1.0 mM lucigenin–a fluorophore quenched by chloride–were prepared in 200 mM NaNO_3_ buffered to pH 6.5, and then a Cl^‒^/NO_3_^‒^ gradient was created by adding NaCl (33.3 mM) in the extravesicular buffer. The influx of chloride anions via a Cl^‒^/NO_3_^‒^ antiport mechanism mediated by **1** was evaluated by monitoring the rate of change in the fluorescence intensity (λ_ex_ = 455 nm and λ_em_ = 535 nm), and at the end of each experiment, excess detergent (Triton X‐100) was added to lyse the vesicles for calibration of the emission intensity. Significant quenching of the lucigenin was observed after the addition of transporter **1**. The dose‐dependent Cl^‒^ influx is shown in Figure 3d. Hill analysis furnished *EC*_50_ value of 1.25 ± 0.06 µM (1.14 ± 0.05 mol%) with a Hill coefficient value of ∼1, consistent with that obtained in the HPTS pH dissipation experiments (Figure S73).

Mechanistically, the disruption of the pH gradient across the membrane of the POPC LUVs containing HPTS can occur through antiport (OH^−^/X^−^ or H^+^/M^+^ exchange) or symport (H^+^/X^−^ or OH^−^/M^+^ co‐transport) mechanisms. However, using intracellular NaCl and an iso‐osmolar extravesicular M^+^/Cl^–^ salt (where M^+^ = Li^+^, Na^+^, K^+^, Rb^+^, Cs^+^), no change in ion transport activity was observed, which rules out the possibility of H^+^/M^+^ antiport and OH^−^/M^+^ symport mechanisms (Figure S69). Furthermore, changing the extravesicular Na^+^/X^–^ salt (X^‒^ = Cl^‒^, Br^‒^, I^‒^, NO_3_^‒^, ClO_4_^‒^) resulted in a significant modulation of the observed ion transport kinetics (Figure 3b), revealing the role of anions in an overall transport process, and hence suggest that the ion transport process occurs through either OH^−^/Cl^−^ antiport or H^+^/Cl^−^ symport modes. Similarly, inactivity of the system when the chloride anions in the buffer were exchanged for gluconate–a large hydrophilic anion too hydrophilic to be transported–further confirmed that the transport in the presence of chloride is cation independent, occurring via Cl^−^/OH^−^ antiport (or Cl^−^/H^+^ symport) and not via a cation‐dependent H^+^/Na^+^ antiport mechanism (Figure S70). Finally, an antiport mechanism of transport was validated for Cl^‒^/ NO_3_^‒^ exchange in lucigenin‐based fluorescence assays by exchanging the nitrate anion for the more hydrophilic dianionic sulfate. In this assay, lucigenin was encapsulated within the liposomes containing NaCl (200 mM) buffered at pH 6.5, and the ion transport activity was monitored with either NaNO_3_ (200 mM) or Na_2_SO_4_ (200 mM) in the external buffer. Significant transport activity was observed only with external nitrate, and not in the presence of the strongly solvated and poorly transported sulfate anion (Figure 3f). This suggests that Cl^‒^/NO_3_^‒^ antiport is the primary mechanism of ion transport rather than H^+^/Cl^‒^ symport, because the rate of the latter is not affected by the nature of the external anion.

Evidence for anion transport by **1** via a mobile carrier mechanism was obtained by conducting analogous experiments in dipalmitoyl phosphatidylcholine (DPPC) LUVs. Inactivity at 25 °C, below the gel–liquid phase transition temperature for DPPC (*T*_m_  = 41 °C), and restoration of activity at 45 °C, is indicative of a mobile carrier process, rather than transport mediated by self‐assembly of **1** into a membrane spanning channel, that activity of which would be typically expected to be independent of the lipid phase (Figure 3c).

### Synthesis and anion binding experiments of caged pro‐transporters 1a and 1b

**Scheme 2:**
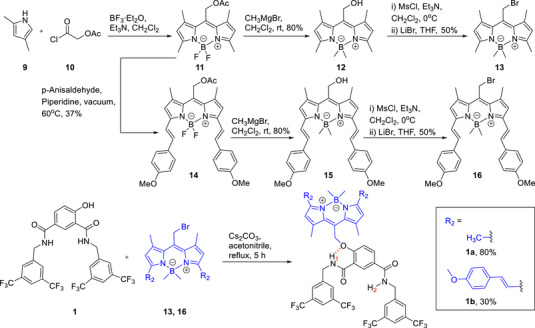
Chemical synthesis of caged pro‐transporters **1a** and **1b**.

Compound **1**, with the optimum anion binding and transport capabilities, was selected for caging with BODIPY photocleavable groups, which upon irradiation undergo photoheterolysis of the C─O(phenol) bond to release the anionophore.^[^ ^52^, ^53^ ^]^ To synthesize the corresponding BODIPY‐caged pro‐transporters **1a** and **1b**, bromo‐BODIPY derivatives **13** and **16** were initially synthesized (Scheme 2). Acetyl‐functionalized BODIPY **11** was obtained by treating 2,5‐dimethylpyrole **9** with acetoxyacetoyl chloride **10** in the presence of BF_3_·Et_2_O and triethyl amine. Treatment of compound **11** with *p*‐anisaldehyde afforded red‐shifted BODIPY derivative **14**.^[^ ^54^ ^]^ Treatment of acetyl BODIPY derivatives **11** and **14** with methyl magnesium bromide simultaneously methylated the boron atom and deprotected the acetyl group to afford **12** and **15**, respectively. Subsequent mesylation and reaction with lithium bromide yielded BODIPY‐bromo derivatives **13** and **16** in good yields. Finally, **13** and **16** were coupled with transporter **1** to furnish BODIPY‐caged pro‐transporters **1a** and **1b**, respectively (Scheme 2).

**Figure 4:**
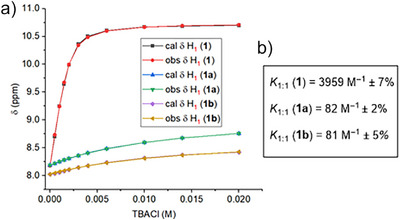
a) Change in chemical shift values of H_1_ upon titrating **1**, **1a**, or **1b** with tetrabutylammonium chloride in acetonitrile‐d_3_. b) Binding constant values of transporter **1** and caged pro‐transporters **1a** and **1b**, respectively.

With the caged anionophores **1a** and **1b** in hand, ^1^H NMR chloride binding experiments were performed in acetonitrile‐*d*_3_ to explore the effect of intramolecular hydrogen bonding of the amide protons on the chloride binding affinity. Both **1a** and **1b** displayed weak chloride binding with association constant values of 82 M^‒1^ ± 2% for **1a** and 81 M^‒1^ ± 5% for **1b**, respectively (Figures 4a,b and S58–S61), in contrast to strong anion binding by transporter **1** (*K*_a_(1:1)/Cl^‒^ = 3959 M^‒1^ ± 7%). These results demonstrate the role of the BODIPY cage in **1a** and **1b** in inhibiting chloride binding through the formation of the intramolecular hydrogen bond (Figure 1a), which renders the adjacent amide proton unavailable for anion recognition. This was supported by ^1^H‐^1^H NOESY NMR conformation analysis (Figure S37).

### Photo‐Responsive Ion Transport Activation

**Figure 5:**
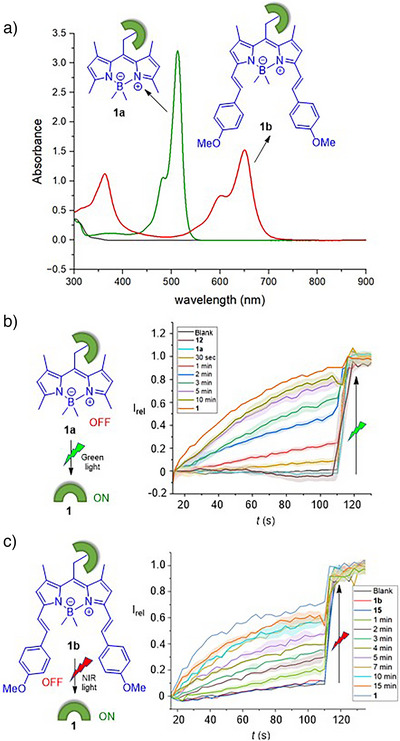
a) UV–vis absorption spectra of BODIPY‐caged pro‐transporters **1a** and **1b**. b) Ion transport activities across POPC LUVs containing HPTS, by photo‐irradiating **1a** (1.3 mol%) at 530 nm light using an LED (480 mW), and c) **1b** (0.67 mol%) at 730 nm light using an LED (1130 mW). Error bars represent standard deviations (*n* = 3).

The photo‐decaging of pro‐transporters **1a** and **1b** to generate **1** was initially performed in solution‐phase experiments via ^1^H NMR spectroscopy. UV‒vis absorption spectra of **1a** and **1b** displayed *λ*_max_ centered at 500 nm for **1a** and 645 nm for **1b**, respectively (Figure 5a). In the case of the latter, the BODIPY absorption extends out into the near‐IR region, up to ∼750 nm, suggesting that photo‐cleavage by irradiation in this region should be achievable. For **1a**, a 1 mM solution of the pro‐transporter in DMSO‐*d*_6_ was subjected to photoirradiation using a 530 nm LED (480 mW) and analyzed by ^1^H NMR experiments (Figure S75). Under these conditions, **1a** was readily cleaved with a half‐life of 415 s (Figure S76). For **1b**, photo‐cleavage could be achieved using both 625 nm (920 mW) and 730 nm (1130 mW) light with half‐lives of 120 and 185 min, respectively (Figures S77–S80).

Having confirmed successful decaging of pro‐transporters **1a** and **1b**, photo‐activation of ion transport was explored using pH dissipation anion transport assays in LUVs containing HPTS. Vesicles were prepared containing **1a** or **1b** pre‐incorporated into the membrane and then photo‐irradiated prior to initiation of transport by the addition of the NaOH base pulse. Vesicles containing **1a** were irradiated with 530 nm light, and those containing **1b** were irradiated with either 625 or 730 nm light for different time intervals, and the transport activity was monitored over time (Figure 5b,c). Photo‐activation of **1a** (1.3 mol% with respect to lipid) with the 530 nm LED resulted in efficient activation, achieving comparable activity to a sample of **1** after 10 min of irradiation, indicative of near quantitative decaging under these conditions (Figure 5b). Similarly, photo‐activation of **1b** (0.67 mol%) using the 625 and 730 nm LEDs resulted in efficient activation after 10 min of irradiation with 625 nm, and 15 min with 730 nm, respectively (Figures 5c and S82). In contrast, samples of caged anionophores **1a** and **1b** that were not irradiated did not show any activity at these concentrations. Analogous ion transport experiments using hydroxyl‐BODIPY derivatives **12** and **15**, which are generated as by‐products upon photo‐irradiation of **1a** and **1b**, confirmed that, as expected, these compounds do not mediate ion transport. Moreover, irradiation of a control BODIPY compound **C1** that released 4‐methoxyphenol (as a model of the ionophore but unable to facilitate ion transport) did not result in observable transport (Figure S83). Together, these experiments confirm that the observed ion transport triggered by photoirradiation of **1a** and **1b** is due to the formation of active transporter **1**, and not the BODIPY photo‐cleavage byproducts.

### NIR Photo‐Triggered Anticancer Activity

**Figure 6:**
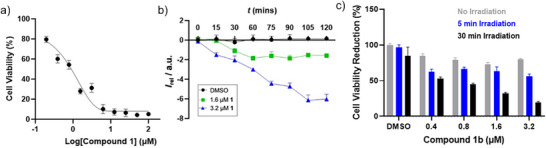
a) Dose‐response curve for compound **1** in *MDA‐MB‐231* cells following 4 h incubation, assessed via alamarblue. IC_50_ ≈ 1.2 µM. b) Chloride transport activity of **1** was measured using the MQAE fluorescence assay in MDA‐MB‐231 cells following 4 h incubation. Decreasing relative fluorescence *I*_rel_ corresponds to chloride influx. Cells were treated with 1.6 µM (green) or 3.2 µM (blue) transporter, or DMSO (black). c) Cell viability with respect to a DMSO control following treatment with pro‐transporter **1b** under different irradiation conditions (no irradiation; 5 or 30 min at 730 nm). Data represent mean ± SEM (*n* = 4).

Given the ability of certain chloride anionophores to induce apoptosis, we conducted preliminary cell viability experiments with **1b** to establish whether NIR‐activated anion transport is viable for inducing cell death, as a new approach to NIR photodynamic therapy. To this end, we initially investigated the cytotoxic effect of decaged anionophore **1** using *MDA‐MB‐231* cells, a model of late‐stage breast cancer. The cells were treated with increasing concentrations of **1** for 4 h, and viability was assessed using alamarBlue. These experiments revealed potent cytotoxicity, with an IC_50_ of ∼1.3 µM (Figure 6a), which is consistent with the compound's chloride anion transport activity. In contrast, analogous experiments with BODIPY‐caged pro‐transporter **1b** revealed that this photocaged analogue exhibited significantly reduced cytotoxicity (IC_50_ ∼15 µM, Figure S84). We confirmed the ability of **1** to transport chloride across cell membranes using a cell‐permeable chloride‐sensitive dye *N*‐(ethoxycarbonylmethyl)‐6‐methoxyquinolinium bromide (MQAE) which is quenched by chloride. A significant dose‐dependent quenching of the MQAE fluorescence was observed upon administering **1** to the cells (Figure 6b). Addition of **1b** did not cause chloride influx, but this could be switched on by irradiating the cells incubated with **1b** with a 730 nm LED (1130 mW) for 30 min (Figure S85). This is indicative of enhanced chloride uptake into the cells in the presence of **1**, consistent with results from the experiments in vesicles

To investigate whether NIR activation could trigger cytotoxicity via uncaging of the active transporter, cells were treated with **1b** and irradiated with the 730 nm LED for either 5 or 30 min. This led to a dramatic reduction in cell viability, which was dependent on the length of irradiation time (Figure 6c). In the presence of 3.2 µM **1b**, cell viability reduced by 18% and 72% relative to a DMSO control after 5 and 30 min of NIR irradiation, respectively. To confirm that the observed effects were attributable to the photo‐triggered release of anionophore **1**, control experiments were performed with the BODIPY photo‐deprotection byproduct **15**. Treatment with **15** resulted in minimal cytotoxicity below 3 µM (Figure S86). Irradiation of the cells with 730 nm light in the absence of compound had no significant effect on viability (Figure S87). These results suggest that the cytotoxicity of irradiated **1b** stems from the generation of **1**, rather than the byproduct or light exposure alone. Together, these experiments demonstrate that the NIR‐triggered release of an active anion transporter from a photoresponsive pro‐transporter leads to significant cancer cell death.

## Conclusion

In conclusion, we report the first examples of photo‐responsive transmembrane anion transporters that are activated with NIR light in the therapeutic window (∼600–1000 nm). We utilized the dynamic hydrogen bonding interactions of 4‐hydroxyisophthalamide anionophores, caged with a  π‐conjugated extended BODIPY photo‐cage. Irradiation with NIR light removes the cage, triggering a rearrangement of the anionophore hydrogen bonding and activation of chloride binding and transport. Experiments in cancer cells revealed that NIR activation of the ionophore triggered near‐IR light‐dependent chloride influx and triggered cell death. Unlike existing photodynamic therapies that generate reactive oxygen species, which can be impeded by tumor hypoxia, this approach uses oxygen‐independent photo‐triggered ion transport to induce cell death. We anticipate that the ability to use near‐IR light to activate ionophores may therefore open up a new approach toward photo‐dynamic therapy applications.

## Conflict of Interests

The authors declare no conflict of interest.

## Supporting information



Supporting Information

## Data Availability

The data that support the findings of this study are available in the Supporting Information of this article.
