# Supplementary material for: Near‐Infrared Triggered Anion Transport Induces Cancer Cell Death
Source: Angew Chem Int Ed Engl. 2025 Dec 12;65(5):e23734. doi: 10.1002/anie.202523734 (PMC12851001; doi:10.1002/anie.202523734)
Supplement: Supplementary file 1 — Supporting Information [file ANIE-65-e23734-s001.pdf]

Supporting Information for

Near-Infrared-Triggered Anion Transport Induces Cancer Cell Death

Manzoor Ahmad[a], Ríona M. Devereux[a], Angela J. Russell[a,b] and Matthew J. Langton*[a]

I. **Materials and methods** 2
II. **Synthesis** 3
III. **NMR Spectra** 13
IV. **HRMS Spectra:** 31
V. **Anion Binding Studies** 38
VI. **Ion transport studies** 44
VII. **Photoresponsive studies** 53
VIII. **Stimulus-responsive ion transport activation** 57
IX. **Biological studies** 59
X. **References** 61

I. Materials and methods

All reagents and solvents were purchased from commercial sources and used without further purification. Lipids were purchased from Avanti Polar Lipids and used without further purification. Column chromatography was carried out on Merck® silica gel 60 under a positive pressure of nitrogen. Where mixtures of solvents were used, ratios are reported by volume. NMR spectra were recorded on a Bruker AVIII 400, Bruker AVII 500 (with cryoprobe), and Bruker AVIII 500 spectrometers. Chemical shifts are reported as δ values in ppm. Mass spectra were carried out on a Waters Micromass LCT and Bruker microTOF spectrometers. UV-Vis spectra were recorded on a V-770 UV-Visible/NIR Spectrophotometer equipped with a Peltier temperature controller and stirrer, using quartz cuvettes of 1 cm path length. Fluorescence spectroscopic data were recorded using an Agilent fluorescence spectrophotometer, equipped with Peltier temperature controller and stirrer. Experiments were conducted at 25°C unless otherwise stated. Vesicles were prepared as described below using the Avestin "LiposoFast" extruder apparatus, equipped with polycarbonate membranes with 200 nm pores. GPC purification of vesicles was carried out using GE Healthcare PD-10 desalting columns prepacked with Sephadex G 25 medium. Photoirradiation was carried out using Thorlabs-mounted LEDs M730L6 (1130mW), M625L4 (920 mW), and M530L4 (480 mW), respectively.

Abbreviations

DCM: Dichloromethane; **DMF:** *N*, *N*-Dimethylformamide; **THF:** Tetrahydrofuran; **DMSO:** Dimethylsulfoxide; **DPPC:** 1,2-dipalmitoyl-sn-glycero-3-phosphocholine; **EYPG:** egg-yolk phosphatidylglycerol; **HRMS:** High resolution mass spectrometry; **LUVs:** large unilamellar vesicles; **MeOH:** Methanol; **POPC:** 1-palmitoyl-2-oleoyl-sn-glycero-3-phosphocholine; rt: Room temperature; **TBACl:** Tetrabutylammonium chloride.

II. Synthesis

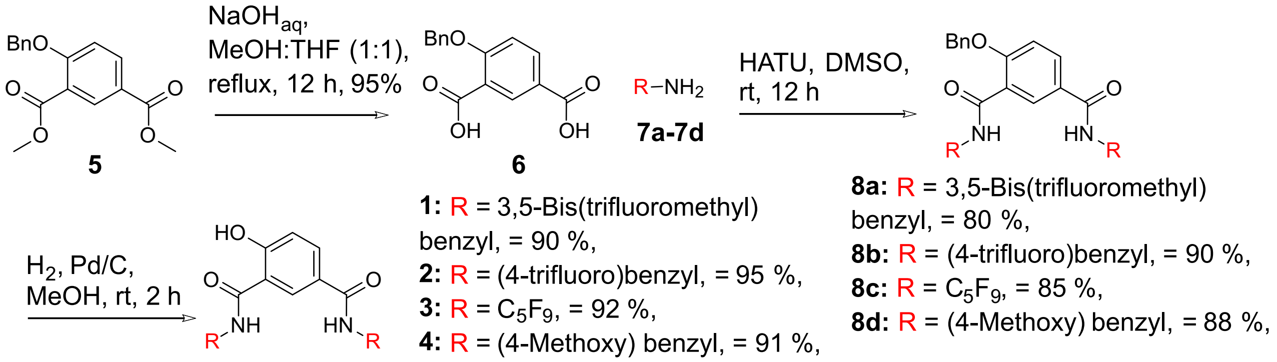

Scheme 1. Chemical synthesis of transporters **1-4.**

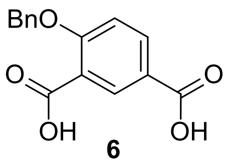

6

4-(benzyloxy)isophthalic acid (6): In a 25 mL round-bottom flask, dimethyl 4-(benzyloxy)isophthalate **5** (2 g, 6.6 mmol) was dissolved in a mixture of Methanol (20 mL), tetrahydrofuran (20 mL), and aqueous sodium hydroxide (2 N, 20 mL). The reaction was refluxed for 12 h at 70 °C. After the completion of the reaction, monitored by TLC, the solvent was removed under rotary evaporator. The mixture was then acidified by the addition of HCl (2 N) dropwise. The white precipitate formed was collected by vacuum filtration, washed with diethyl ether and pentane to furnish the compound **6** in excellent yield. Yield: 95%, 1.7 g. **^{1}H NMR (400 MHz, DMSO) δ** 12.90 (s, 2H), 8.24 (d, J = 2.3 Hz, 1H), 8.05 (dd, J = 8.8, 2.3 Hz, 1H), 7.53 – 7.49 (m, 2H), 7.43 – 7.38 (m, 2H), 7.36 – 7.29 (m, 2H), 5.30 (s, 2H); **^{13}C NMR (151 MHz, DMSO)** δ 167.0, 166.8, 160.8, 136.9, 134.6, 132.6, 128.8, 128.2, 127.59, 123.1, 122.0, 114.1, 70.2; **HRMS (ESI) *m/z*:** Calcd. for $C_{15}H_{12}O_5Na$ $[M+Na]^+$, 295.0577; Found 295.0577.

General procedure for the synthesis of compounds 8a-8d:

In a 25 mL round-bottom flask, 4-(benzyloxy)isophthalic acid **6** (200 mg, 0.73 mmol, 1 eq) and HATU (559 mg, 1.47 mmol, 2 eq) were dissolved in DMSO (10 mL). The resulting solution was stirred at room temperature for 30 min. After 30 min, the aliphatic amine (2.2 eq) and triethylamine (212 mL, 1.62 mmol, 2.2 eq) were added. The reaction mixture was stirred at room temperature for an additional 12 h. After completion of the reaction, 10 mL of water was added to the reaction mixture. The white precipitate formed for **8b** and **8d** was filtered under vacuum filtration, washed with diethyl ether and pentane to give **8b** and **8d** in excellent yields. For **8a** and **8c** reaction mixture was extracted with ethyl acetate. The organic layer was washed with brine, dried over magnesium sulfate, and purified by column chromatography.

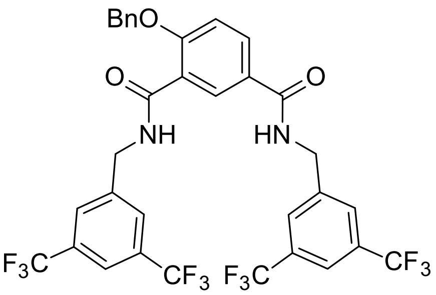

8a

4-(benzyloxy)-N1, N3-bis(3,5-bis(trifluoromethyl)benzyl)isophthalamide (8a): This compound was synthesized by reacting 4-(benzyloxy)isophthalic acid **6** (200 mg, 0.73 mmol, 1 eq) with (3,5-bis(trifluoromethyl)phenyl) methanamine **7a** (357 mg, 1.47 mmol, 2 equiv). The product was purified via silica gel column chromatography (*Eluent*: 30% ethyl acetate in hexane). Yield: 80%, 424 mg. **^{1}H NMR (400 MHz, DMSO)** δ 9.20 (t, *J* = 5.9 Hz, 1H), 9.00 (t, *J* = 6.1 Hz, 1H), 8.22 (d, *J* = 2.4 Hz, 1H), 8.06 – 7.94 (m, 7H), 7.42 (dd, *J* = 6.7, 2.9 Hz, 2H), 7.35 – 7.24 (m, 4H), 5.35 (s, 2H), 4.66 (dd, *J* = 18.3, 5.9 Hz, 4H); **^{13}C NMR (151 MHz, DMSO)** δ 165.9, 158.4, 143.8, 143.4, 136.6, 131.6, 130.9, 130.7, 130.5, 130.3, 129.7, 128.7, 128.3, 127.6, 126.5, 124.7-127.6 (quartet), 124.4, 122.9-122.8 (quartet), 113.8, 70.3, 42.5, 42.5; **HRMS (ESI) *m/z*:** Calcd. for $C_{33}H_{23}F_{12}N_2O_3$ $[M+H]^+$, 723.1512; Found 723.1507.

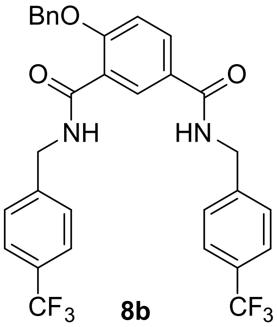

8b

4-(benzyloxy)-N1, N3-bis(4-(trifluoromethyl)benzyl)isophthalamide (8b): This compound was synthesized by reacting 4-(benzyloxy)isophthalic acid **6** (200 mg, 0.73 mmol, 1 equiv) with (4-(trifluoromethyl)phenyl)methanamine**7b** (257 mg, 1.47 mmol, 2 equiv). Yield: 90%, 387 mg. **^{1}H NMR (400 MHz, DMSO)** δ 9.16 (t, *J* = 6.0 Hz, 1H), 8.84 (t, *J* = 6.1 Hz, 1H), 8.22 (d, *J* = 2.4 Hz, 1H), 8.01 (dd, *J* = 8.7, 2.4 Hz, 1H), 7.70 (d, *J* = 7.9 Hz, 2H), 7.58 – 7.41 (m, 8H), 7.35 (d, *J* = 7.3 Hz, 4H), 5.31 (s, 2H), 4.55 (d, *J* = 5.9 Hz, 4H); **^{13}C NMR (151 MHz, DMSO)** δ 165.8, 165.7, 158.3, 145.1, 144.5, 136.6, 131.4, 129.6, 128.9, 128.5, 128.4, 128.3, 128.1, 128.1-127.5 (2 quartets), 127.5-127.4 (2 quartets) 126.78, 124.93, 113.49, 70.66, 42.81, 42.73; **HRMS (ESI) *m/z*:** Calcd. for $C_{24}H_{19}F_6N_2O_3$ $[M+H]^+$, 587.1764; Found 587.1763.

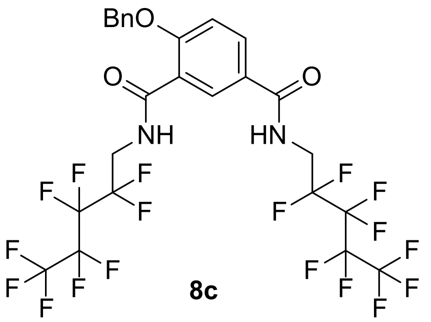

4-(benzyloxy)-N1, N3-bis(2,2,3,3,4,4,5,5,5-nonafluoropentyl)isophthalamide (8c): This compound was synthesized by reacting 4-(benzyloxy)isophthalic acid **6** (200 mg, 0.73 mmol, 1 equiv) with 2,2,3,3,4,4,5,5,5-nonafluoropentan-1-amine **7c** (365 mg, 1.47 mmol, 2 equiv). The product was purified via silica gel column chromatography (*Eluent*: 20% ethyl acetate in hexane). Yield: 85%, 458 mg. **^{1}H NMR (600 MHz, DMSO)** δ 9.10 (t, *J* = 6.3 Hz, 1H), 8.80 (t, *J* = 6.4 Hz, 1H), 8.23 (d, *J* = 2.3 Hz, 1H), 8.02 (dd, *J* = 8.8, 2.4 Hz, 1H), 7.51 – 7.47 (m, 2H), 7.39 – 7.31 (m, 4H), 5.34 (s, 2H), 4.19 (dtd, *J* = 22.4, 16.5, 6.2 Hz, 4H); **^{13}C NMR (151 MHz, DMSO)** δ 166.3, 166.2, 158.8, 136.48, 132.2, 130.2, 128.8, 128.5, 128.0, 125.9, 123.6, 113.8, 70.6, 38.9, 38.75; **HRMS (ESI) *m/z*:** Calcd. for $C_{25}H_{17}F_{18}N_2O_3$ $[M+H]^+$, 735.0946; Found 735.0941.

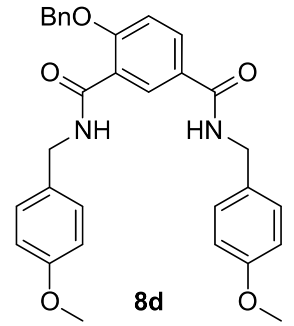

4-(benzyloxy)-N1, N3-bis(4-methoxybenzyl)isophthalamide (8d): This compound was synthesized by reacting 4-(benzyloxy)isophthalic acid **6** (200 mg, 0.73 mmol, 1 equiv) with (4-methoxyphenyl)methanamine**7d** (201 mg, 1.47 mmol, 2 equiv). Yield: 88%, 330 mg. **^{1}H NMR (600 MHz, DMSO)** δ 8.97 (t, *J* = 6.0 Hz, 1H), 8.60 (t, *J* = 5.9 Hz, 1H), 8.22 (d, *J* = 2.4 Hz, 1H), 7.97 (dd, *J* = 8.7, 2.4 Hz, 1H), 7.46 – 7.42 (m, 2H), 7.38 – 7.34 (m, 3H), 7.31 (d, *J* = 8.8 Hz, 1H), 7.25 – 7.22 (m, 2H), 7.18 – 7.14 (m, 2H), 6.90 – 6.86 (m, 2H), 6.79 – 6.76 (m, 2H), 5.27 (s, 2H), 4.39 (d, *J* = 5.9 Hz, 4H), 3.72 (s, 6H); **^{13}C NMR (151 MHz, DMSO)** δ 165.4, 165.3, 158.6, 158.6, 158.2, 136.6, 132.2, 131.4, 131.3, 129.8, 129.1, 128.9, 128.9, 128.4, 128.2, 127.1, 124.7, 114.1, 114.1, 113.4, 70.6, 55.5, 55.5, 42.5, 42.5; $[M+H]^+$, 511.2227; Found 511.2231.

General procedure for the synthesis of compounds 1-4:

In a 25 mL round-bottom flask, **8a-8d** (200 mg) was dissolved in THF: Methanol (2:1, 30 mL) to which 10% Pd/C (0.02 mg) was added. The reaction was subjected to stirring for a duration of 3 h under hydrogen (1 Atm). Following the reaction, the Pd/C was filtered out, and the resulting filtrate was collected and dried under vacuum, washed with diethyl ether and pentane to furnish compounds **1-4** in excellent yields.

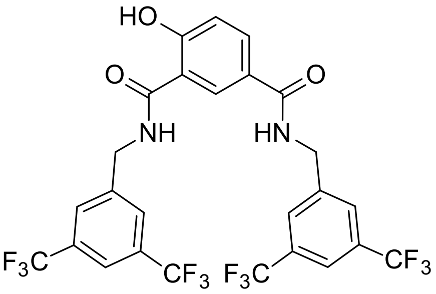

1

N1, N3-bis(3,5-bis(trifluoromethyl)benzyl)-4-hydroxyisophthalamide (**1**): The compound **1** was obtained by reducing 4-(benzyloxy)-N1, N3-bis(3,5-bis(trifluoromethyl)benzyl) isophthalamide **8a** with H_2 over 10% Pd/C as a white solid (157 mg, 90%); **^{1}H NMR (600 MHz, DMSO)** δ 12.40 (d, *J* = 2.2 Hz, 1H), 9.47 (t, *J* = 6.0 Hz, 1H), 9.10 (t, *J* = 5.9 Hz, 1H), 8.43 (d, *J* = 2.2 Hz, 1H), 8.06 (s, 2H), 8.03 – 7.97 (m, 4H), 7.94 (dd, *J* = 8.6, 2.2 Hz, 1H), 7.03 (d, *J* = 8.6 Hz, 1H), 4.67 (dd, *J* = 37.0, 5.8 Hz, 4H); **^{13}C NMR (151 MHz, DMSO)** δ 168.3, 168.3, 166.2, 161.8, 143.8, 143.2, 132.7, 130.9-130.2 (double quartets), 128.8-128.6 (double quartets), 129.2, 126.5, 125.1, 124.7, 122.9, 117.5, 116.7, 42.5, 42.4; **^{13}C-{^{19}F} (126 MHz, DMSO) δ** 168.3, 166.2, 161.8, 143.8, 143.2, 132.7, 130.6, 130.6, 129.3, 128.7, 128.6, 125.1, 123.8, 123.8, 123.8, 123.6, 121.2, 121.0, 117.5, 116.8, 42.5, 42.4; **^{19}F NMR (565 MHz, DMSO)** δ -61.36.; **HRMS (ESI) *m/z*:** Calcd. for $C_{26}H_{17}F_{12}N_2O_3$ $[M+H]^+$, 633.1042; Found 633.1058.

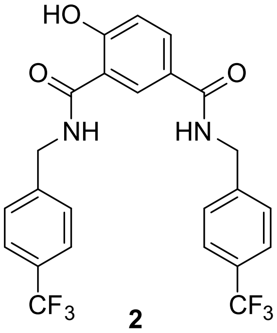

2

4-hydroxy-N1,N3-bis(4-(trifluoromethyl)benzyl)isophthalamide (**2**): The compound **2** was obtained by reducing 4-(benzyloxy)-N1,N3-bis(4-(trifluoromethyl)benzyl)isophthalamide **8b** with H_2 over 10% Pd/C as a white solid (160 mg, 95%); **^{1}H NMR (600 MHz, DMSO)** δ 12.66 (s, 1H), 9.53 (t, *J* = 6.1 Hz, 1H), 9.01 (t, *J* = 6.0 Hz, 1H), 8.48 (d, *J* = 2.2 Hz, 1H), 7.95 (dd, *J* = 8.6, 2.2 Hz, 1H), 7.70 (t, *J* = 8.3 Hz, 4H), 7.55 (dd, *J* = 17.1, 7.9 Hz, 4H), 7.01 (d, *J* = 8.6

Hz, 1H), 4.58 (dd, J = 34.1, 5.9 Hz, 4H); **^{13}C NMR (151 MHz, DMSO)** δ 168.7, 166.1, 162.4, 145.1, 144.3, 132.8, 128.9, 128.7-128.4 (doublet of quartets), 128.4, 128.3, 128.0-127.4 (doublet of quartets), 125.7-125.5 (doublet of quartets), 125.3, 123.9, 123.8, 117.5, 116.0, 42.8, 42.7; **^{13}C-{^{19}F} (126 MHz, DMSO) δ** 168.6, 166.1, 162.4, 145.1, 144.3, 132.8, 129.0, 128.4, 128.3, 128.0, 127.9, 125.7, 125.6, 125.3, 124.8, 124.8, 117.5, 116.0, 42.8, 42.6; **^{19}F NMR (565 MHz, DMSO)** δ -61.19.**HRMS (ESI) *m/z*:** Calcd. for $C_{24}H_{19}F_6N_2O_3$ $[M+H]^+$, 497.1294; Found 497.1307.

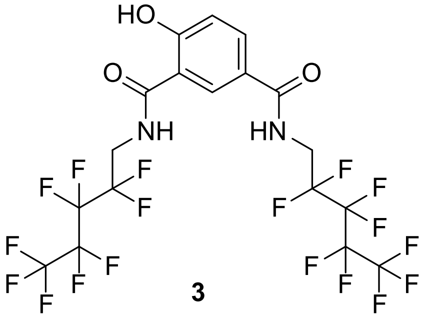

3

4-hydroxy-N1, N3-bis(2,2,3,3,4,4,5,5,5-nonafluoropentyl)isophthalamide (**3**): The compound **3** was obtained by reducing 4-(benzyloxy)-N1, N3-bis(2,2,3,3,4,4,5,5,5-nonafluoropentyl)isophthalamide **8c** with H_2 over 10% Pd/C as a white solid (161 mg, 92%); **^{1}H NMR (600 MHz, DMSO)** δ 12.16 (s, 1H), 9.16 (t, J = 6.3 Hz, 1H), 9.00 (t, J = 6.2 Hz, 1H), 8.47 (d, J = 2.3 Hz, 1H), 7.96 (dd, J = 8.6, 2.3 Hz, 1H), 7.06 (d, J = 8.6 Hz, 1H), 4.23 (dtd, J = 64.7, 16.5, 6.2 Hz, 5H); **^{13}C NMR (151 MHz, DMSO)** δ 167.7, 166.6, 161.1, 133.2, 130.3, 124.8, 117.5, 117.0; **^{13}C-{^{19}F} (126 MHz, DMSO) δ** 167.7, 166.6, 161.1, 133.2, 130.3, 124.7, 117.5, 117.3, 117.1, 116.2, 116.1, 110.7, 110.6, 108.6, 39.0, 38.8; **^{19}F NMR (565 MHz, DMSO)** δ -116.76, -124.07, -125.65; **HRMS (ESI) *m/z*:** Calcd. for $C_{18}H_{11}F_{18}N_2O_3$ $[M+H]^+$, 645.0477; Found 645.0483.

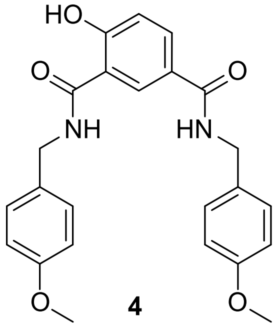

4

4-hydroxy-N1, N3-bis(4-methoxybenzyl)isophthalamide (**4**): The compound **4** was obtained by reducing 4-(benzyloxy)-N1, N3-bis(4-methoxybenzyl)isophthalamide **8c** with H_2 over 10% Pd/C as a white solid (149 mg, 91%); **^{1}H NMR (600 MHz, DMSO)** δ 9.64 (s, 1H), 8.76 (t, J = 5.9 Hz, 1H), 8.45 (d, J = 2.3 Hz, 1H), 7.87 (dd, J = 8.6, 2.2 Hz, 1H), 7.33 – 7.15 (m, 4H), 6.89 (ddd, J = 9.9, 8.6, 3.2 Hz, 5H), 4.42 (dd, J = 33.4, 5.2 Hz, 4H), 3.72 (d, J = 2.7 Hz, 6H); **^{13}C NMR (151 MHz, DMSO)** δ 168.5, 165.9, 158.7, 158.6, 132.5, 132.2, 131.4, 129.2, 129.0, 128.8, 117.8, 116.0, 114.2, 114.1, 55.5, 55.5, 42.5, 42.3; **HRMS (ESI) *m/z*:** Calcd. for $C_{24}H_{25}N_2O_5$ $[M+H]^+$, 421.1758; Found 421.1759.

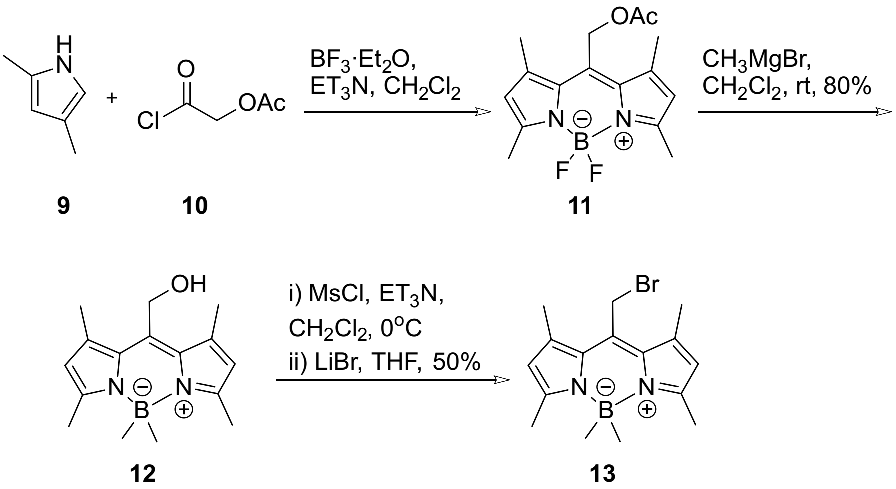

Scheme 2: Synthesis of bromo-BODIPY derivative **13.**

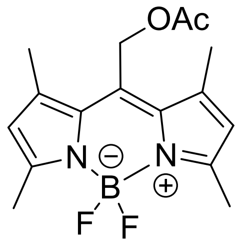

11

(5,5-difluoro-1,3,7,9-tetramethyl-5H-4l4,5l4-dipyrrolo[1,2-c:2',1'-f][1,3,2]diazaborinin-10-yl)methyl acetate (11): In a 50 mL round bottomed flask, 2-Chloro-2-oxoethyl acetate **10** (790 µL, 0.730 mmol) was added to a mixture of 2,4-dimethyl pyrrole **9** (0.230 mL, 2.20 mmol) and triethylamine Et_3N (0.410 mL, 2.93 mmol) in dry DCM (5 mL). Note: addition generates heat, so it must be done slowly, dropwise. After that, $BF_3{\cdot}Et_2O$ (0.550 mL, 4.39 mmol) was added slowly to the reaction mixture, and the reaction was further allowed to stir for 15 minutes. After that, silica was added to RB, and the solvent was evaporated to make the slurry. The crude mixture was purified by column chromatography using pentane/Et_2O (2:1; v/v) as the eluent. The product was obtained as red-gold crystals (93 mg, 40% yield): **^{1}H NMR (400 MHz, $CDCl_3$)** δ 6.01 (s, 2H), 5.22 (s, 2H), 2.46 (s, 6H), 2.29 (s, 6H), 2.06 (s, 3H). ^{1}H NMR matches the reported literature[1].

Compound **12** was synthesized from **11** using the known reported literature[2]

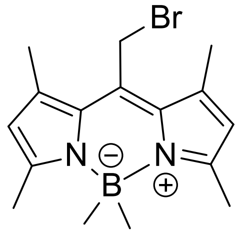

13

10-(bromomethyl)-1,3,5,5,7,9-hexamethyl-5H-4l4,5l4-dipyrrolo[1,2-c:2',1'-f][1,3,2]diazaborinine (13) :. Triethylamine (97 µL, 0.74 mmol, 2.0 equiv.) was charged

dropwise to a solution of **12** (100 mg, 0.37 mmol, 1.0 equiv.) in anhydrous CH_2Cl_2 (3.0 mL) at 0 °C. Mesyl chloride (42 µL, 0.55 mmol, 1.5 equiv.) was then added, and the solution was left to stir at 0 °C for 30 minutes, in the dark. The reaction mixture was allowed to warm to RT and was washed with saturated sodium hydrogen carbonate solution (5 mL) and brine (5 mL). The combined aqueous layers were back-extracted with CH_2Cl_2 (10 mL). The combined organic layers were dried over $MgSO_4$, filtered, and concentrated in vacuo. Lithium bromide (128 mg, 1.48 mmol, 4.0 equiv.) was charged to the resulting dark red solid, and the mixture was dissolved in anhydrous THF (5.0 mL). The solution was stirred for 25 h in the dark. The solvent was removed in vacuo, and the residue was dissolved in CH_2Cl_2 (30 mL). The solution was washed with water (25 mL) and brine (25 mL). The combined aqueous layers were back-extracted with CH_2Cl_2 (30 mL). The combined organic layers were dried over $MgSO_4$, filtered, and concentrated in vacuo. The crude mixture was purified by column chromatography using pentane/Et_2O (2:1; v/v) as the eluent. The product was obtained as red crystals (67 mg, 55% yield). **^{1}H NMR (400 MHz, $CDCl_3$)** δ 6.09 (s, 2H), 4.77 (s, 2H), 2.58 (s, 6H), 2.46 (s, 6H), 0.18 (d, J = 21.8 Hz, 6H). ^{1}H NMR matches with the reported literature[2].

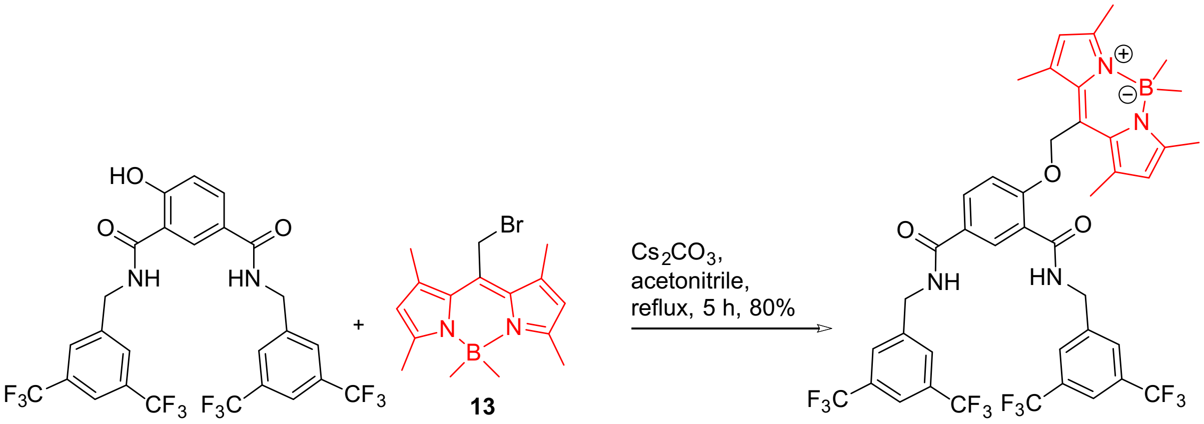

N1, N3-bis(3,5-bis(trifluoromethyl)benzyl)-4-((1,3,5,5,7,9-hexamethyl-5H-4l4,5l4-dipyrrolo[1,2-c:2',1'-f][1,3,2]diazaborinin-10-yl)methoxy)isophthalamide (1a): In a 50 mL round bottomed flask, **1** (100 mg, 0.158 mmol, 1 equiv) and Cs_2CO_3 (61 mg, 0.189 mmol, 1.2 equiv) were dissolved in acetonitrile (20 mL), followed by the addition of compound **13** (63 mg, 0.189 mmol, 1.2 equiv). The reaction mixture was stirred for 5 h at 70 °C using an oil bath. After the completion of the reaction, the solvent was evaporated through a rotary evaporator, and the crude mixture was extracted with ethyl acetate (3 × 30 mL). The organic layers were dried with anhydrous magnesium sulfate, filtered, and concentrated under reduced pressure to give the crude product. The crude residue was then purified by column chromatography over 100−200 mesh silica gel (*Eluent*: 30 ethyl acetate in hexane) to furnish **1a** as an orange solid (111 mg, 80%). **^{1}H NMR (600 MHz, DMSO)** δ 9.23 (t, J = 6.0 Hz, 1H), 8.77 (t, J = 6.0 Hz, 1H), 8.14 (d, J = 2.3 Hz, 1H), 8.08 (dd, J = 8.7, 2.4 Hz, 1H), 8.02 (d, J = 9.7 Hz, 3H), 7.95 (s, 1H), 7.89 (s, 2H), 7.51 (d, J = 8.8 Hz, 1H), 6.12 (s, 2H), 5.37 (s, 2H), 4.66 (d, J = 5.9 Hz, 2H), 4.52 (d, J = 6.0 Hz, 2H), 2.39 (s, 6H), 2.23 (s, 6H), 0.10 (s, 6H); **^{13}C NMR (151 MHz, DMSO)** δ 165.9, 165.8, 158.2, 153.1, 143.0, 137.8, 133.6, 131.6, 131.5, 130.9-130.3 (doublet of quartets), 129.4, 127.1, 126.5-121.0 (doublet of quartets), 124.9, 123.0, 113.0, 67.4, 63.1, 42.5,

42.2, 25.5, 16.6, 15.3; **^{19}F NMR (565 MHz, DMSO)** δ -61.30, -61.40; **HRMS (ESI) *m/z*:** Calcd. for $C_{42}H_{38}BF_{12}N_4O_3$ [M+H]$^+$, 885.2840; Found 885.2854.

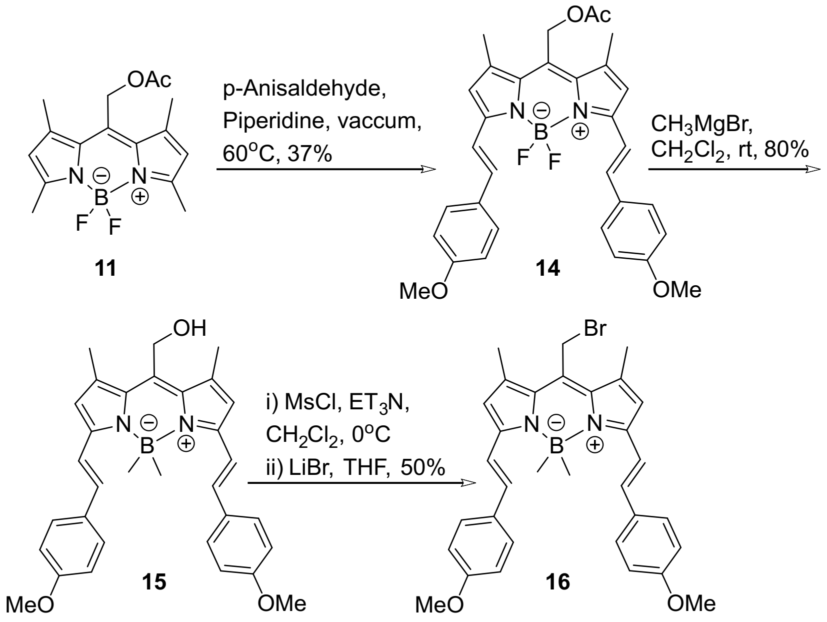

Scheme 3: Synthesis of red-shifted bromo-bodipy derivative **16.**

Compound **14** was synthesized from **11,** following the reported literature [3]

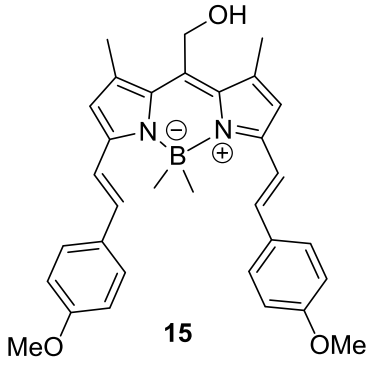

(3,7-bis((E)-4-methoxystyryl)-1,5,5,9-tetramethyl-5H-4l4,5l4-dipyrrolo[1,2-c:2',1'-f][1,3,2]diazaborinin-10-yl)methanol (15): To a solution of **14**, dissolved in dichloromethane, was added 15 equiv. of methyl magnesium bromide (4.5 mL 3 M solution in diethyl ether, 13.4 mmol, 15 equiv.). The solution was stirred for 1 h, after which the reaction was complete by TLC. The reaction was quenched with ammonium chloride, and ethyl acetate was added. The organic layer was washed 3 times with ammonium chloride, once with brine, and dried over sodium sulphate. The solvent was reduced under a vacuum, and the product was purified via silica gel column chromatography (*Eluent*: 2:1 diethyl ether: pentane) to give **11** as a red solid in an 80% yield (364 mg). **^{1}H NMR (400 MHz, $CDCl_3$)** δ 7.41 (d, *J* = 25.7 Hz, 6H), 7.00 (d, *J* = 16.3 Hz, 2H), 6.87 (d, *J* = 9.5 Hz, 5H), 6.65 (s, 2H), 4.92 (s, 2H), 3.79 (s, 6H), 2.52 (s, 5H), 0.38 (s, 6H). ^{1}H NMR matches the reported literature[3]

Compound **16** was synthesized from **15,** following the reported literature [3]

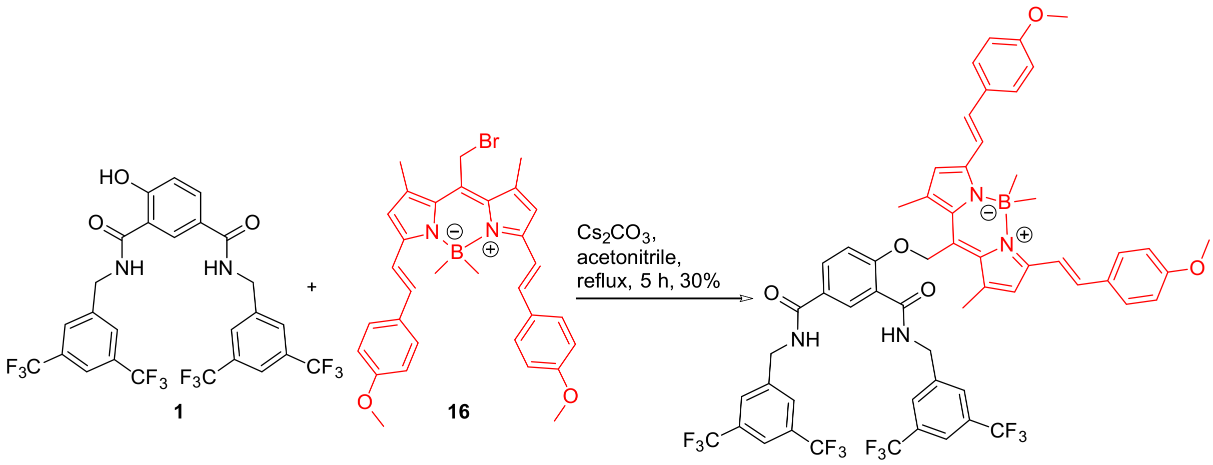

4-((3,7-bis((E)-4-methoxystyryl)-1,5,5,9-tetramethyl-5H-4l4,5l4-dipyrrolo[1,2-c:2',1'-f][1,3,2]diazaborinin-10-yl)methoxy)-N1,N3-bis(3,5 bis(trifluoromethyl) benzyl) isophthalamide (1b): In a 50 mL round-bottomed flask, **1** (200 mg, 0.316 mmol, 1 equiv) and Cs_2CO_3 (122 mg, 0.316 mmol, 1.2 equiv) were dissolved in acetonitrile (20 mL), followed by the addition of compound **16** (216 mg, 0.379 mmol, 1.2 equiv). The reaction mixture was stirred for 5 h at 70 °C using an oil bath. After the completion of the reaction, the solvent was evaporated through a rotary evaporator, and the crude mixture was extracted with ethyl acetate (3 × 30 mL). The organic layers were dried with anhydrous magnesium sulfate, filtered, and concentrated under reduced pressure to give the crude product, which was purified via silica gel column chromatography (*Eluent*: 2:1 diethyl ether: pentane) to furnish **1b** as a dark green solid (106 mg, 30%). **^{1}H NMR (400 MHz, DMSO)** δ 9.25 (s, 1H), 8.85 (s, 1H), 8.11 (d, *J* = 15.6 Hz, 2H), 8.03 (s, 3H), 7.90 (s, 3H), 7.56 (d, *J* = 8.4 Hz, 5H), 7.33 (s, 4H), 7.04 (d, *J* = 8.2 Hz, 4H), 6.91 (s, 2H), 5.42 (s, 2H), 4.67 (d, *J* = 5.5 Hz, 2H), 4.53 (d, *J* = 5.8 Hz, 2H), 3.81 (s, 6H), 2.32 (s, 6H), 0.36 (s, 6H); **^{13}C NMR (151 MHz, DMSO)** δ 170.8, 166.1, 165.9, 160.4, 158.2, 150.7, 143.8, 143.1, 137.3, 134.0, 133.6, 131.5, 130.9, 130.7, 130.6, 130.5, 130.3, 129.8, 129.3, 128.8, 127.1, 126.5, 126.4, 125.2, 124.7, 124.6, 122.9, 122.8, 119.4, 118.3, 115.1, 113.1, 60.2, 55.7, 21.2, 15.5, 14.5; **^{19}F NMR (565 MHz, $CDCl_3$)** δ -62.80, -62.80; **HRMS (ESI) *m/z*:** Calcd. for $C_{58}H_{50}BF_{12}N_4O_5$ $[M+H]^+$, 1121.3677; Found 1121.3669.

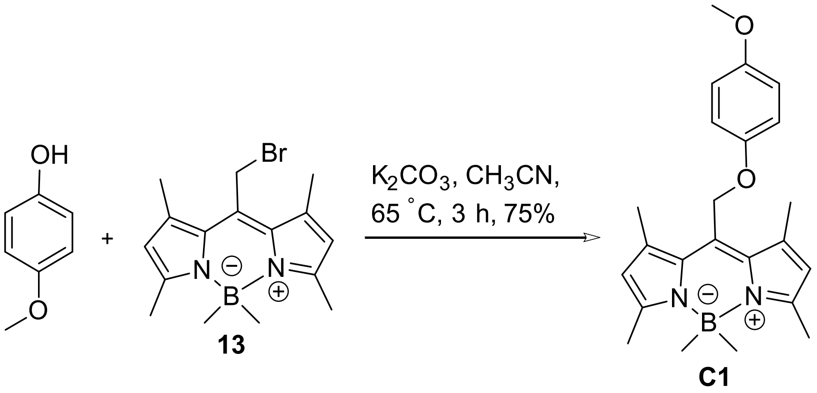

10-((4-methoxyphenoxy)methyl)-1,3,5,5,7,9-hexamethyl-5H-4l4,5l4-dipyrrolo[1,2-c:2',1'-f][1,3,2]diazaborinine (C1): In a 50 mL round-bottomed flask, 4-methoxy phenol (20 mg, 0.142 mmol, 1.0 equiv) and K_2CO_3 (18 mg, 0.142 mmol, 1.0 equiv) were dissolved in

acetonitrile (20 mL), followed by the addition of compound **13** (48 mg, 0.142 mmol, 1.0 equiv). The reaction mixture was stirred for 3 h at 65 °C using an oil bath. After the completion of the reaction, the solvent was evaporated through a rotary evaporator, and the crude mixture was extracted with ethyl acetate (3 × 30 mL). The organic layers were dried with anhydrous magnesium sulfate, filtered, and concentrated under reduced pressure to give the crude product, which was purified via silica gel column chromatography (*Eluent*:1:1 diethyl ether: pentane) to furnish **C1** as a red solid (40 mg, 75%). **^{1}H NMR (600 MHz, $CDCl_3$)** δ 6.97 (d, *J* = 9.2 Hz, 2H), 6.90 (d, *J* = 9.2 Hz, 2H), 6.09 (s, 2H), 5.18 (s, 2H), 3.81 (s, 3H), 2.48 (s, 6H), 2.34 (s, 6H), 0.24 (s, 6H); **^{13}C NMR (151 MHz, $CDCl_3$)** δ 153.2, 151.9, 151.5, 136.3, 133.2, 130.5, 121.4, 113.8, 113.8, 60.7, 54.7; **HRMS (ESI) *m/z*:** Calcd. for $C_{23}H_{29}BN_2O_2$ [M+H]$^+$, 377.2395; Found 377.2401.

III. NMR Spectra

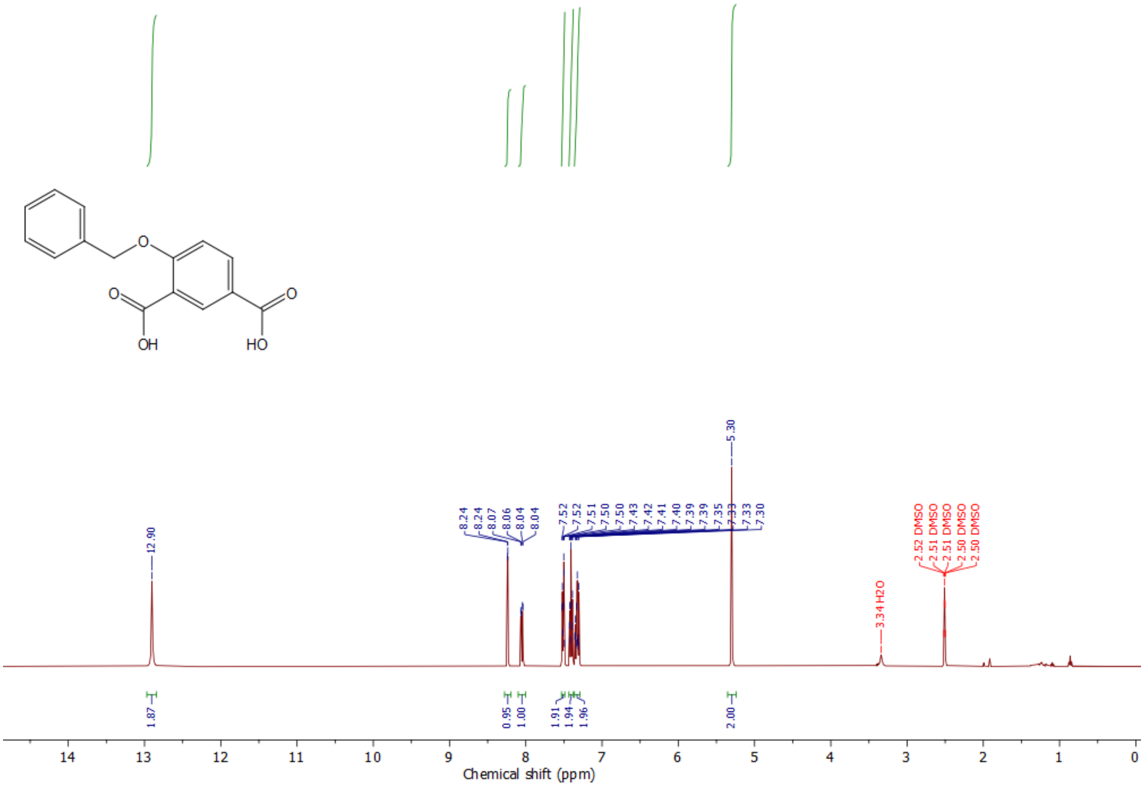

Figure S1: ^{1}H NMR spectrum of **6** in DMSO-d_6 (600 MHz, 298 K).

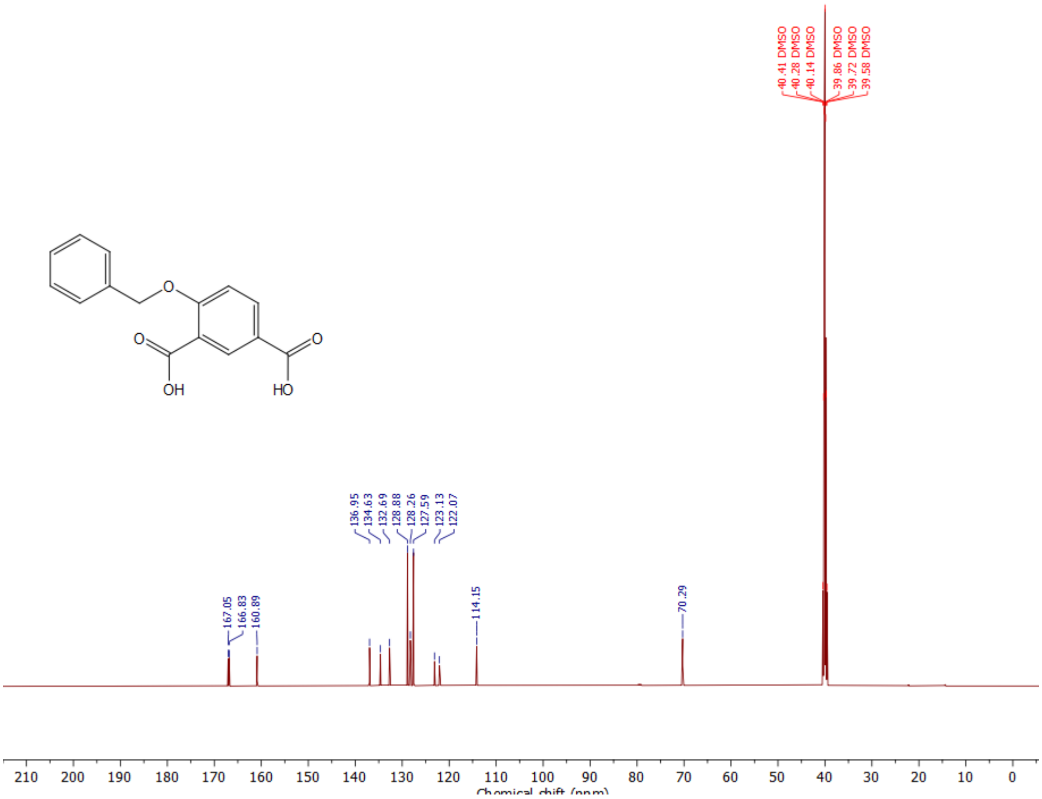

Figure S2: ^{13}C NMR spectrum of **6** in DMSO-d_6 (151 MHz, 298 K).

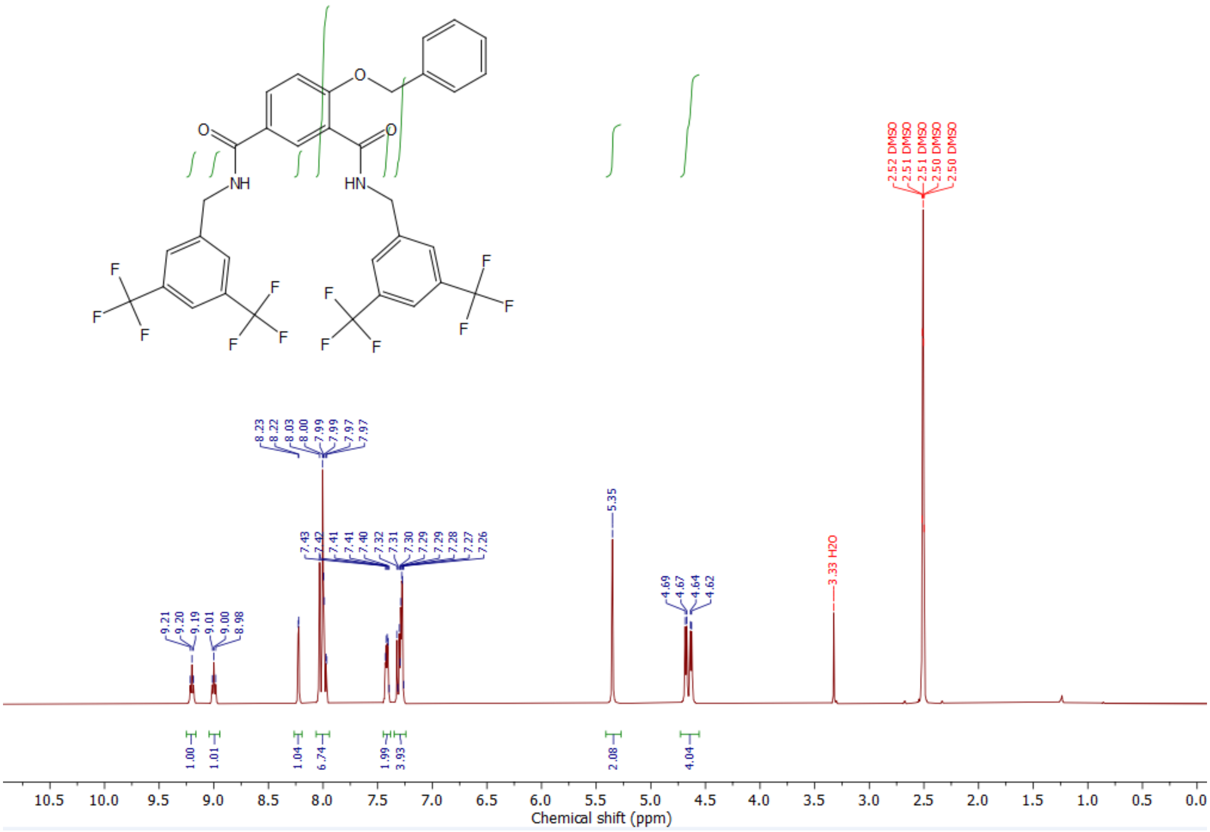

Figure S3: ^{1}H NMR spectrum of **8a** in DMSO-d_6 (600 MHz, 298 K).

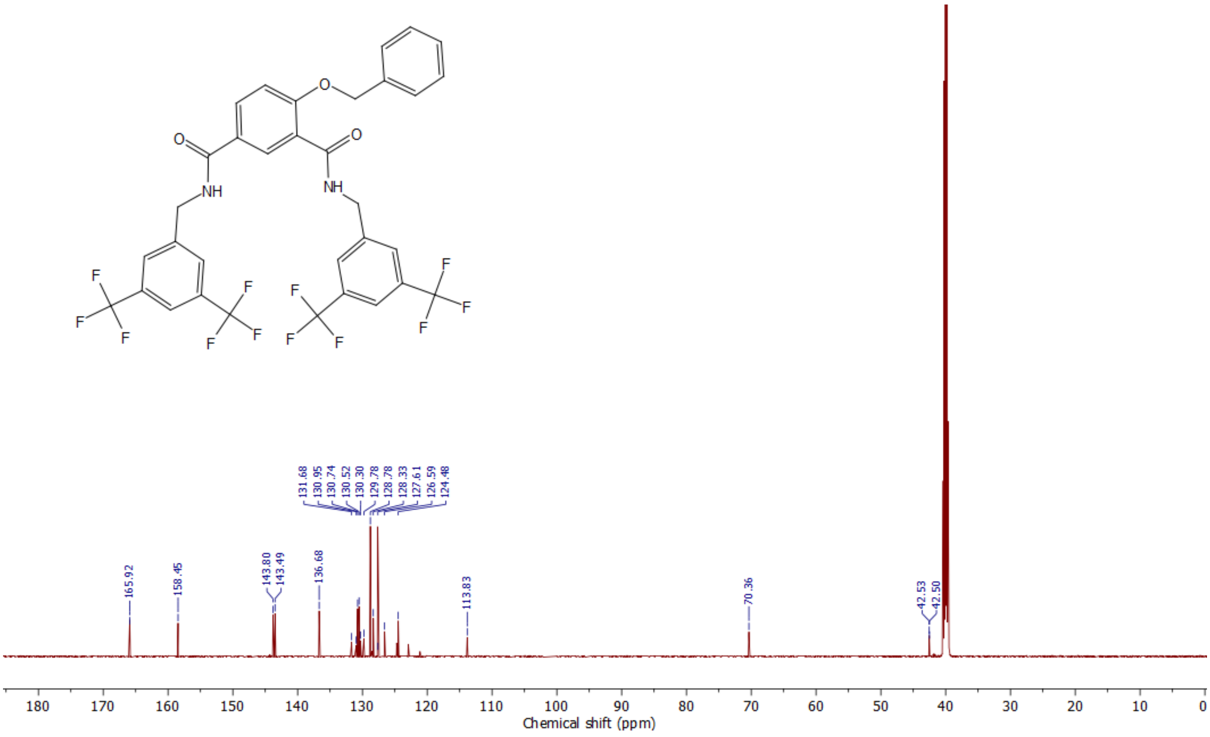

Figure S4: ^{13}C NMR spectrum of **8a** in DMSO-d_6 (151 MHz, 298 K).

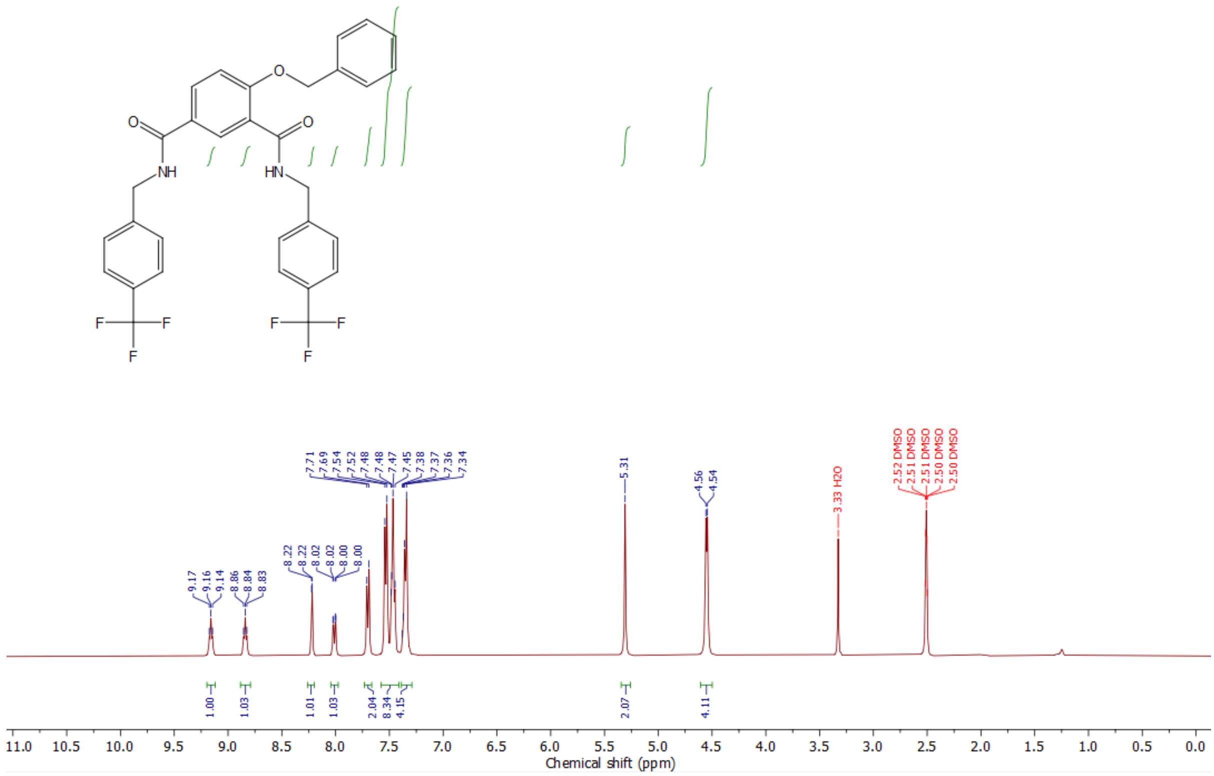

Figure S5: ^{1}H NMR spectrum of **8b** in DMSO-d_6 (600 MHz, 298 K).

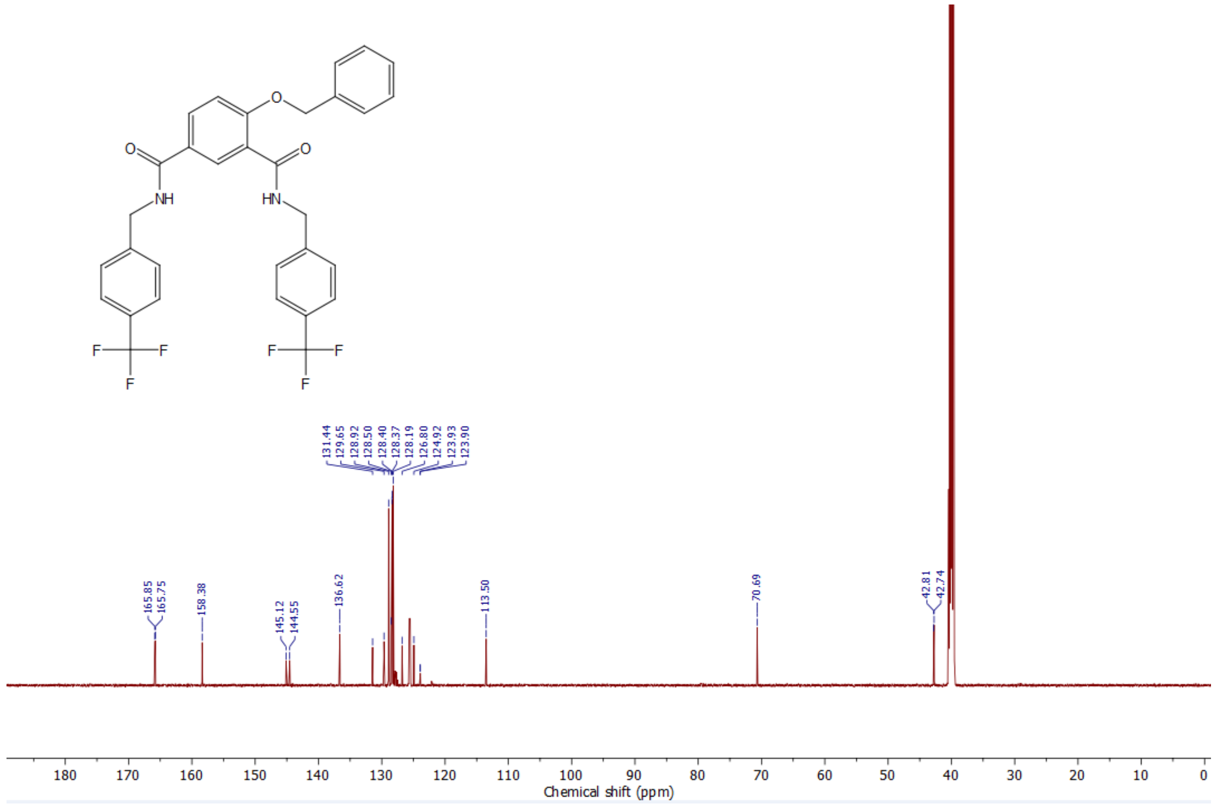

Figure S6: ^{13}C NMR spectrum of **8b** in DMSO-d_6 (151 MHz, 298 K).

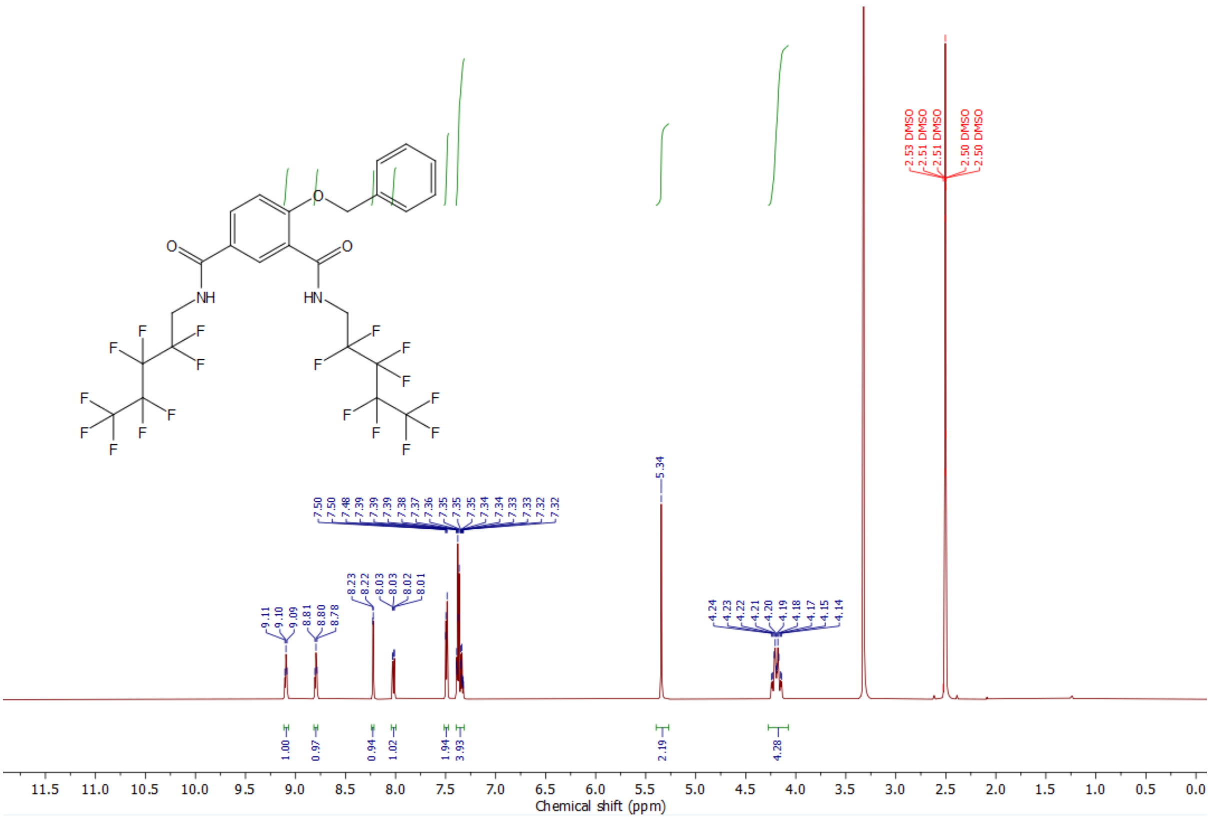

Figure S7: ^{1}H NMR spectrum of **8c** in DMSO-d_6 (600 MHz, 298 K).

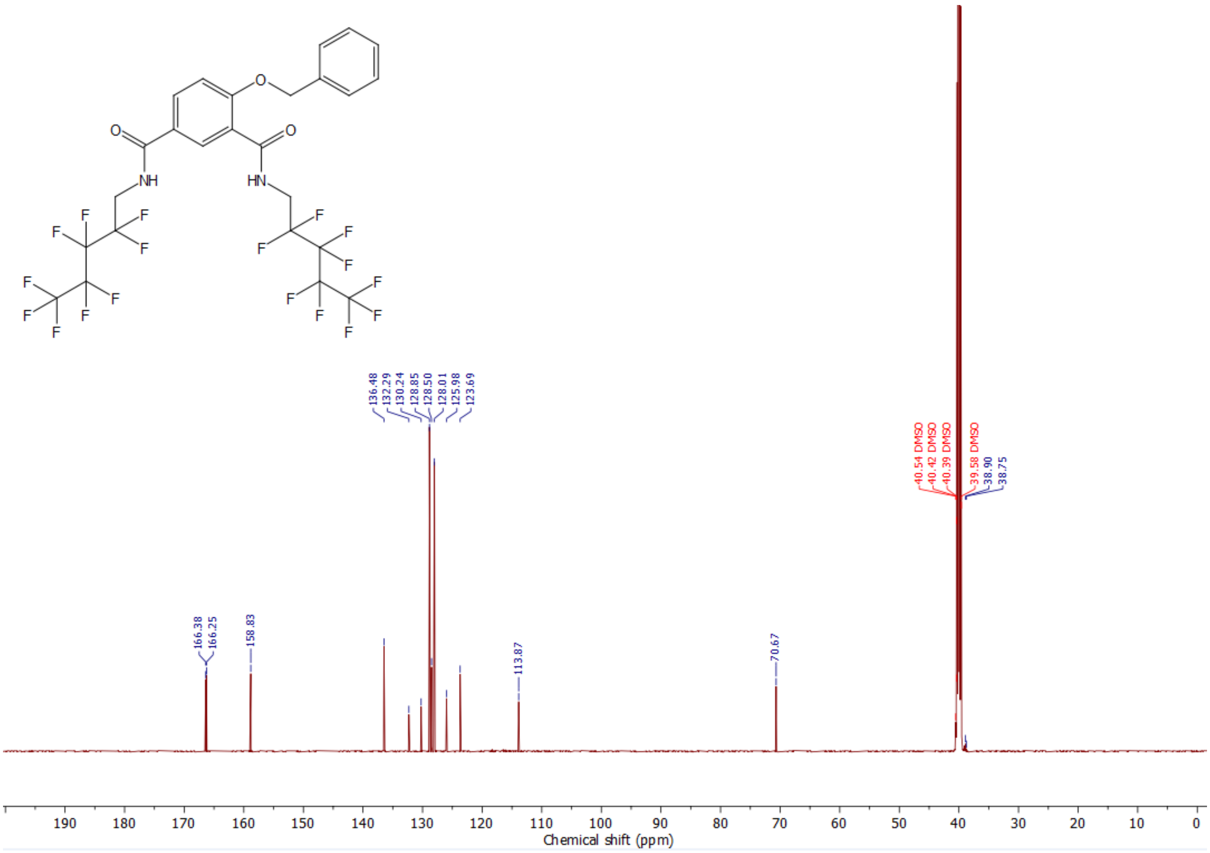

Figure S8: ^{13}C NMR spectrum of **8c** in DMSO-d_6 (151 MHz, 298 K).

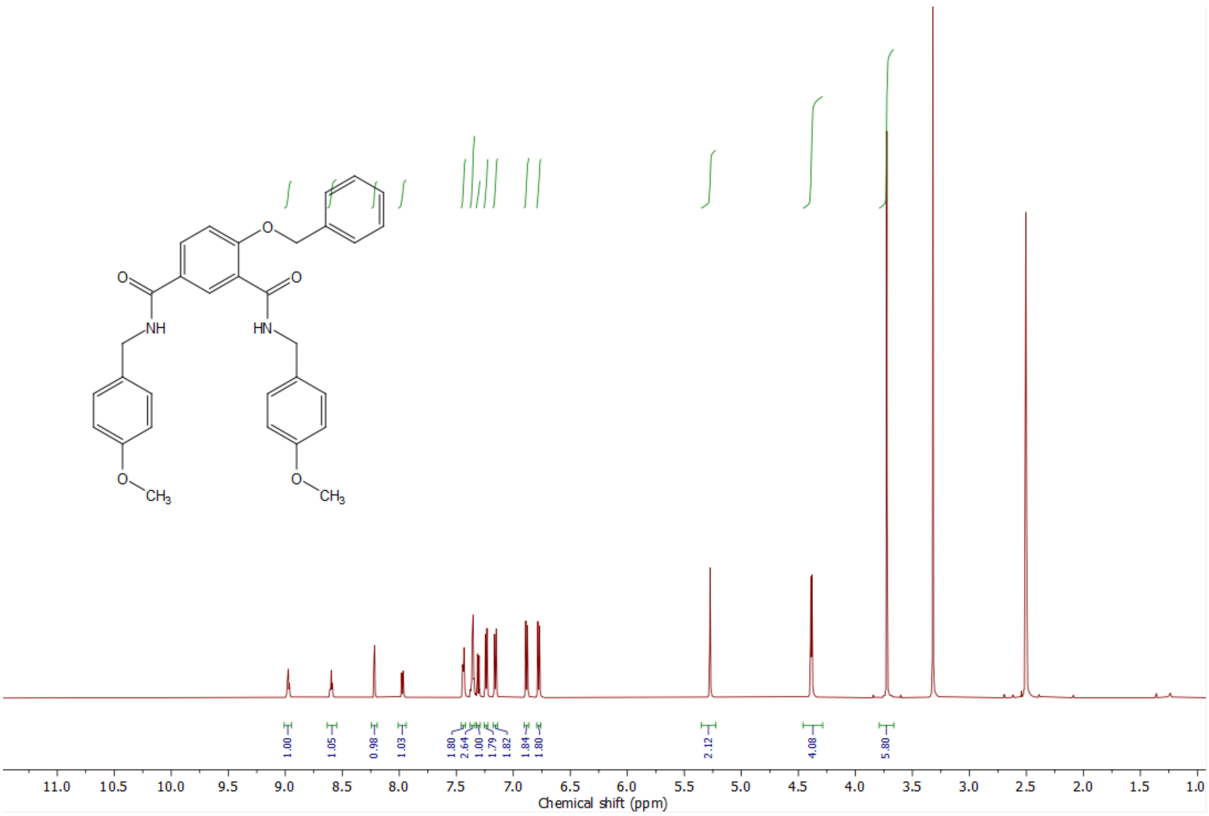

Figure S9: ^{1}H NMR spectrum of **8d** in DMSO-d_6 (600 MHz, 298 K).

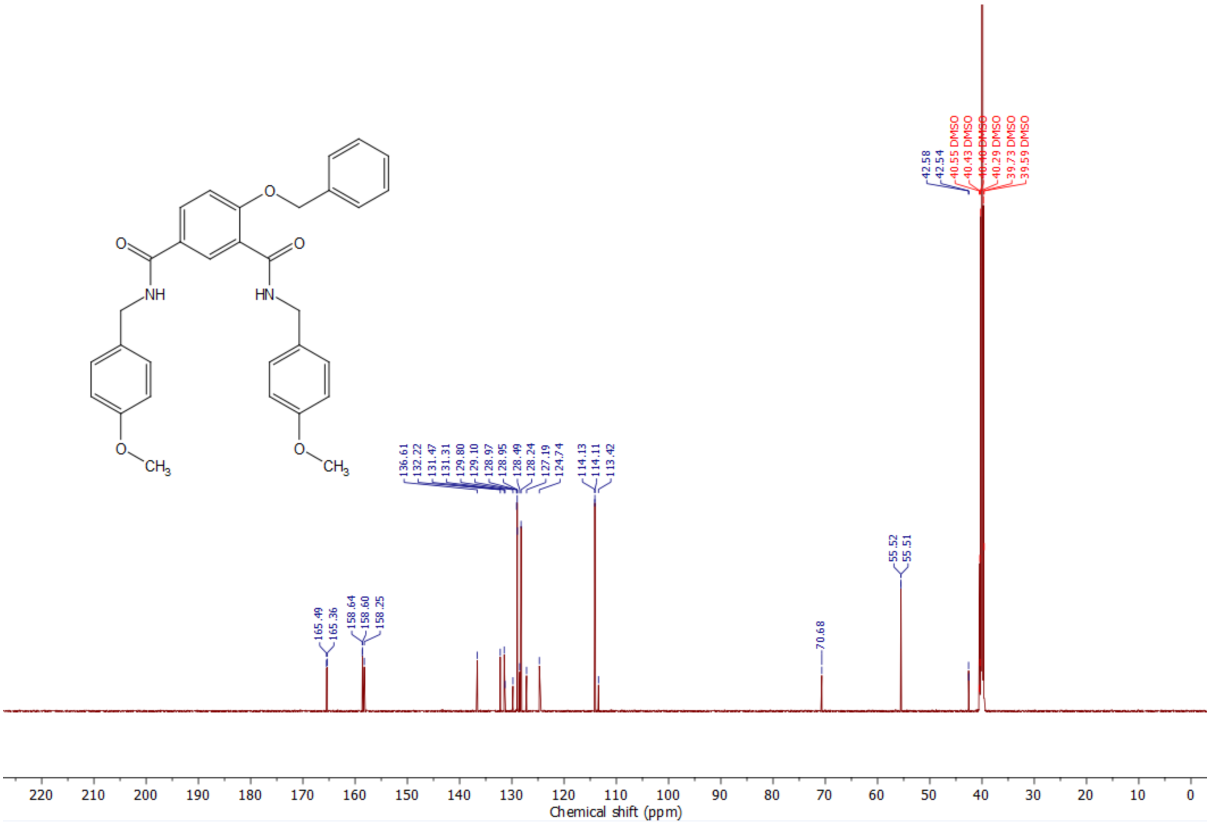

Figure S10: ^{13}C NMR spectrum of **8d** in DMSO-d_6 (151 MHz, 298 K).

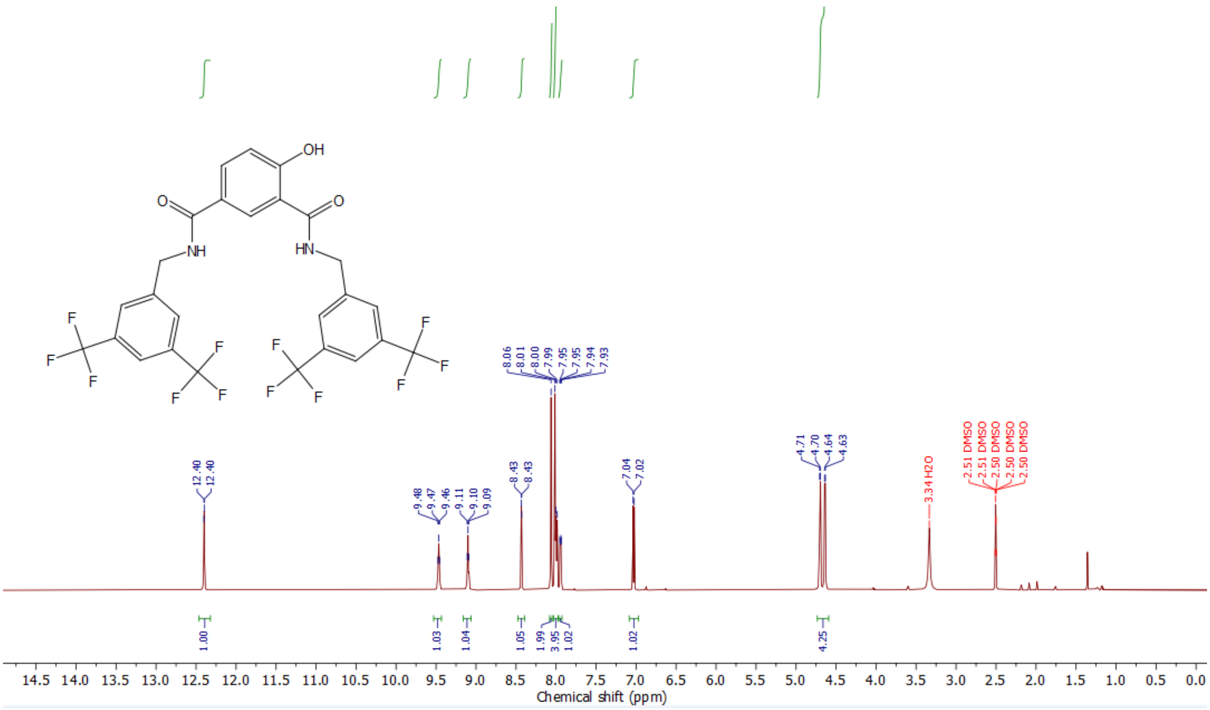

Figure S11: ^{1}H NMR spectrum of **1** in DMSO-d_6 (600 MHz, 298 K).

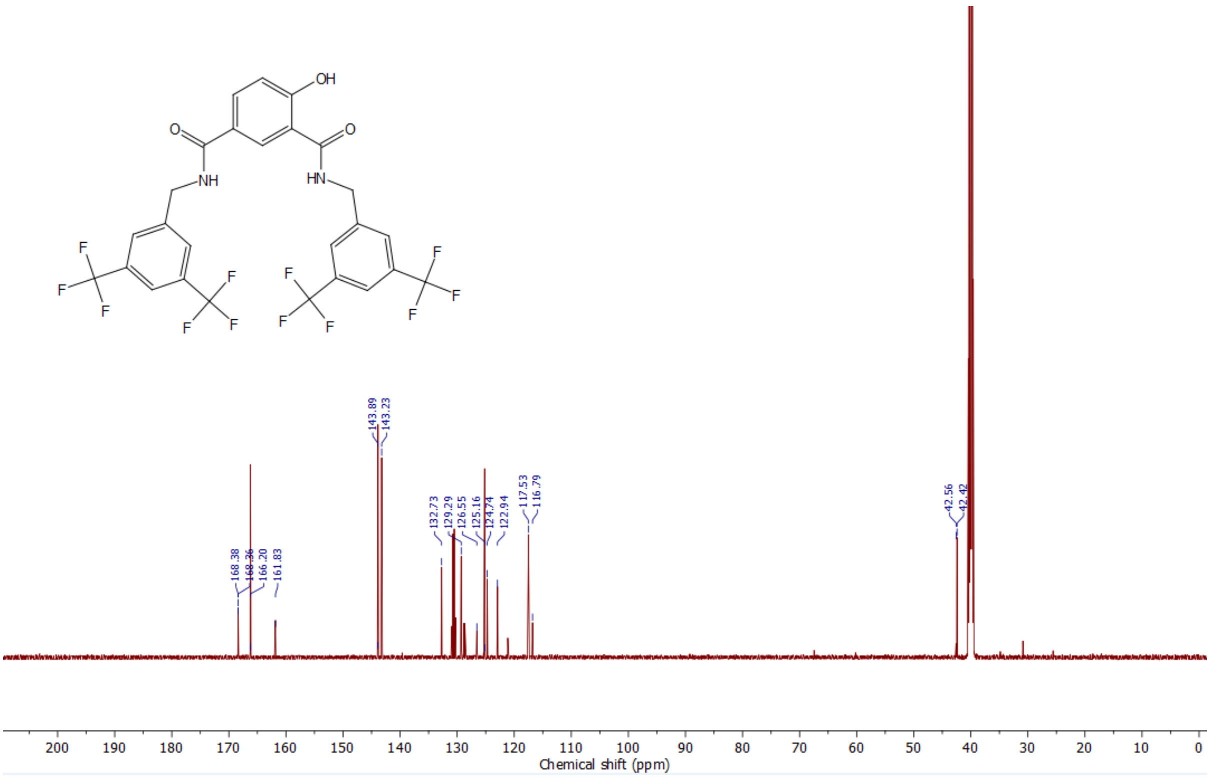

Figure S12: ^{13}C NMR spectrum of **1** in DMSO-d_6 (151 MHz, 298 K).

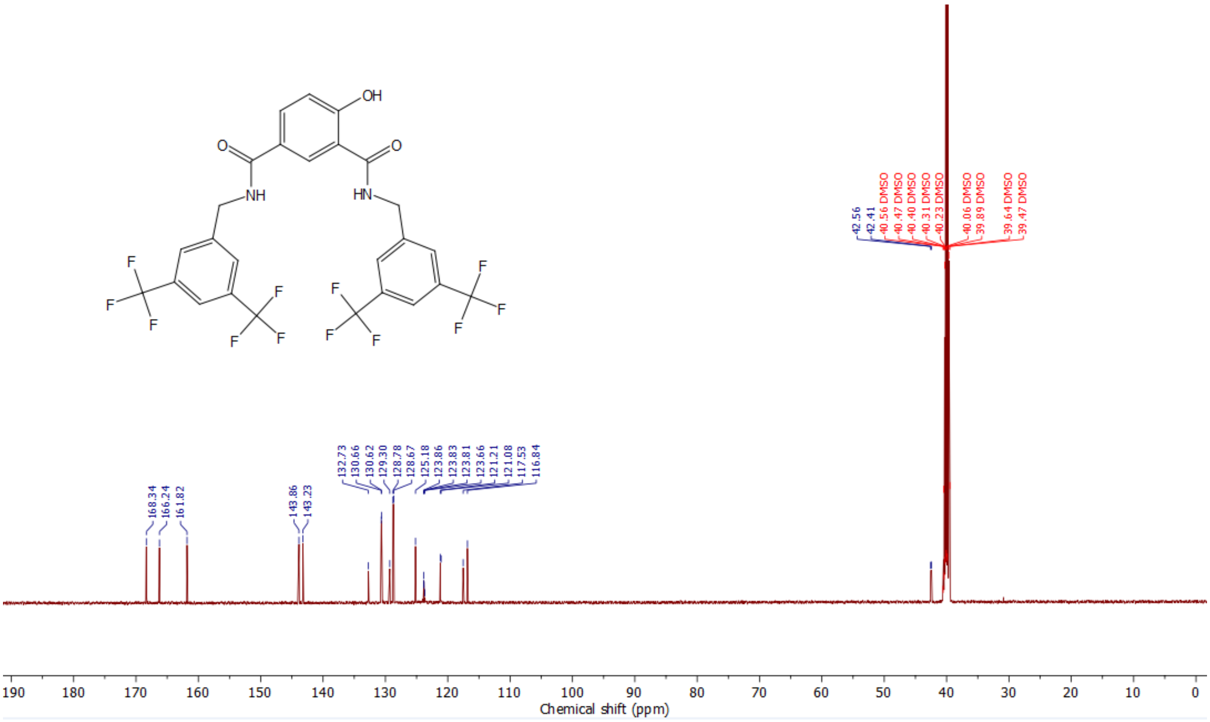

Figure S13: $^{13}C\{^{19}F\}$ NMR spectrum of **1** in DMSO-d_6 (126 MHz, 298 K).

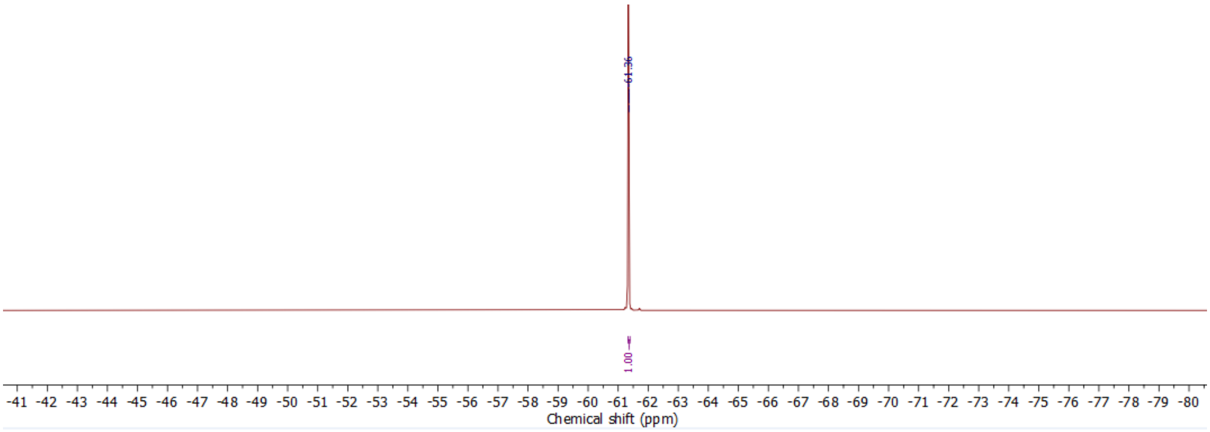

Figure S14: ^{19}F NMR spectrum of **1** in DMSO-d_6 (565 MHz, 298 K).

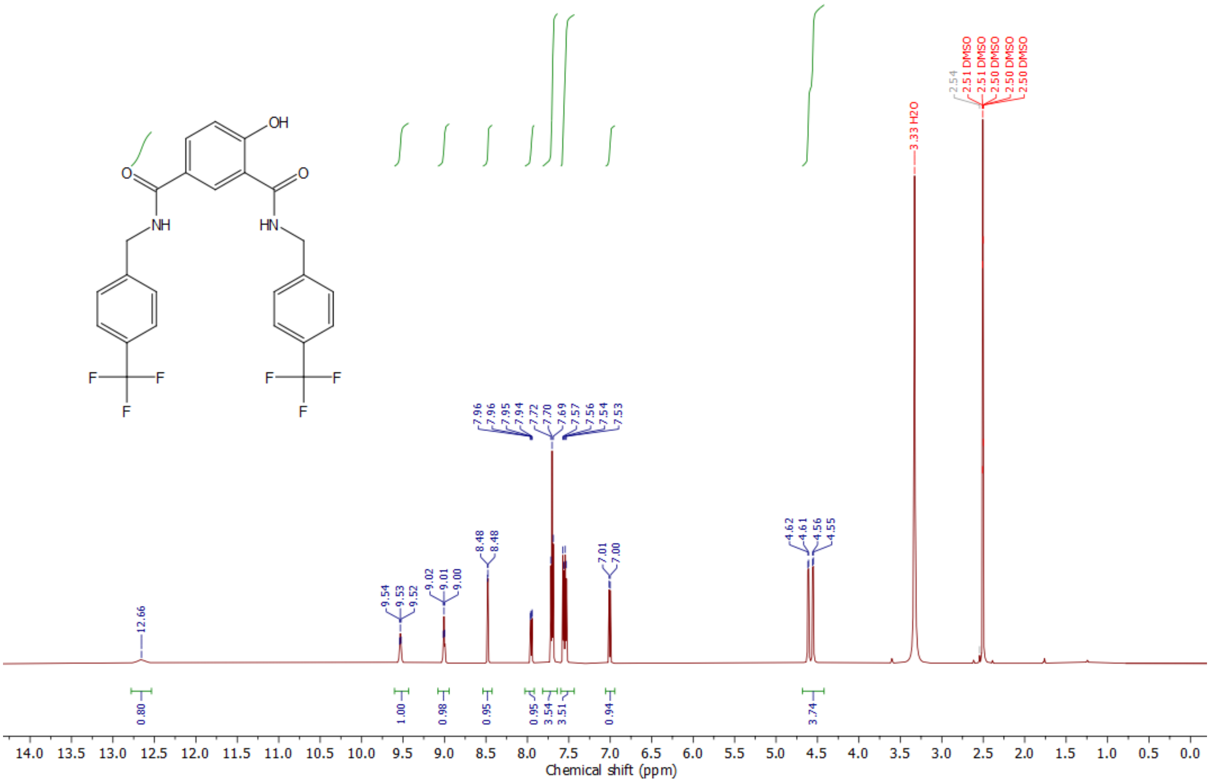

Figure S15: ^{1}H NMR spectrum of **2** in DMSO-d_6 (600 MHz, 298 K).

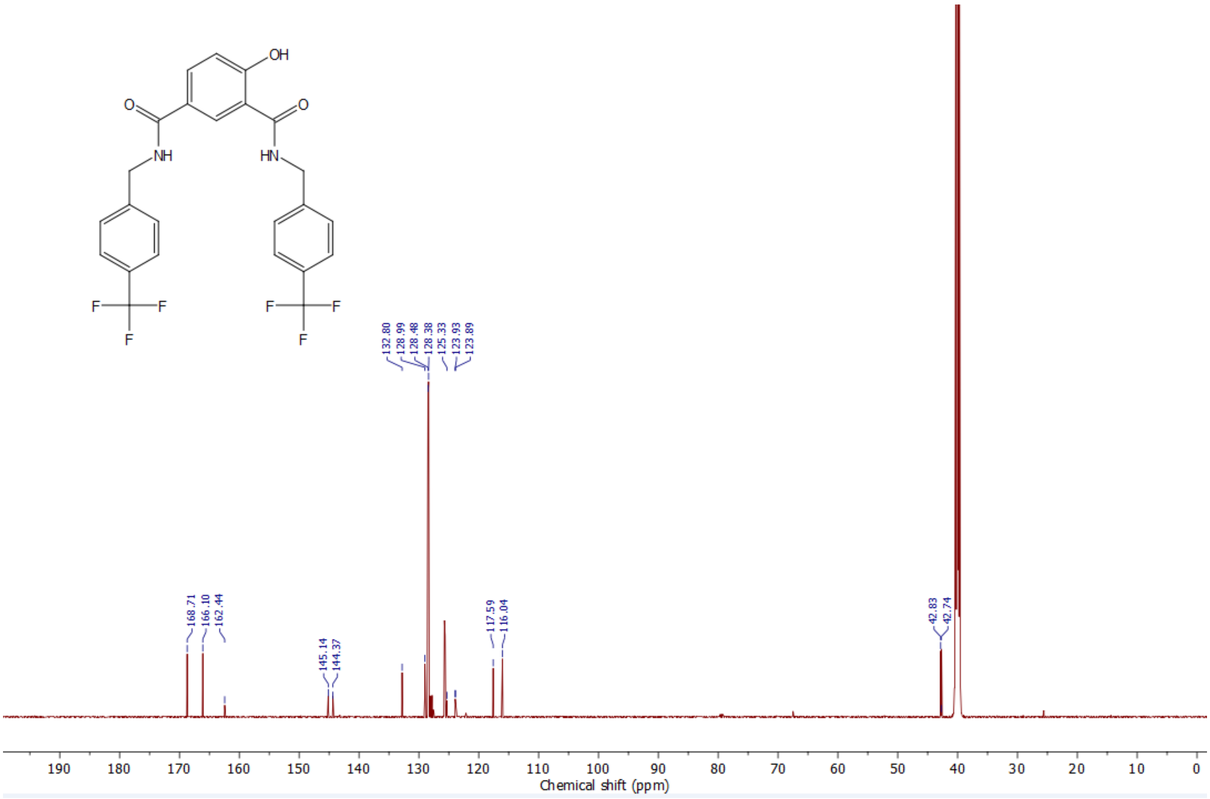

Figure S16: ^{13}C NMR spectrum of **2** in DMSO-d_6 (151 MHz, 298 K).

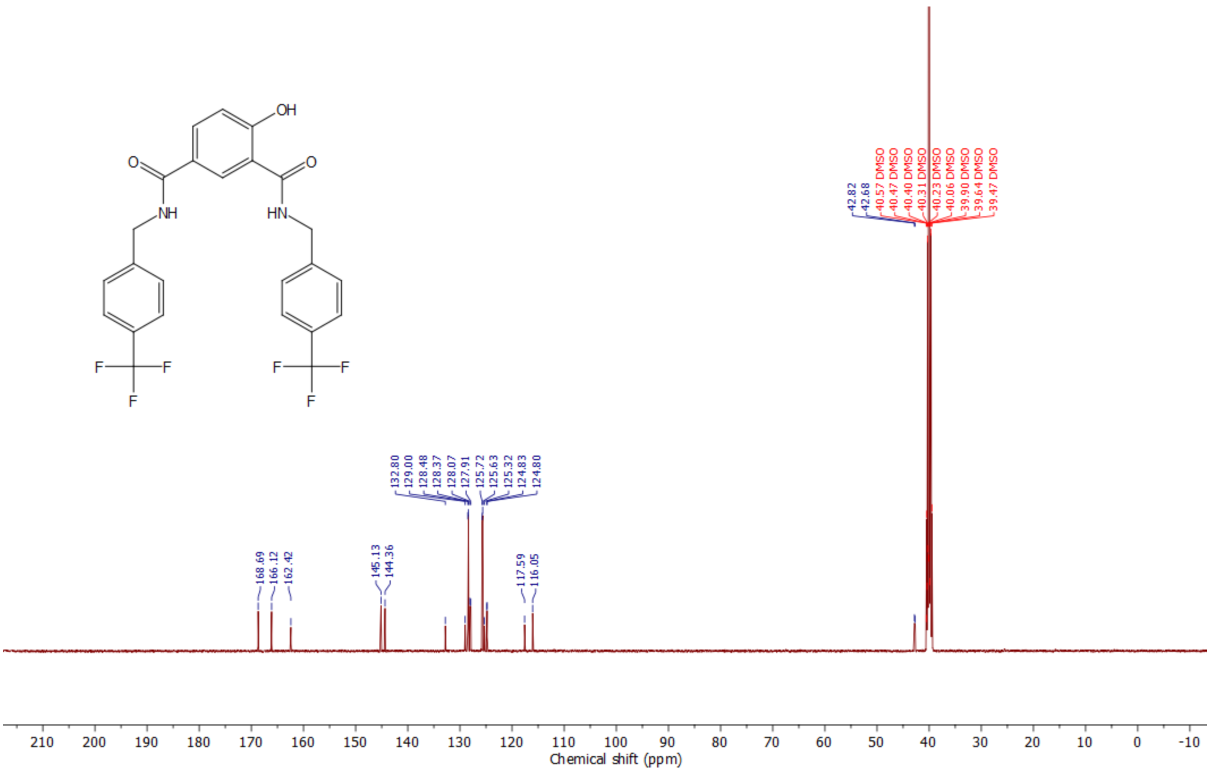

Figure S17: $^{13}C\{^{19}F\}$ NMR spectrum of **2** in DMSO-d_6 (126 MHz, 298 K).

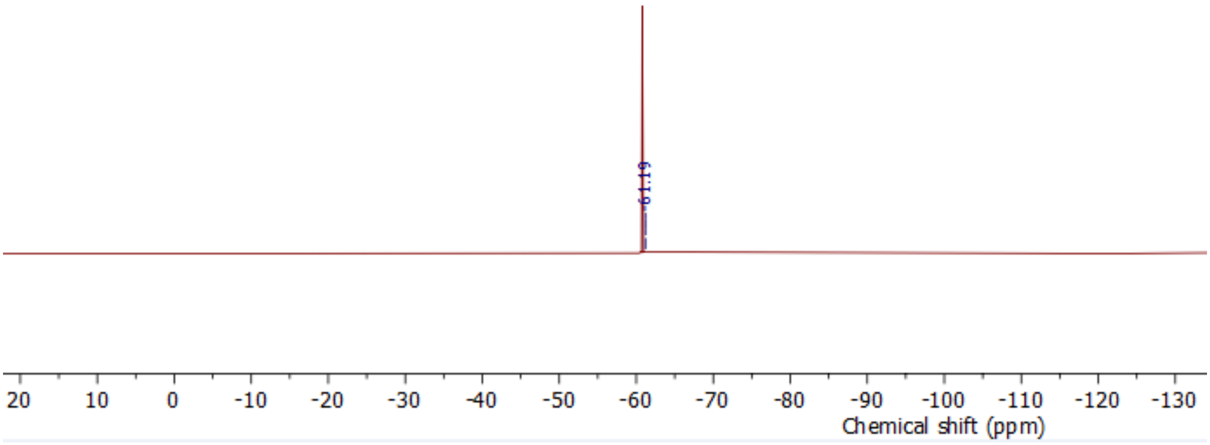

Figure S18: ^{19}F NMR spectrum of **2** in DMSO-d_6 (565 MHz, 298 K).

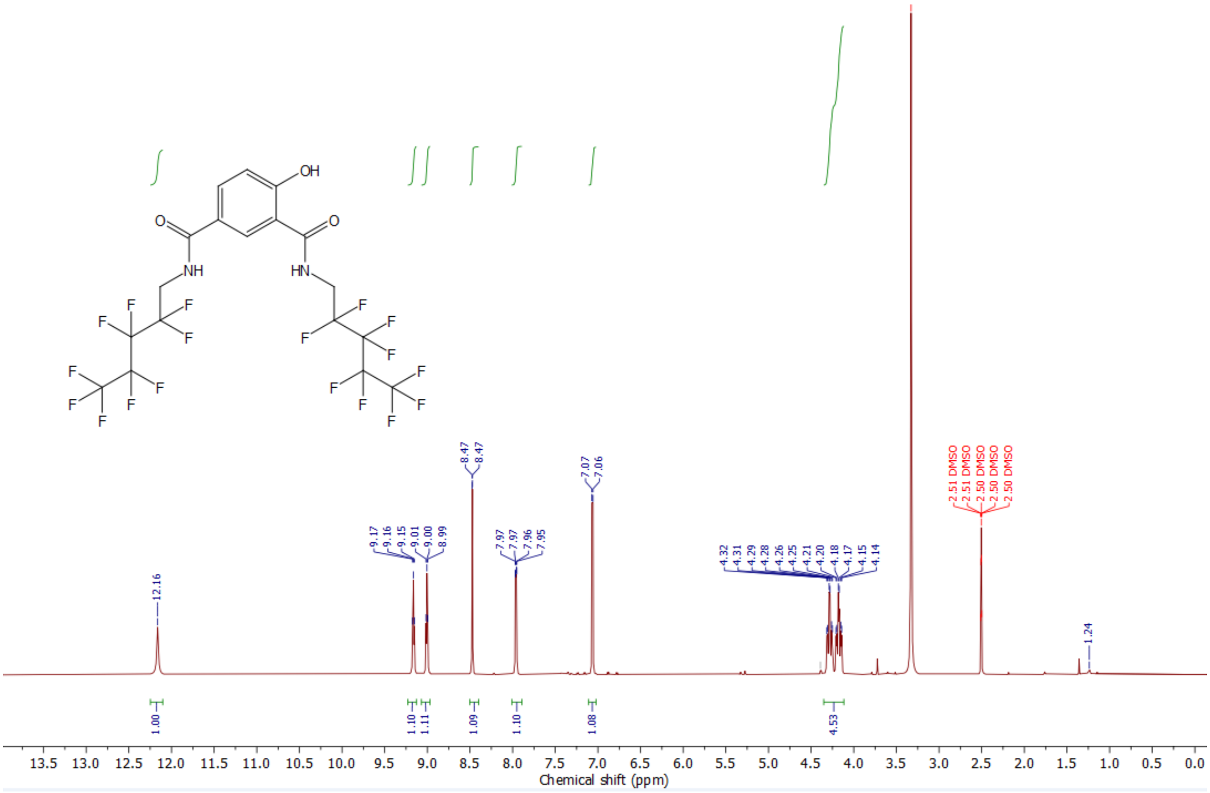

Figure S19: ^{1}H NMR spectrum of **3** in DMSO-d_6 (600 MHz, 298 K).

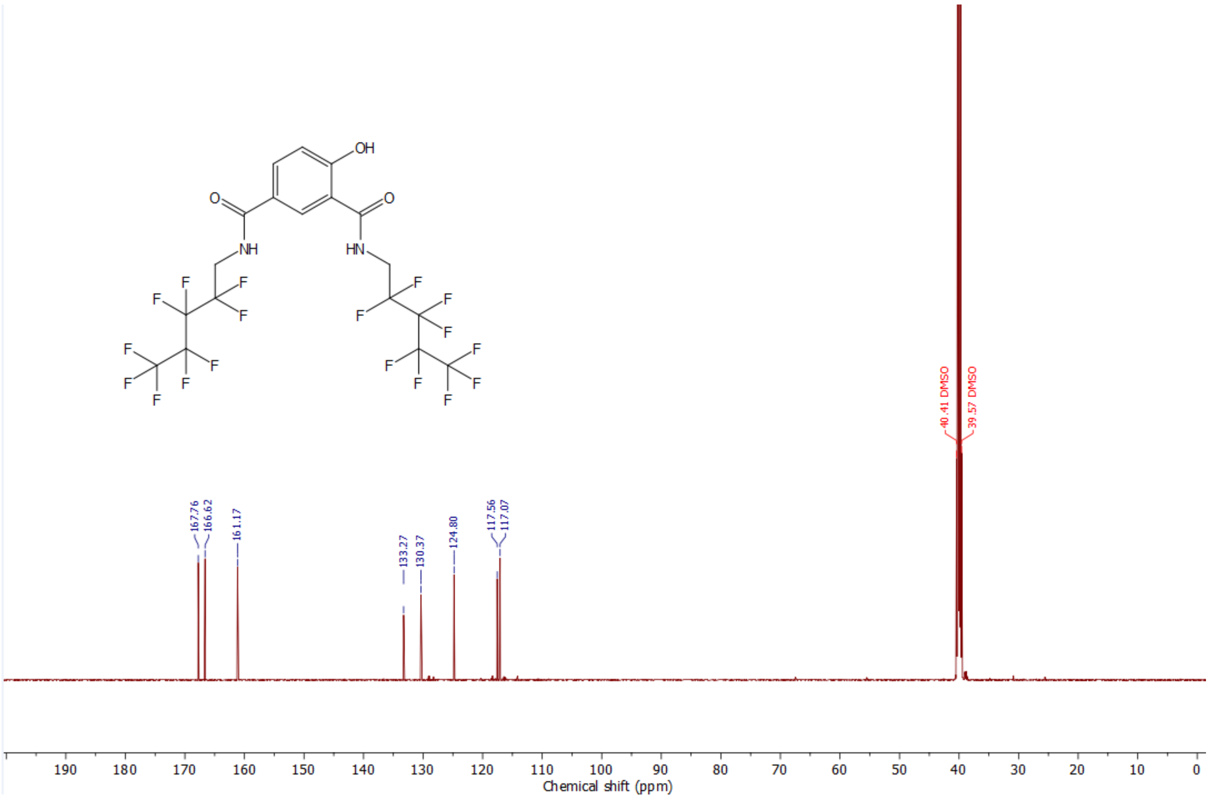

Figure S20: ^{13}C NMR spectrum of **3** in DMSO-d_6 (151 MHz, 298 K).

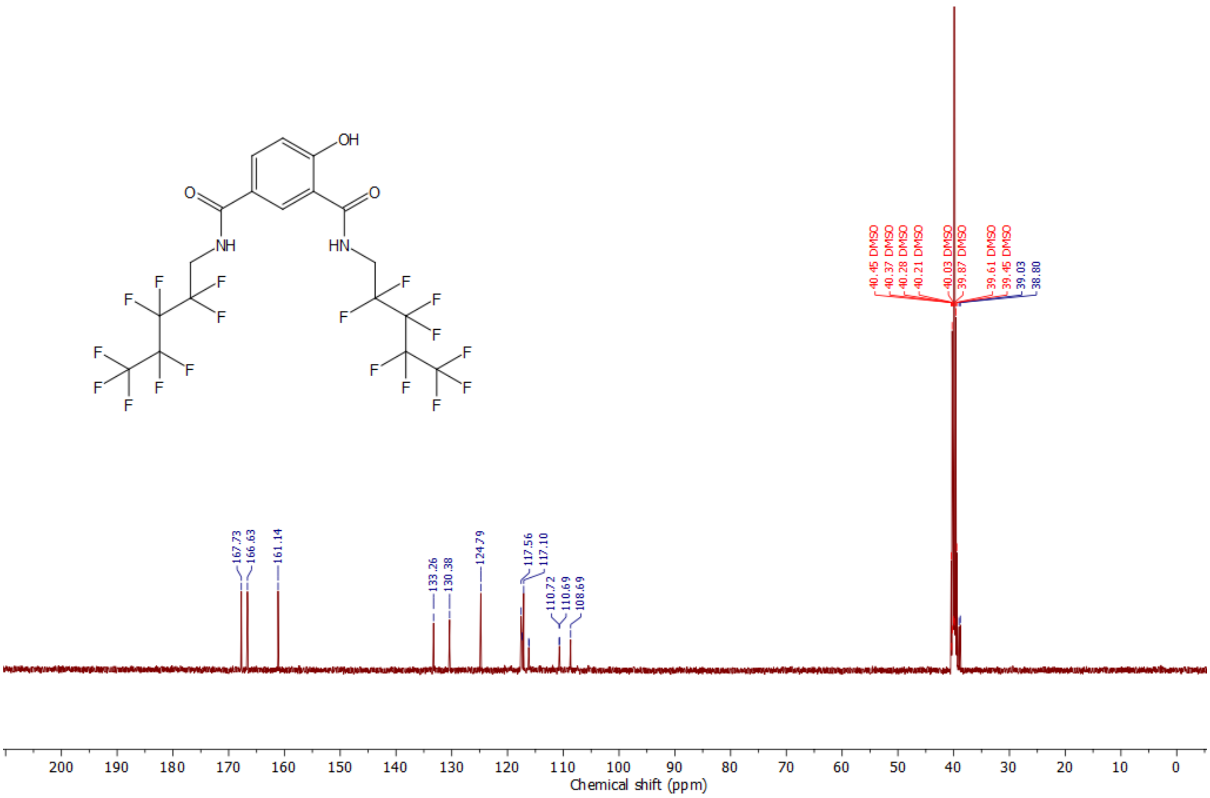

Figure S21: $^{13}C\{^{19}F\}$ NMR spectrum of **3** in DMSO-d_6 (126 MHz, 298 K).

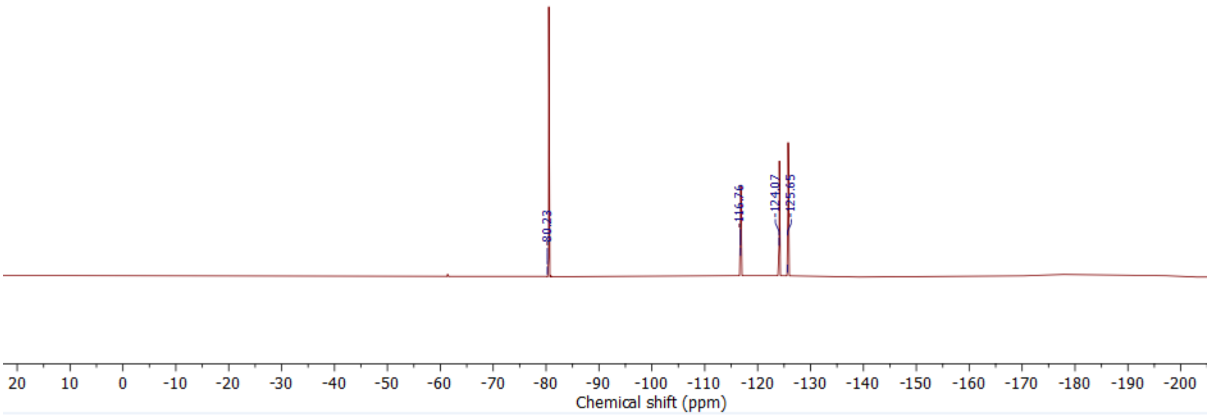

Figure S22: ^{19}F NMR spectrum of **3** in DMSO-d_6 (565 MHz, 298 K).

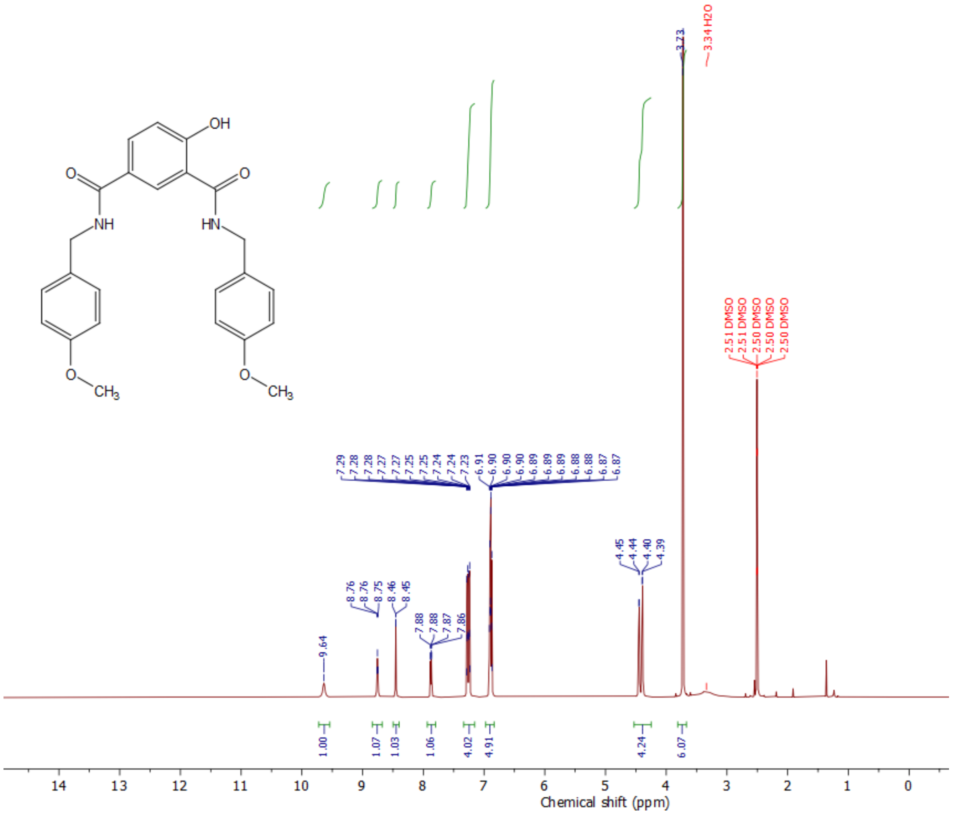

Figure S23: ^{1}H NMR spectrum of **4** in DMSO-d_6 (600 MHz, 298 K).

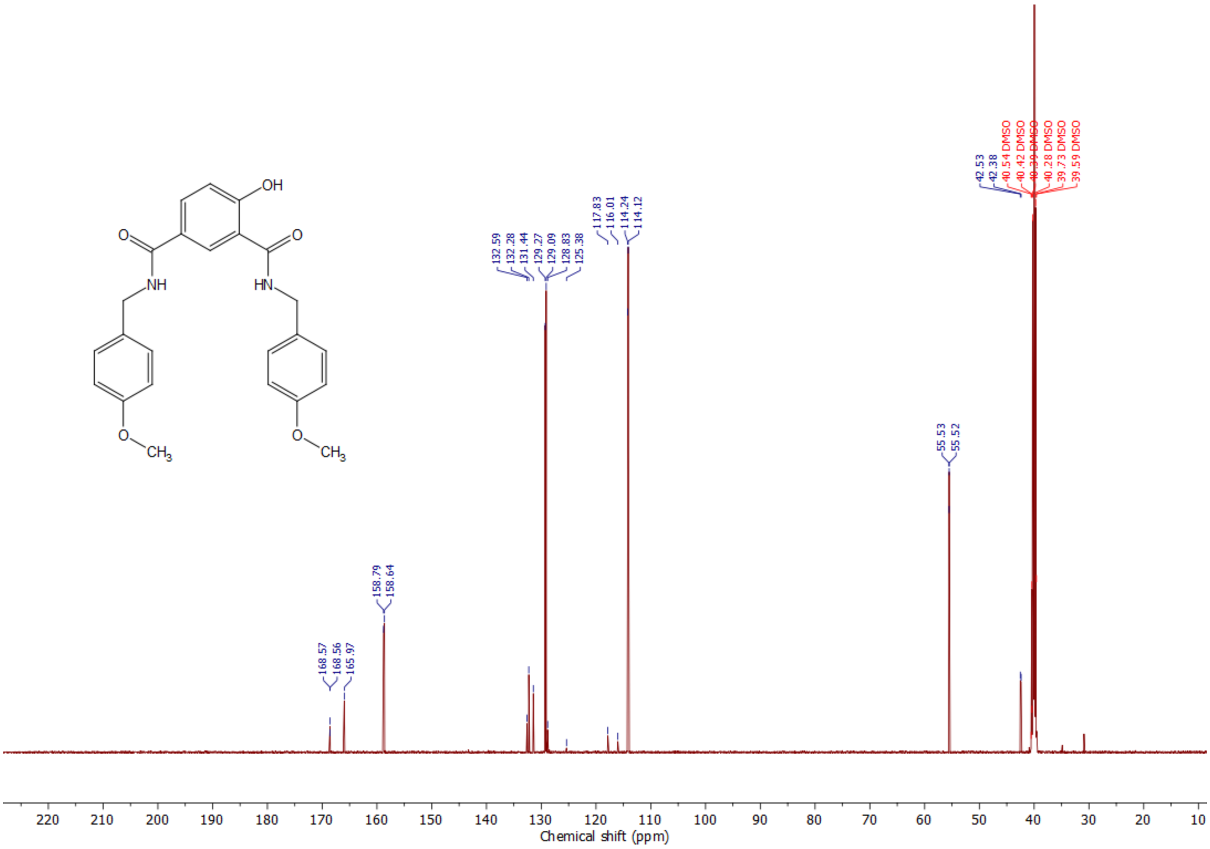

Figure S24: ^{13}C NMR spectrum of **4** in DMSO-d_6 (151 MHz, 298 K).

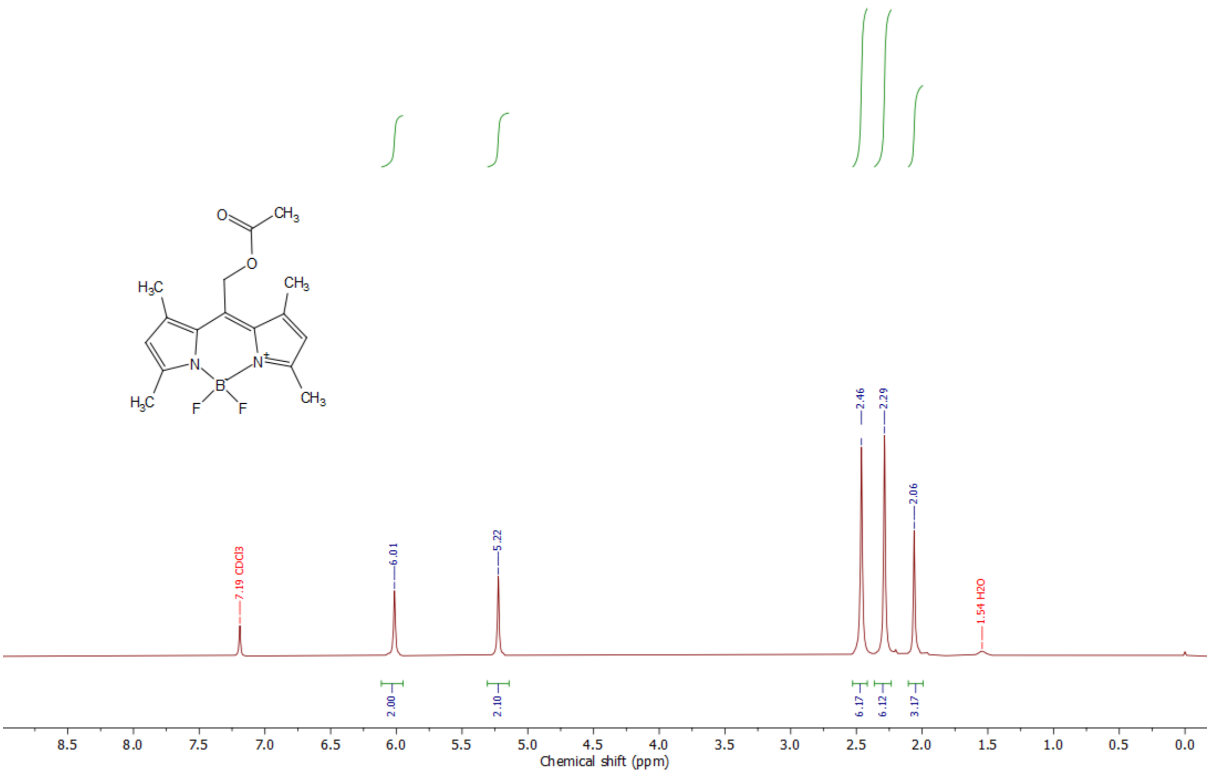

Figure S25: ^{1}H NMR spectrum of **11** in $CDCl_3$ (600 MHz, 298 K).

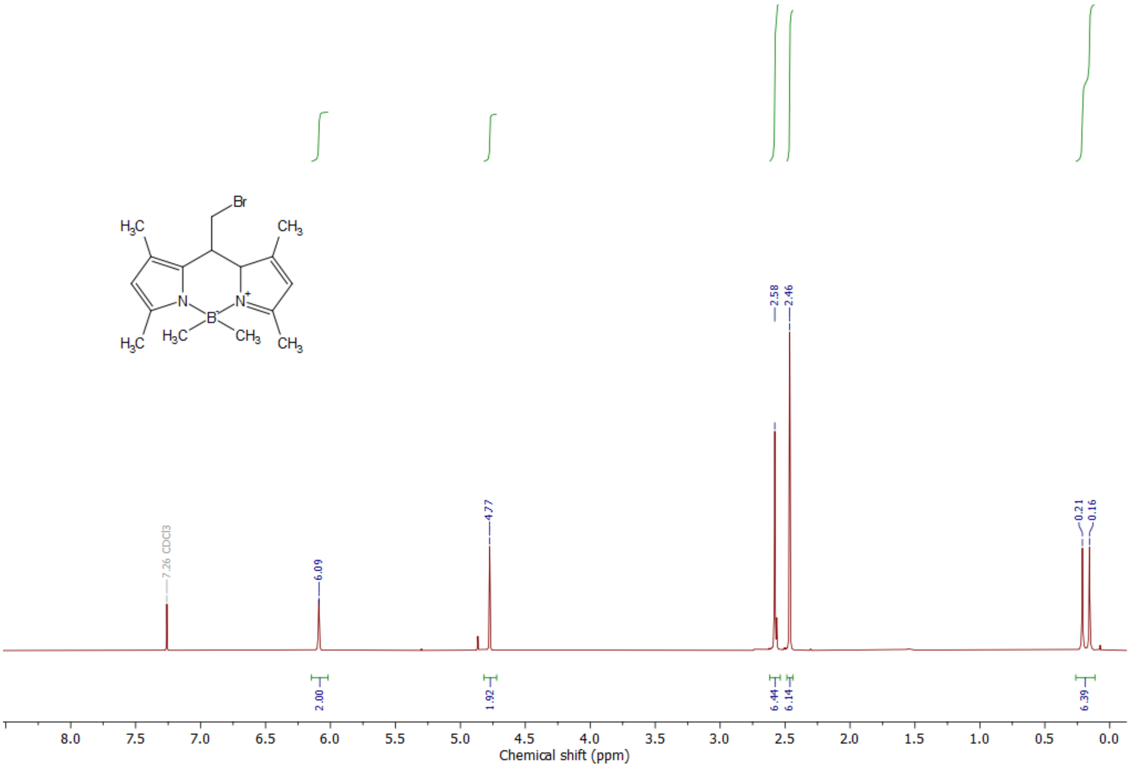

Figure S26: ^{1}H NMR spectrum of **13** in $CDCl_3$ (600 MHz, 298 K).

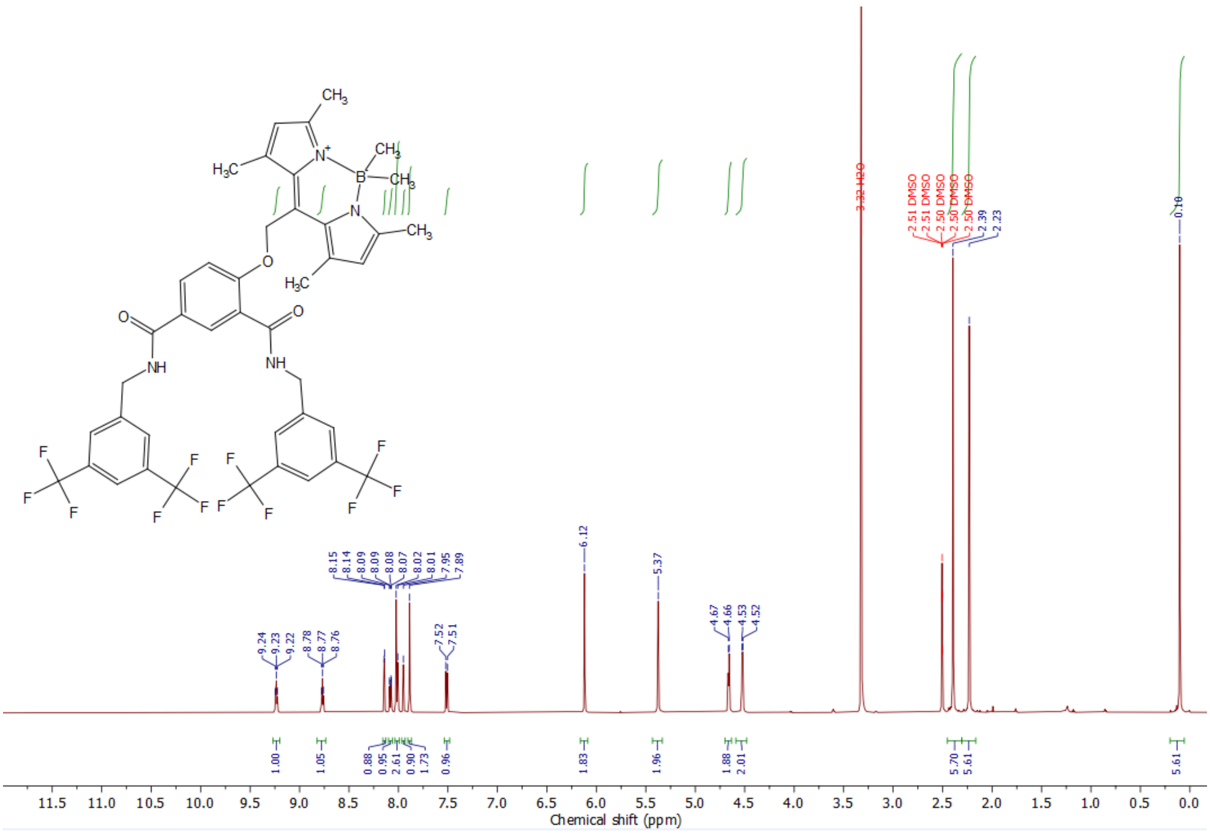

Figure S27: ^{1}H NMR spectrum of **1a** in DMSO-d_6 (600 MHz, 298 K).

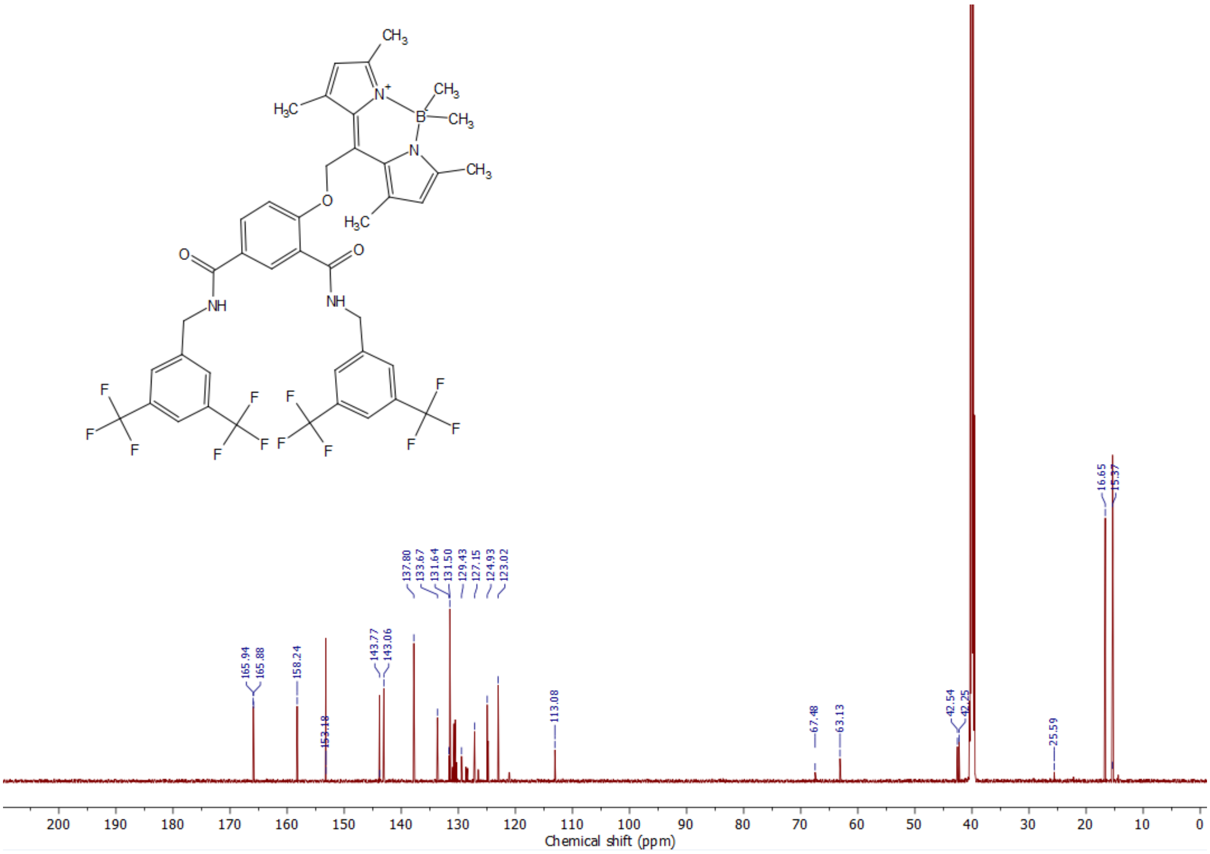

Figure S28: ^{13}C NMR spectrum of **1a** in DMSO-d_6 (151 MHz, 298 K).

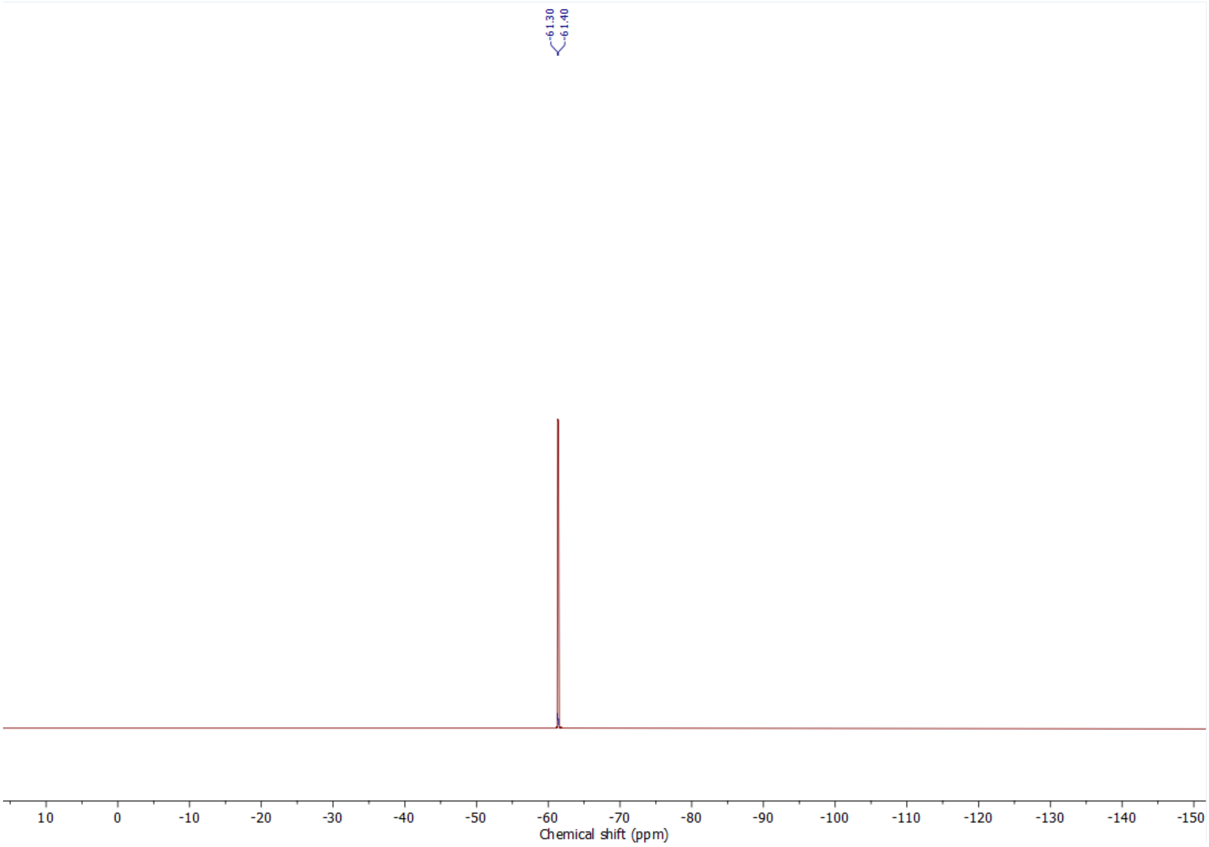

Figure S29: ^{19}F NMR spectrum of 1**a** in DMSO-d_6 (565 MHz, 298 K).

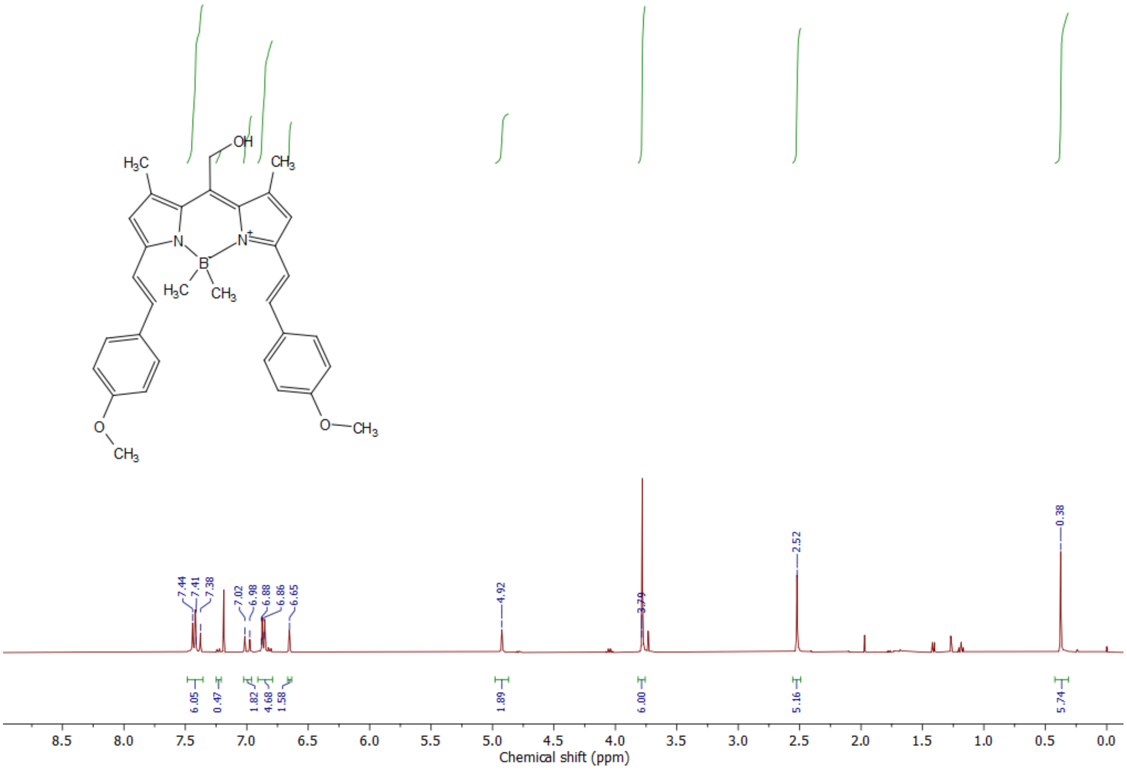

Figure S30: ^{1}H NMR spectrum of **15** in $CDCl_3$ (600 MHz, 298 K).

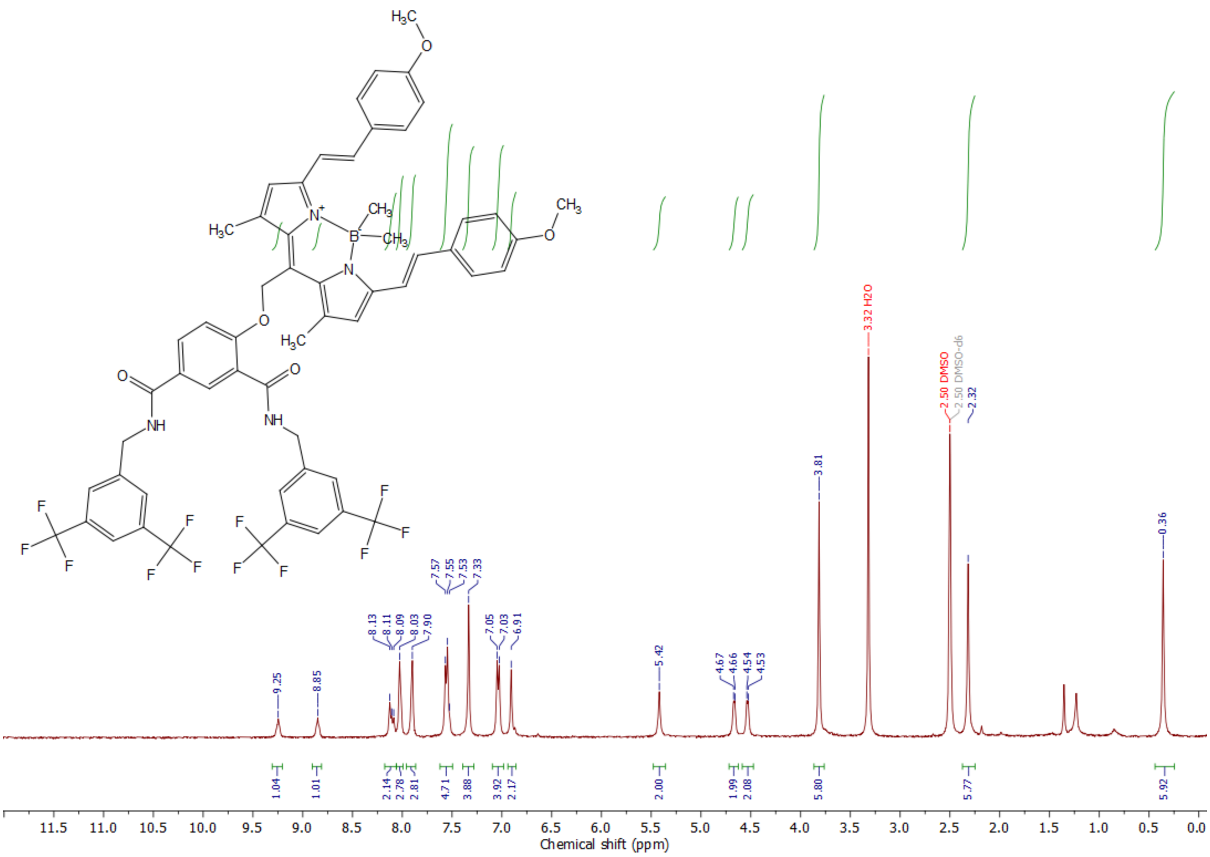

Figure S31: ^{1}H NMR spectrum of **1b** in $CDCl_3$ (600 MHz, 298 K).

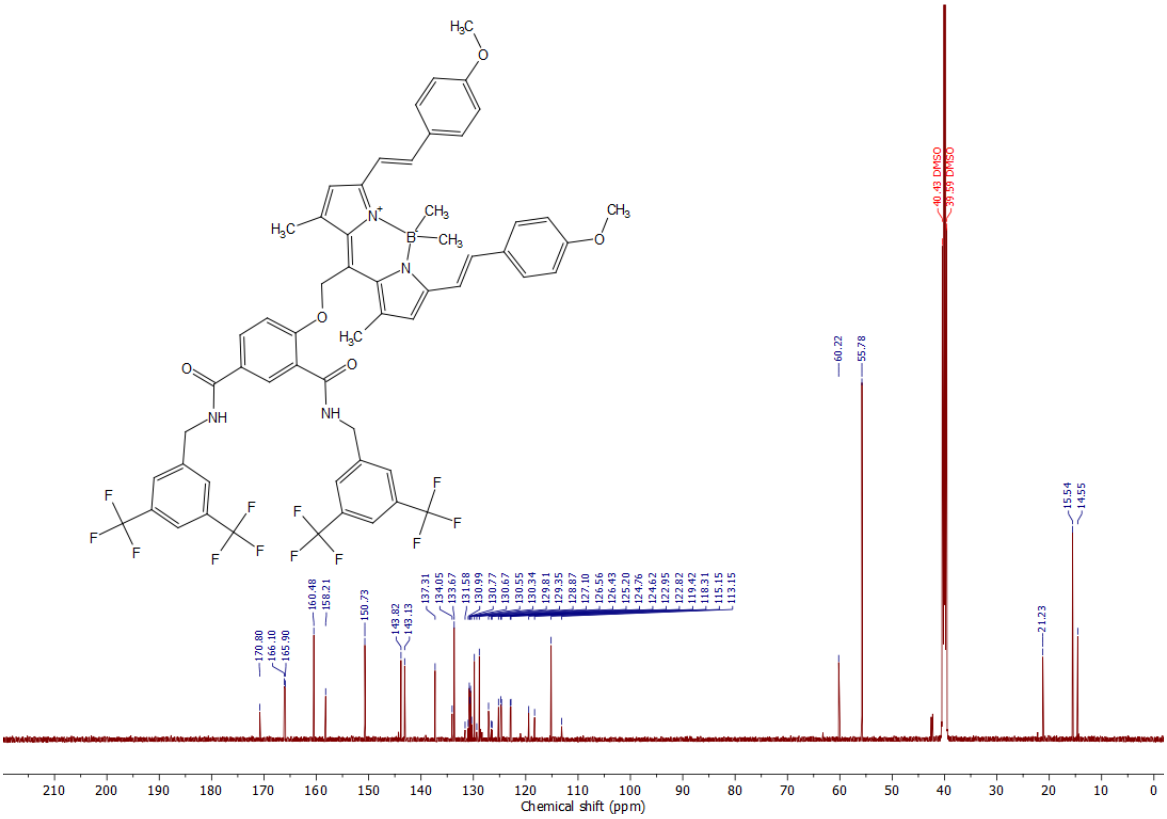

Figure S32: ^{13}C NMR spectrum of **1b** in DMSO-d_6 (151 MHz, 298 K).

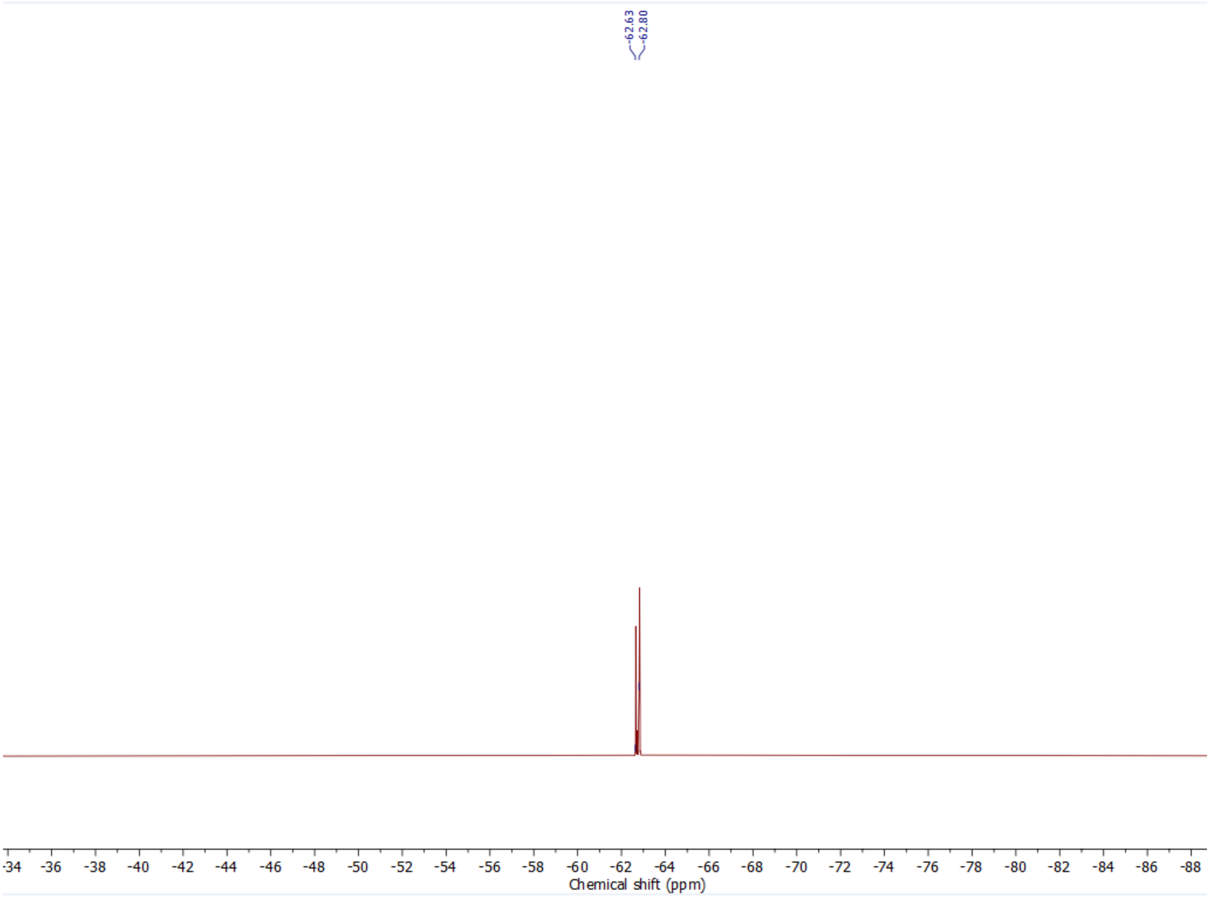

Figure S33: ^{19}F NMR spectrum of **1b** in DMSO-d_6 (565 MHz, 298 K).

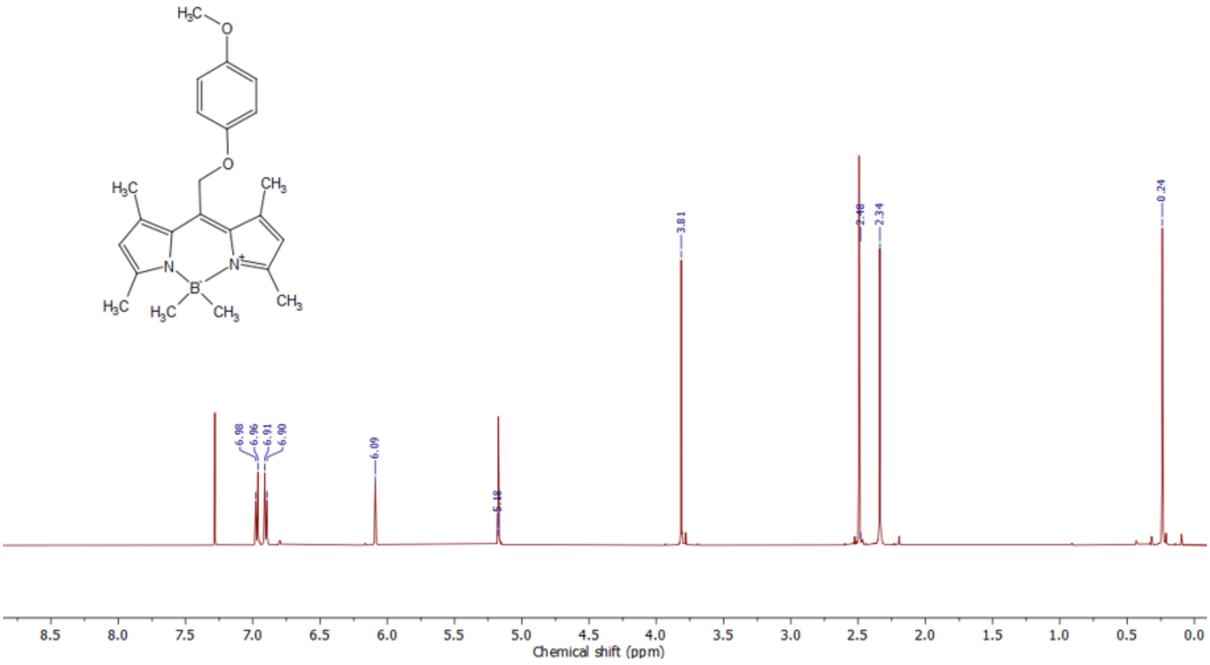

Figure S34: ^{1}H NMR spectrum of C**1** in $CDCl_3$ (600 MHz, 298 K).

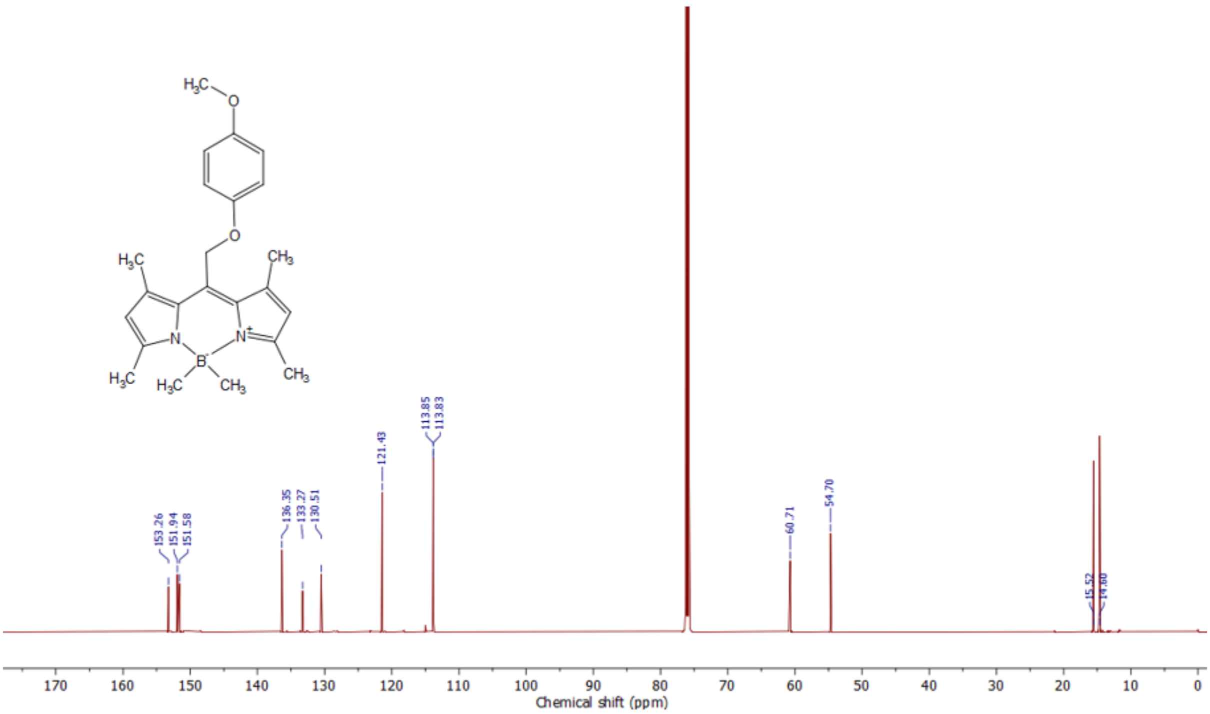

Figure S35: ^{13}C NMR spectrum of **C1** in DMSO-d_6 (151 MHz, 298 K).

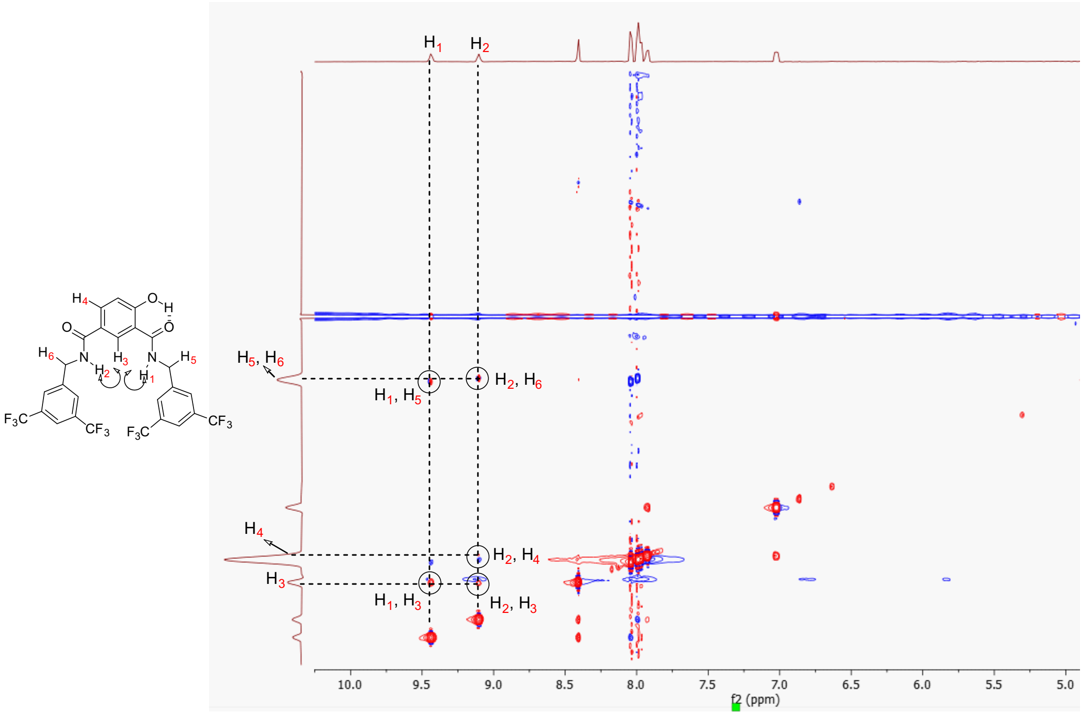

Figure S36. ^{1}H-^{1}H NOESY NMR of **1** in DMSO-d_6

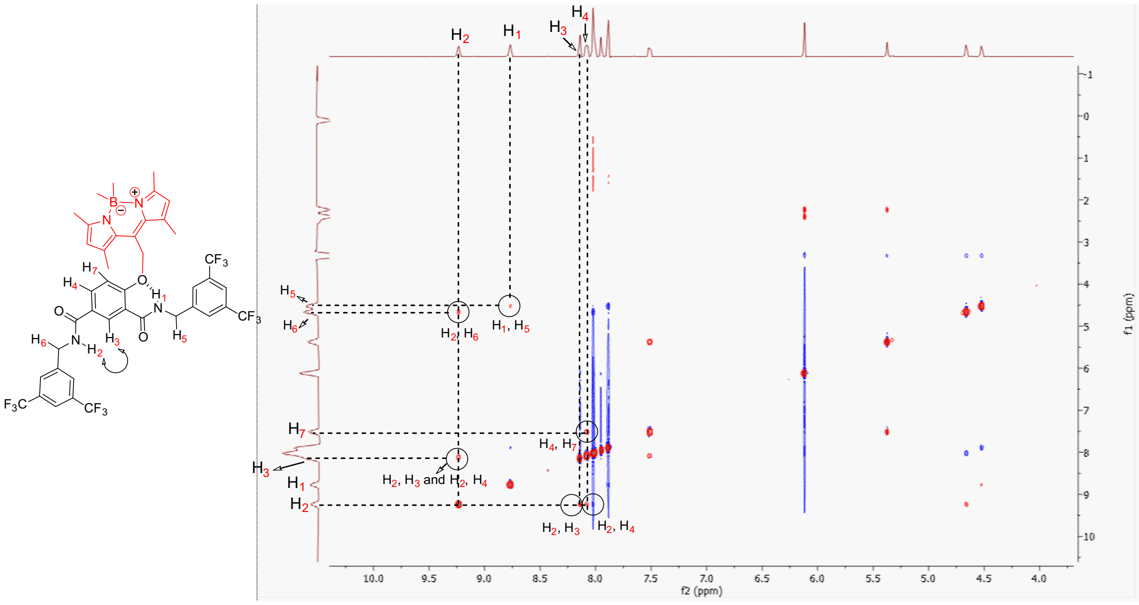

Figure S37. ^{1}H-^{1}H NOESY NMR of **1a** in DMSO-d_6

IV. HRMS Spectra:

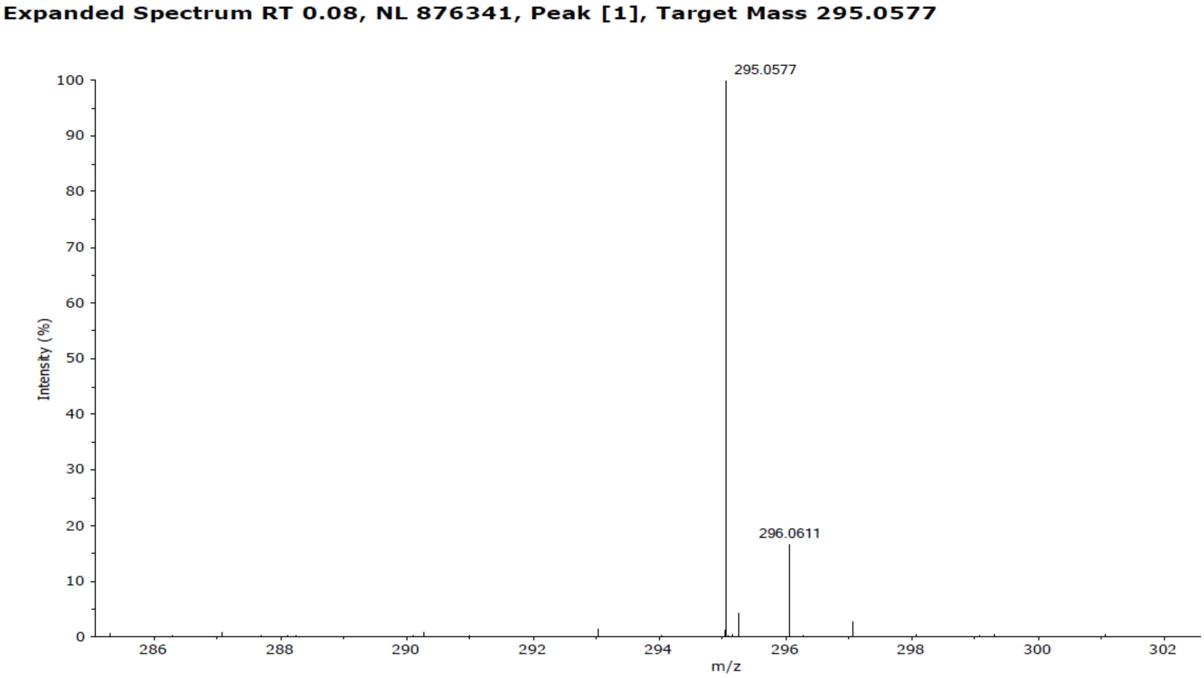

Figure S38: HRMS spectrum of **6.**

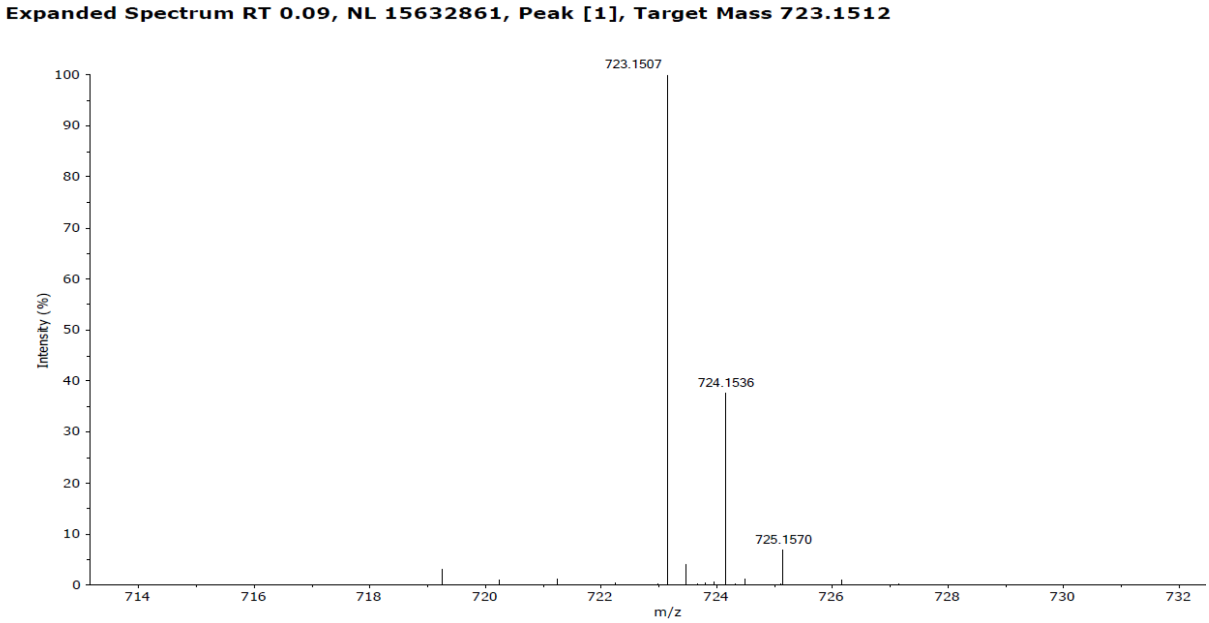

Figure S39: HRMS spectrum of **8a.**

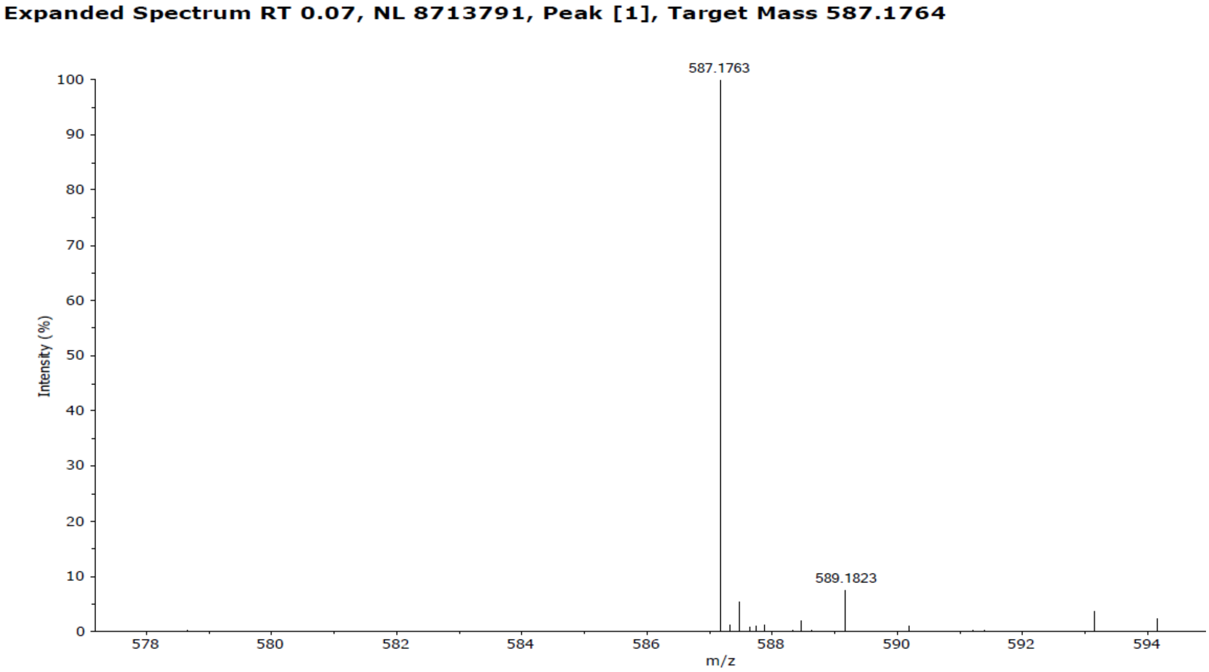

Figure S40: HRMS spectrum of **8b.**

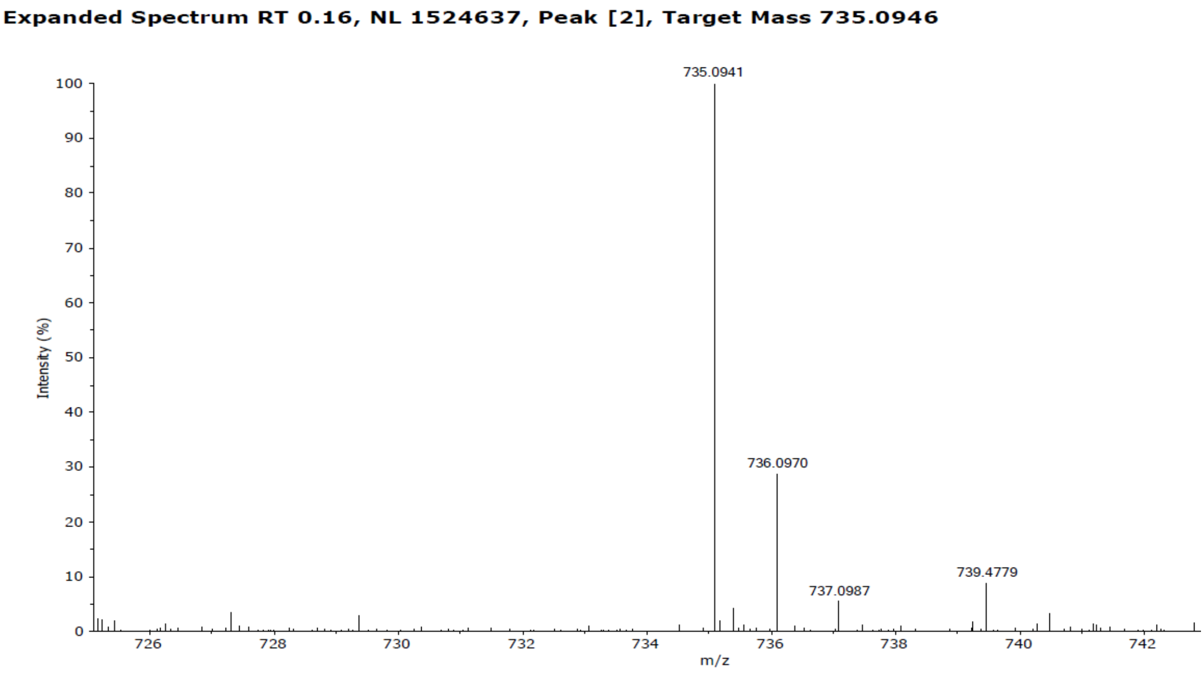

Figure S41: HRMS spectrum of **8c.**

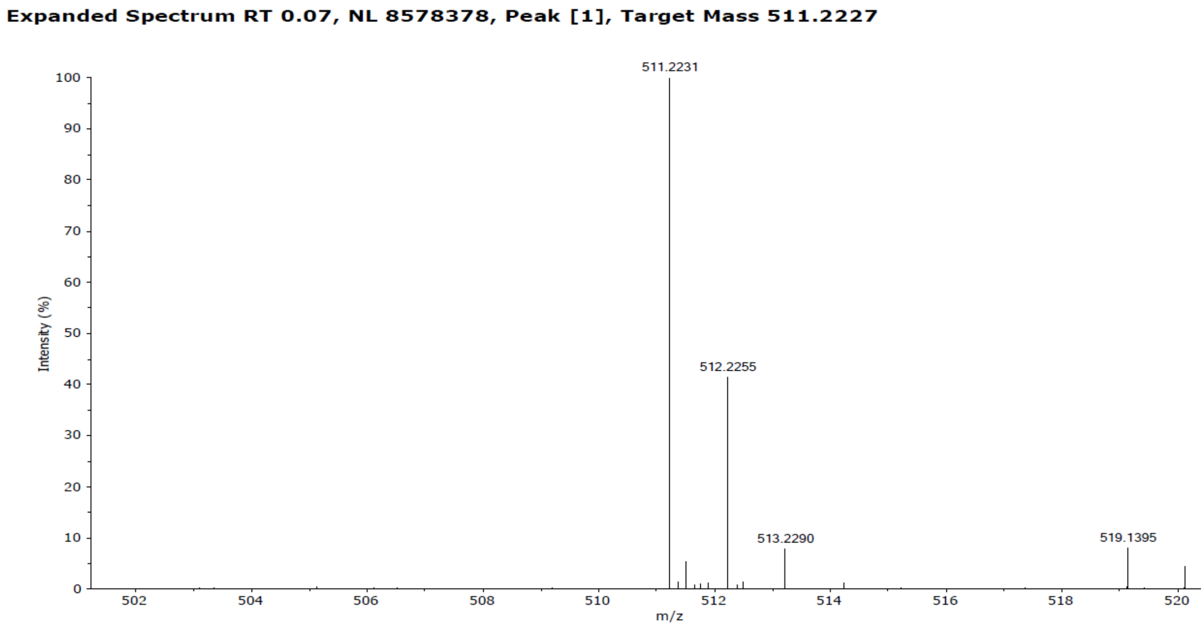

Figure S42: HRMS spectrum of **8d.**

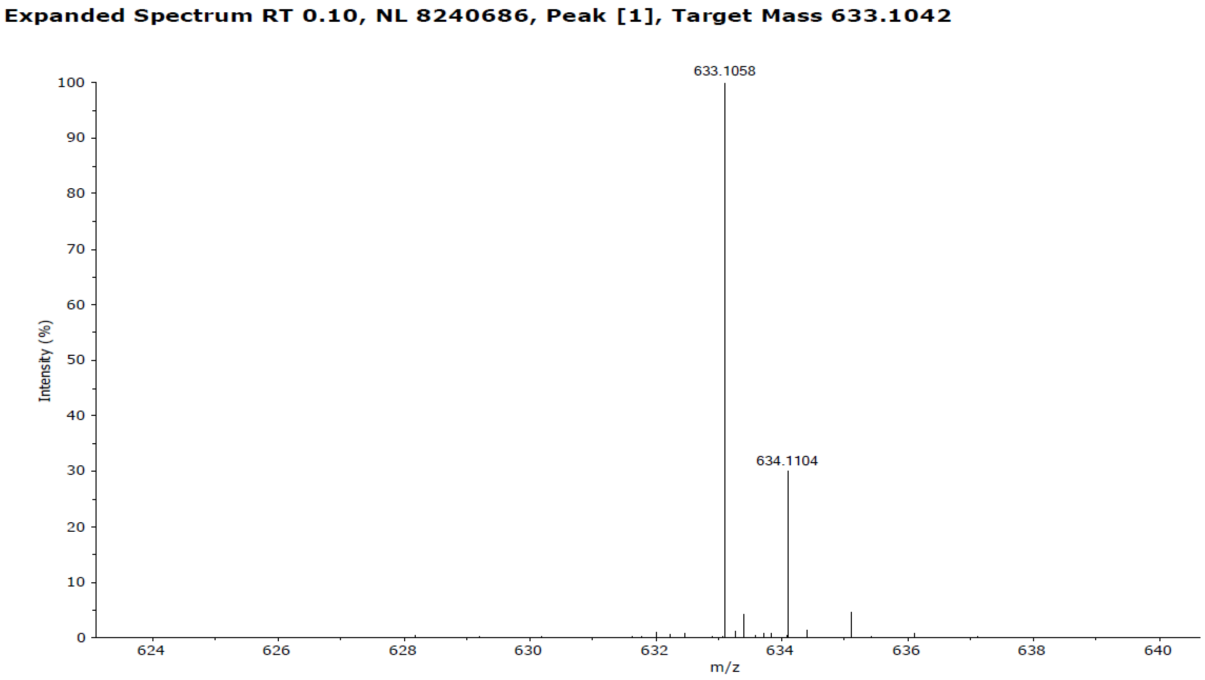

Figure S43: HRMS spectrum of **1.**

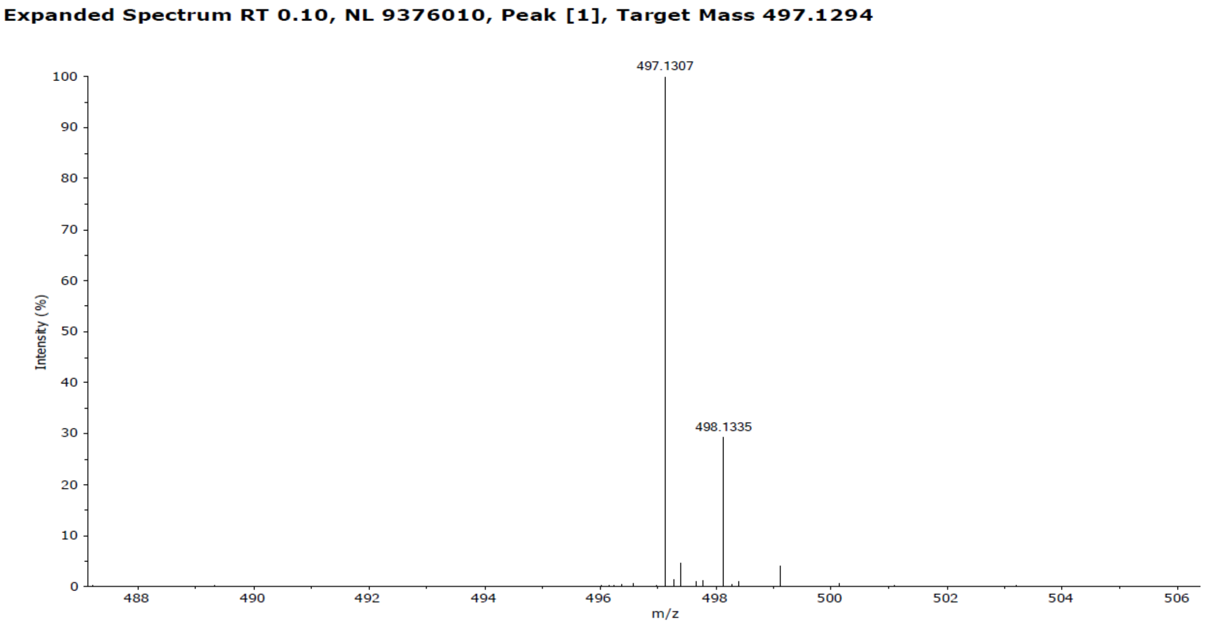

Figure S44: HRMS spectrum of **2.**

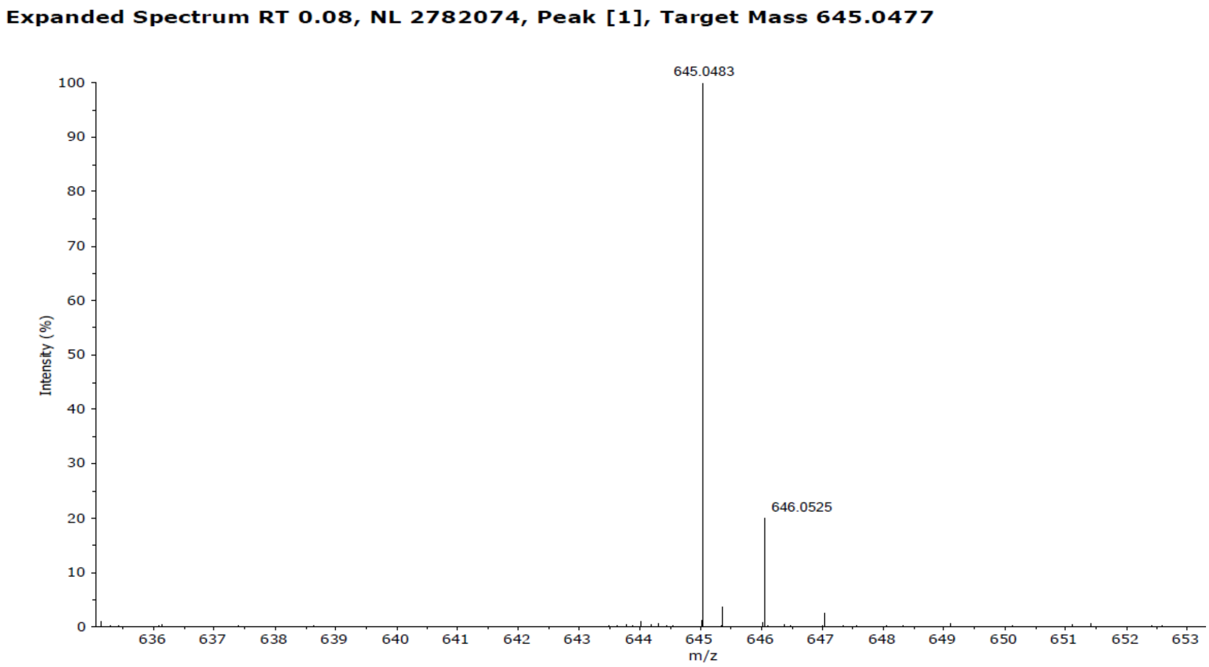

Figure S45: HRMS spectrum of **3.**

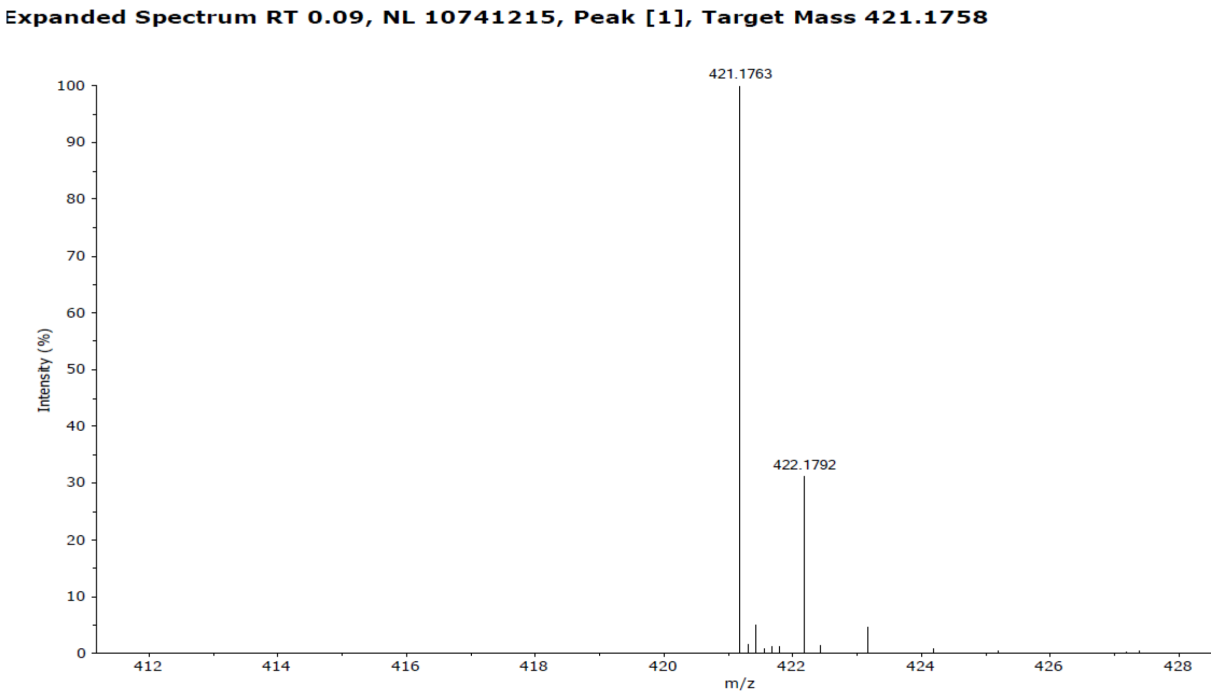

Figure S46: HRMS spectrum of **4.**

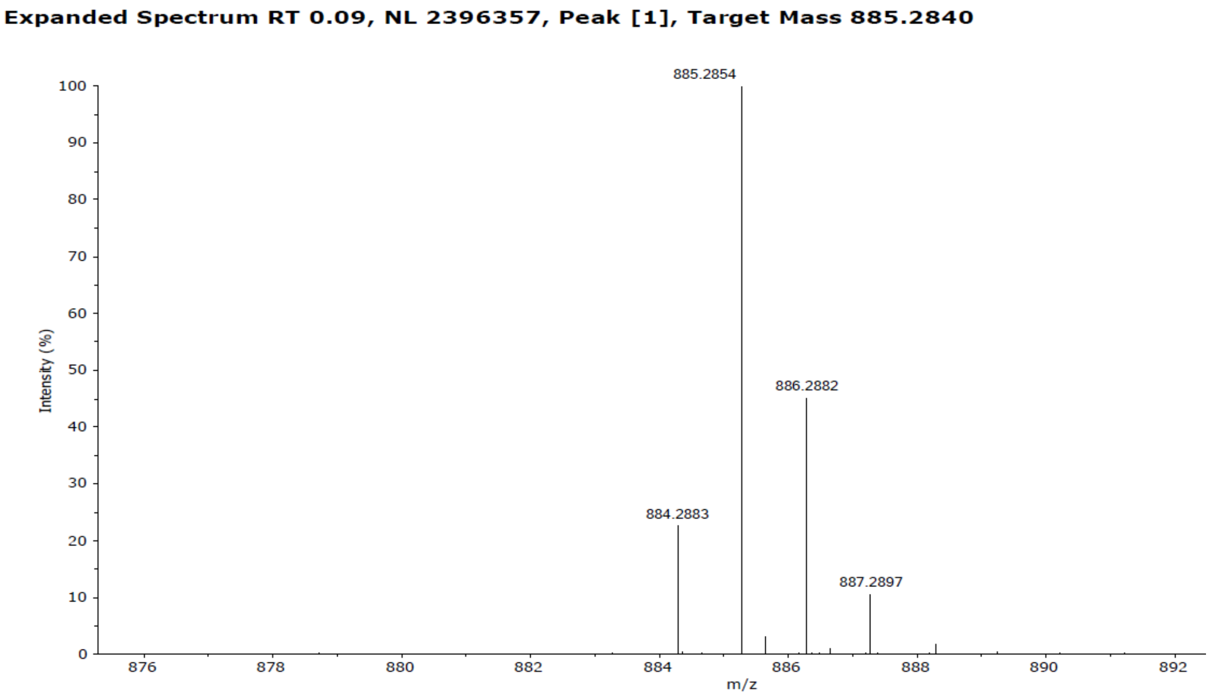

Figure S47: HRMS spectrum of **1a.**

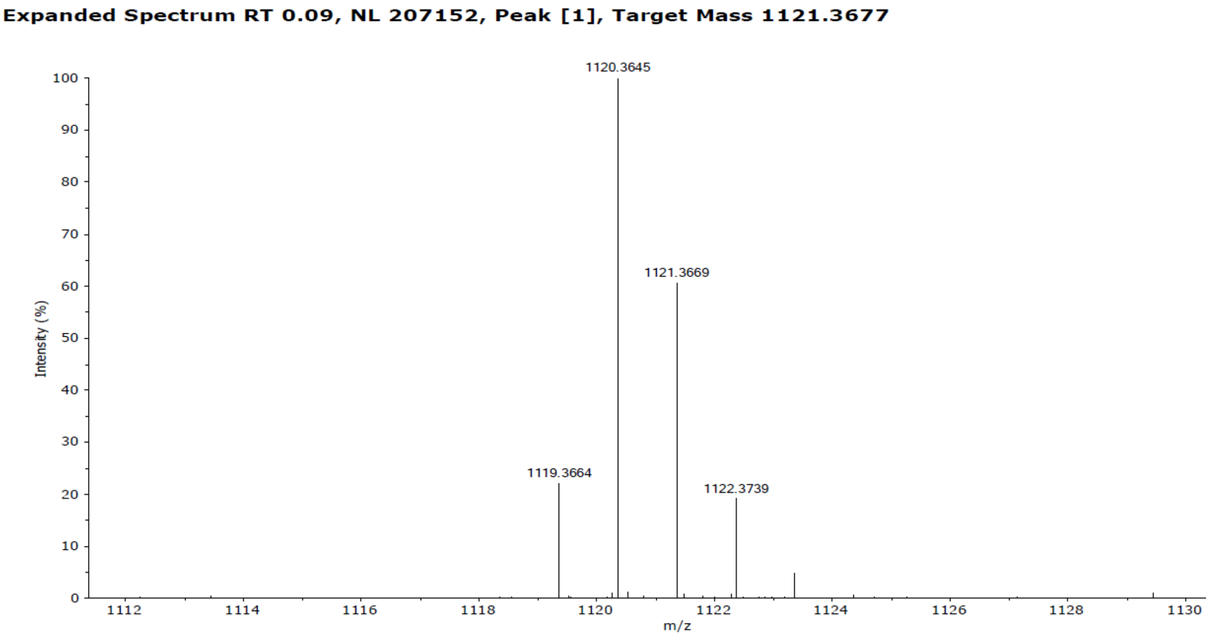

Figure S48: HRMS spectrum of **1b.**

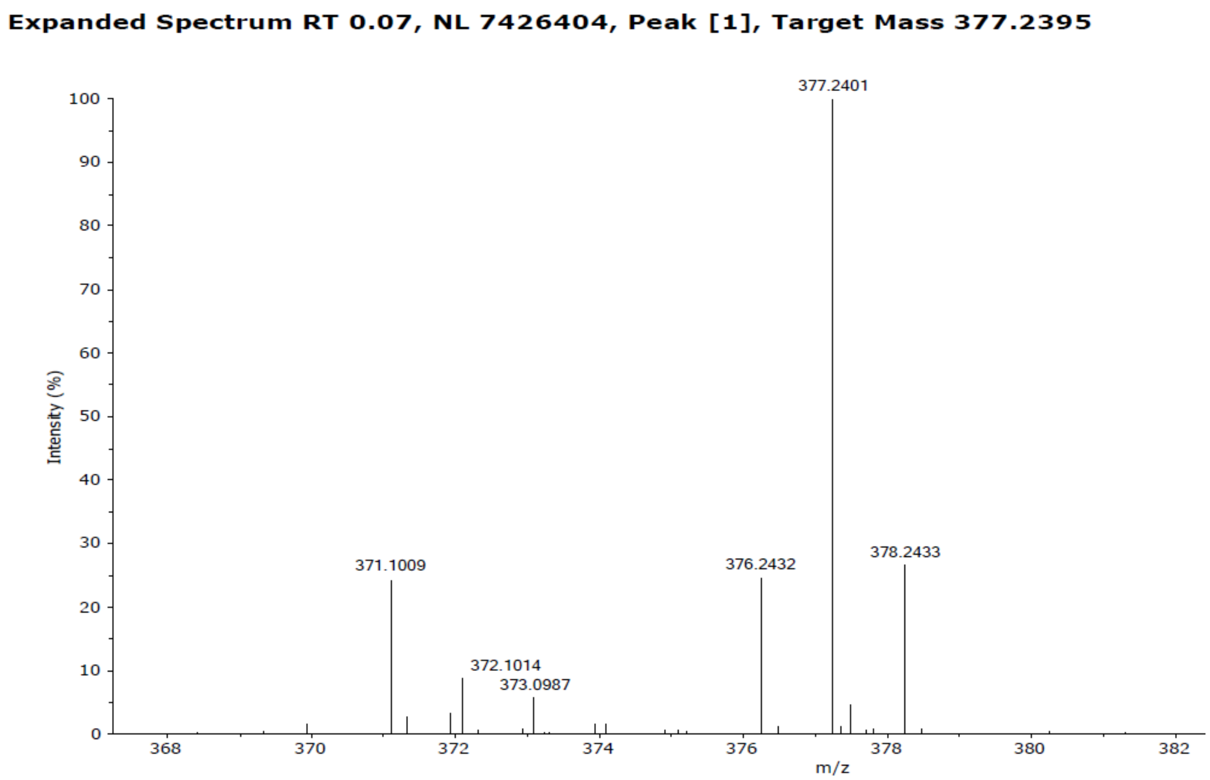

Figure S49: HRMS spectrum of control **C1**.

V. Anion Binding Studies

Transporters 1-4:

^{1}H NMR titration experiments were carried out at room temperature on a Bruker 400 MHz spectrometer. The residual solvent signal (CD_3CN, δ_H= 1.94 ppm) was considered as an internal reference to calibrate spectra. TBACl and the receptor were dried under a high vacuum before use. The titrations were performed by the addition of aliquots from the solution of TBACl (0.25 M in CD_3CN) to the solution of receptors, either of **1**, **2**, **3**, **4**, **1a** or **1b** (2.5 mM), respectively. All NMR data were processed using MestReNova 6.0, and the collected data were analyzed using BindFit v0.5.[4]

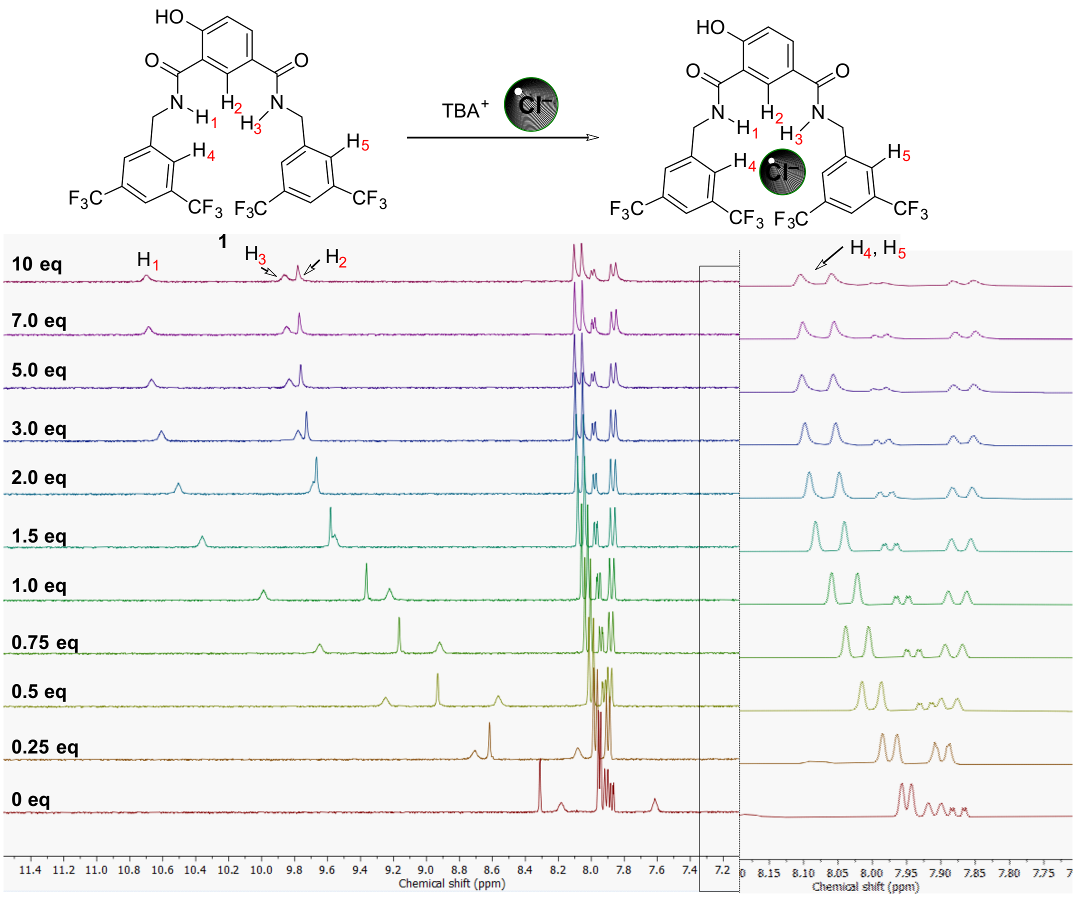

Figure S50. ^{1}H NMR titration spectra for **1** (2.5 mM in CD_3CN) with stepwise addition of TBACl in CD_3CN. The equivalents of added TBACl are shown on the stacked spectra.

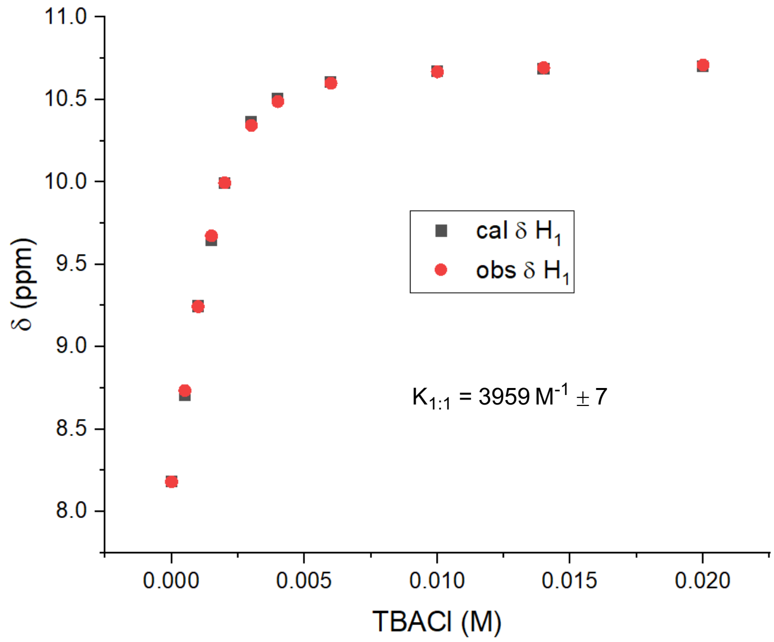

Figure S51. The plot of chemical shift (δ_H of H_1 proton *vs* concentration of TBACl added, fitted to 1:1 binding model of BindFit v0.5.

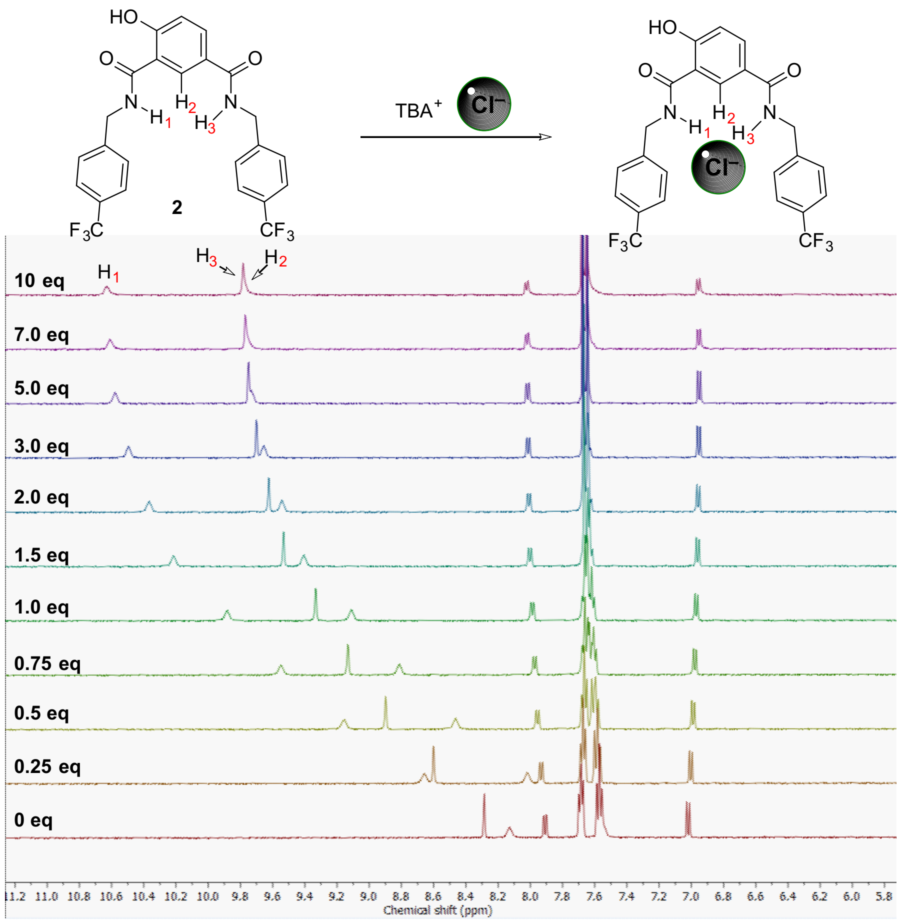

Figure S52. ^{1}H NMR titration spectra for **2** (2.5 mM in CD_3CN) with stepwise addition of TBACl in CD_3CN. The equivalents of added TBACl are shown on the stacked spectra.

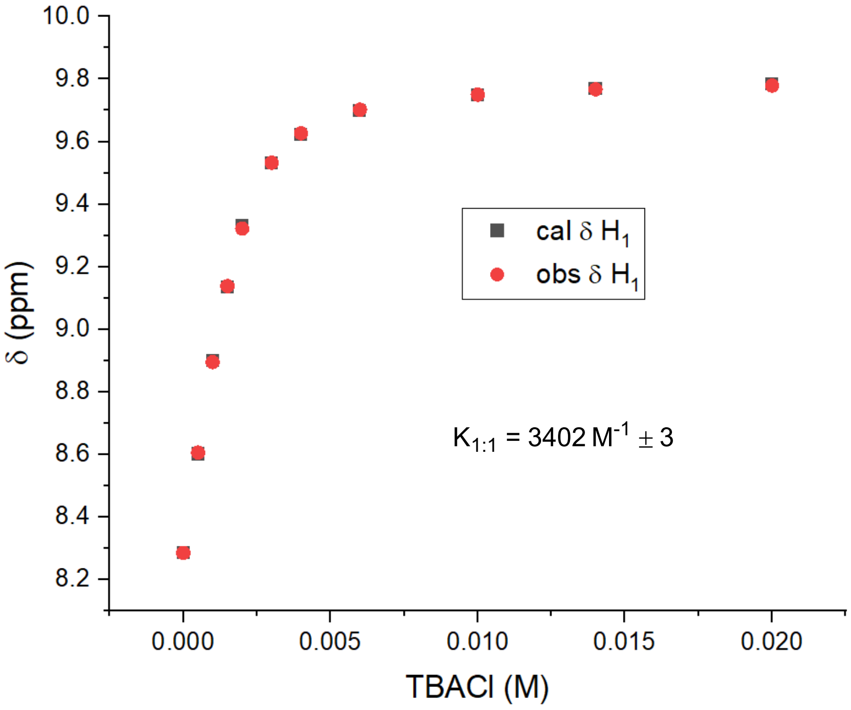

Figure S53. The plot of chemical shift (δ_H of H_1 proton *vs* concentration of TBACl added, fitted to 1:1 binding model of BindFit v0.5.

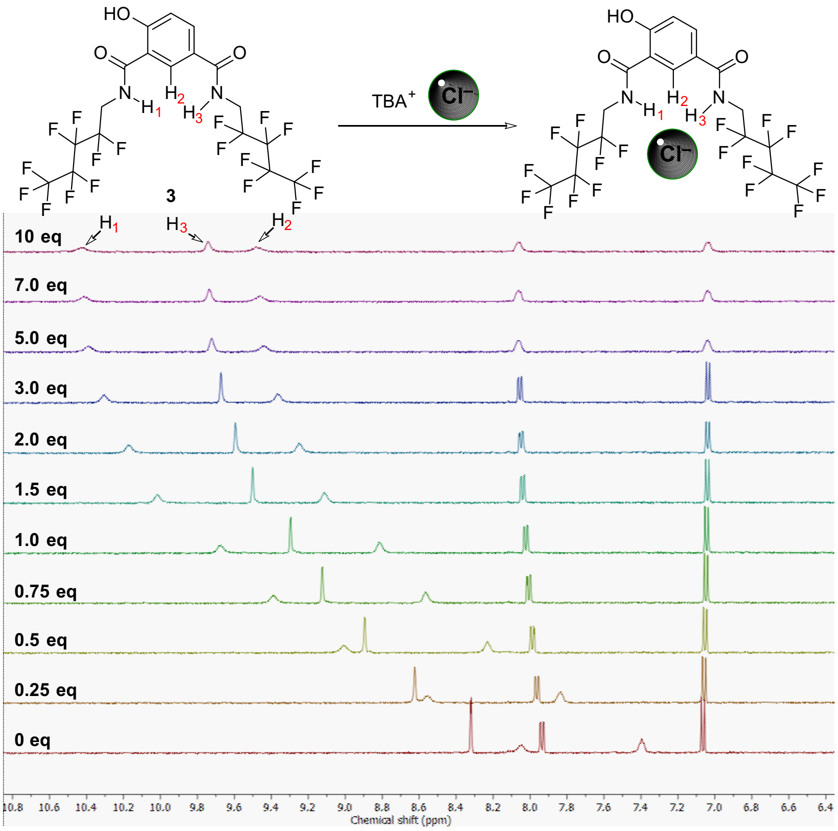

Figure S54. ^{1}H NMR titration spectra for **3** (2.5 mM in CD_3CN) with stepwise addition of TBACl in CD_3CN. The equivalents of added TBACl are shown on the stacked spectra.

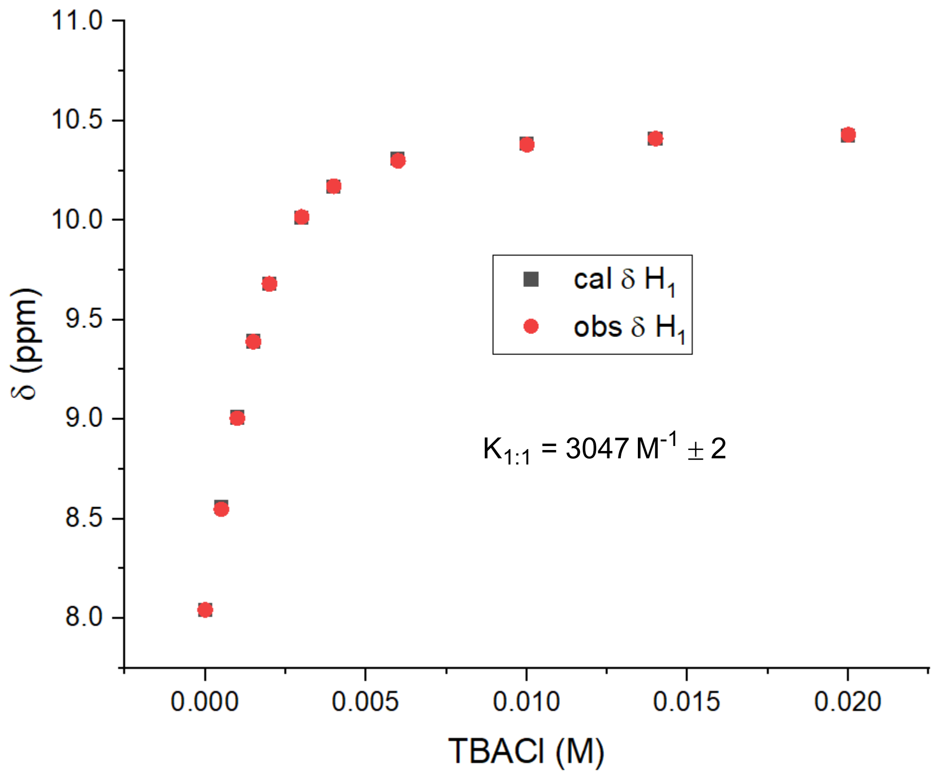

Figure S55. The plot of chemical shift (δ_H of H_1 proton *vs* concentration of TBACl added, fitted to 1:1 binding model of BindFit v0.5.

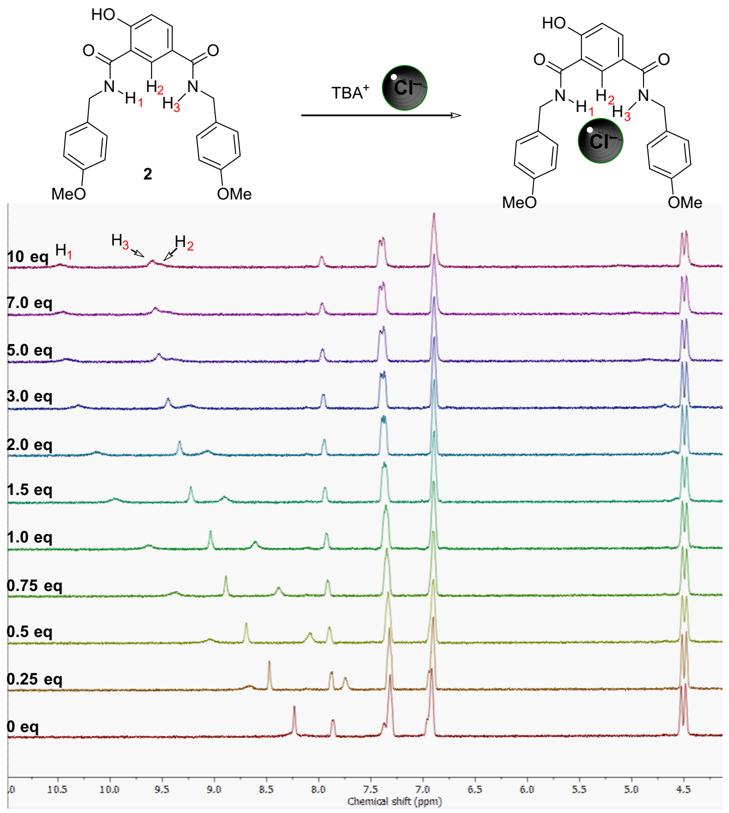

Figure S56. ^{1}H NMR titration spectra for **4** (2.5 mM in CD_3CN) with stepwise addition of TBACl in CD_3CN. The equivalents of added TBACl are shown on the stacked spectra.

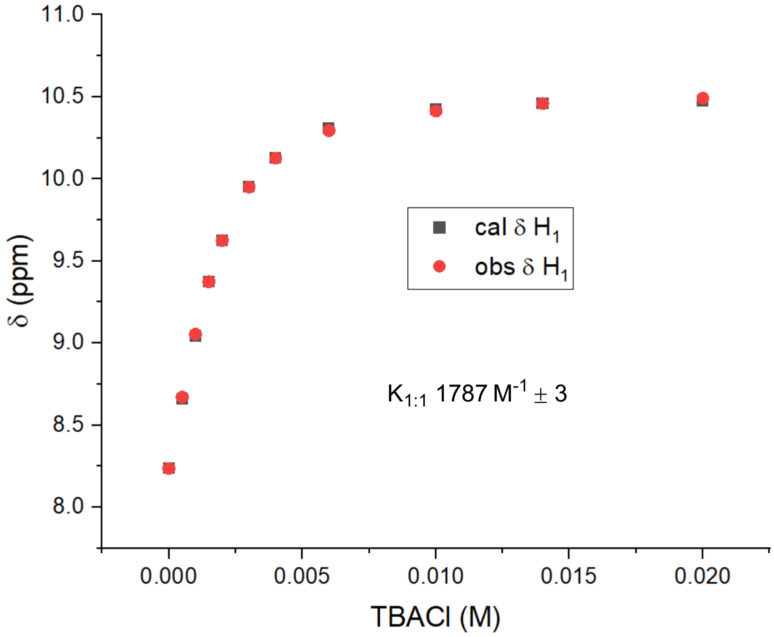

Figure S57. The plot of chemical shift (δ_H of H_1 proton *vs* concentration of TBACl added, fitted to 1:1 binding model of BindFit v0.5.

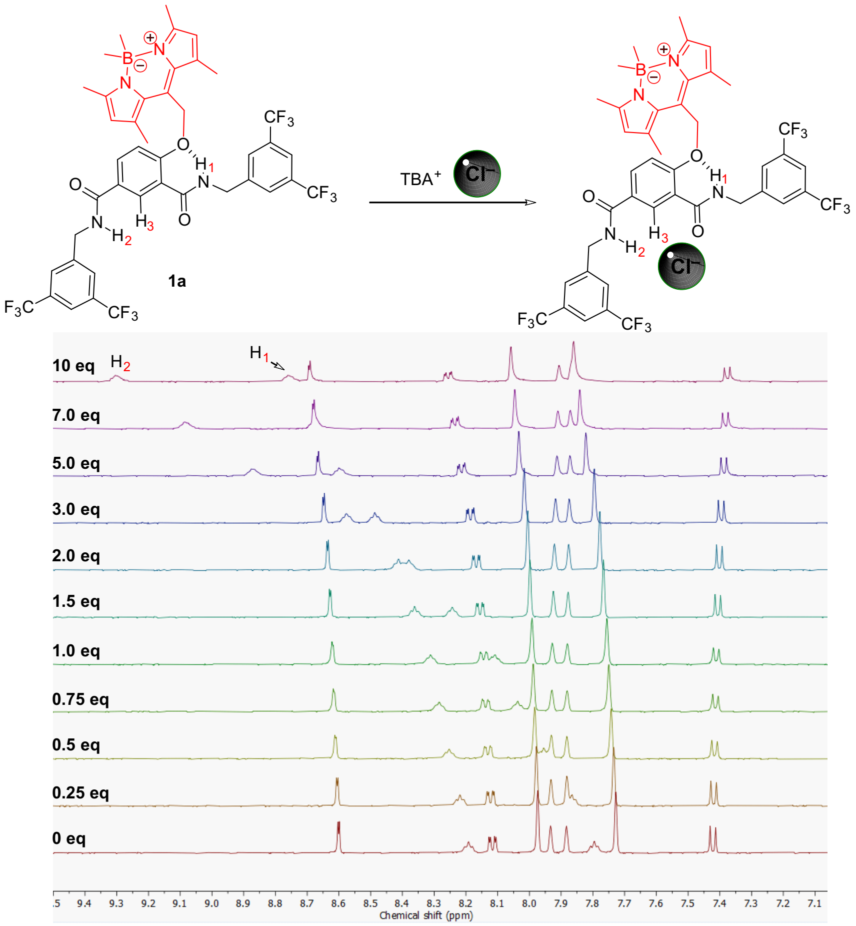

Figure S58. ^{1}H NMR titration spectra for **4** (2.5 mM in CD_3CN) with stepwise addition of TBACl in CD_3CN. The equivalents of added TBACl are shown on the stacked spectra.

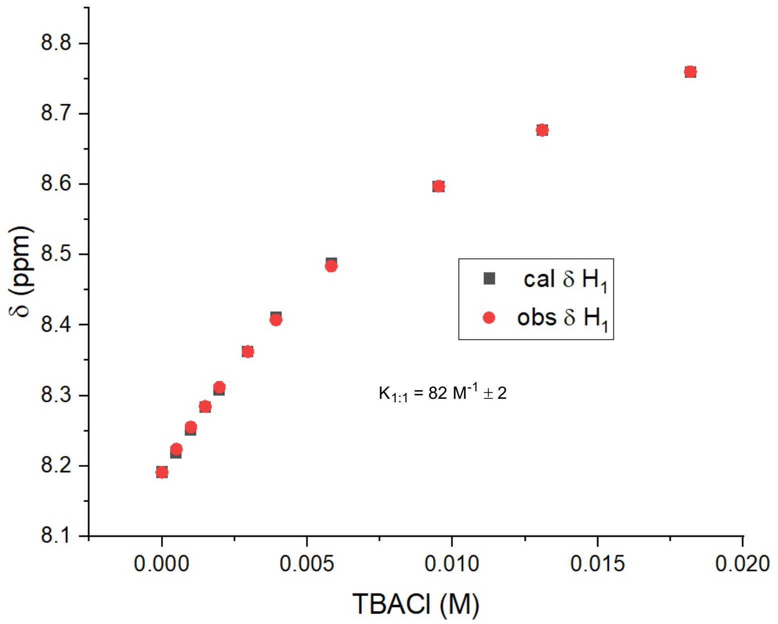

Figure S59. The plot of chemical shift (δ_H of H_1 proton *vs* concentration of TBACl added, fitted to 1:1 binding model of BindFit v0.5.

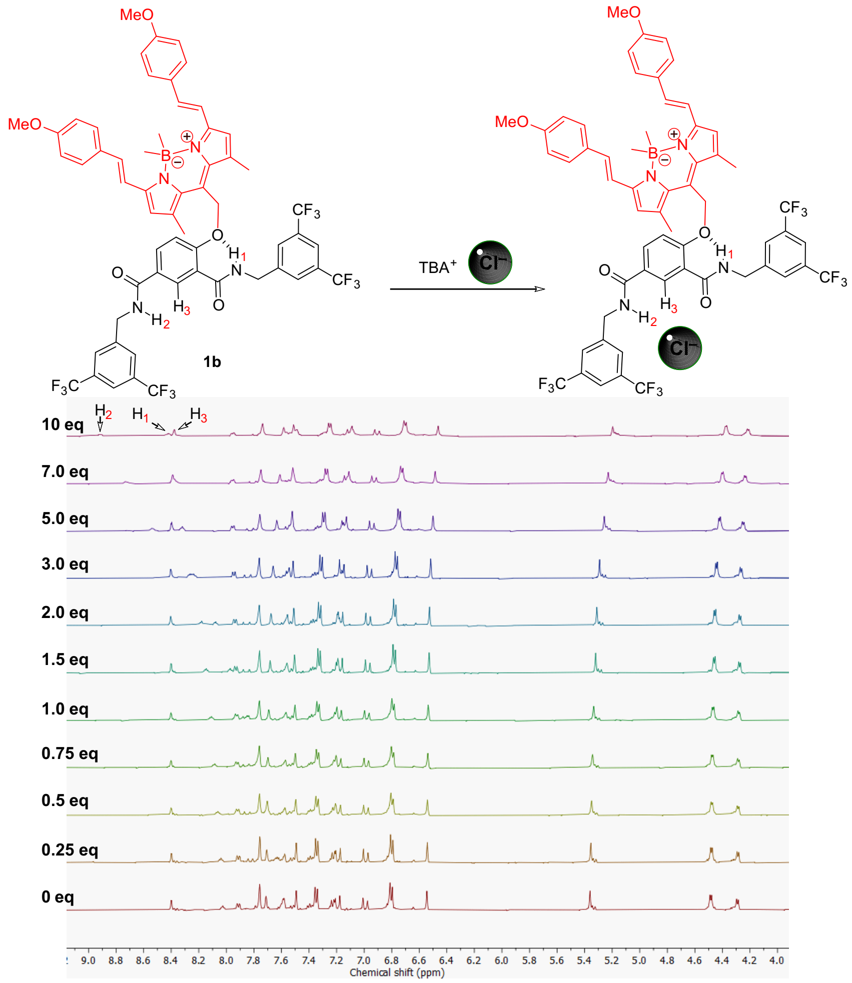

Figure S60. ^{1}H NMR titration spectra for **4** (2.5 mM in CD_3CN) with stepwise addition of TBACl in CD_3CN. The equivalents of added TBACl are shown on the stacked spectra.

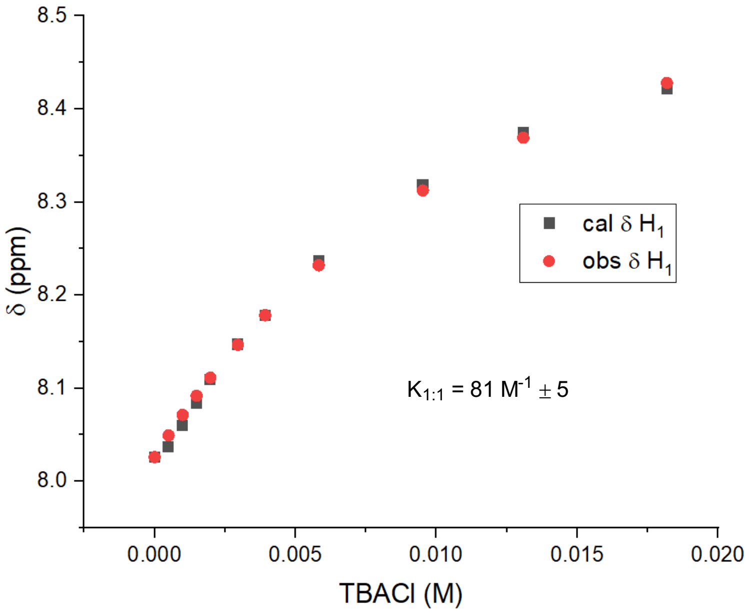

Figure S61. The plot of chemical shift (δ_H of H_1 proton *vs* concentration of TBACl added, fitted to 1:1 binding model of BindFit v0.5.

VI. Ion transport studies

Vesicle preparation

A thin film of lipid (1-palmitoyl-2-oleoyl-*sn*-3-phosphatidylcholine POPC, egg-yolk phosphatidylglycerol EYPG, or dipalmitoyl phosphatidylcholine DPPC) was formed by evaporating a chloroform solution under a stream of nitrogen gas, and then under high vacuum for 6 hours. The lipid film was hydrated by vortexing with the prepared buffer (100 mM NaCl, 10 mM HEPES, 1 mM 8-Hydroxypyrene-1,3,6-trisulfonic acid trisodium salt (HPTS), pH 7.0). The lipid suspension was then subjected to 5 freeze-thaw cycles using liquid nitrogen and a water bath (40°C), followed by extrusion 19 times through a polycarbonate membrane (pore size 200 nm) at rt. Extrusion was performed at 50°C in the case of DPPC lipids. Extra-vesicular components were removed by size exclusion chromatography on a Sephadex G-25 column with 100 mM NaCl, 10 mM HEPES, pH 7.0. Final conditions: LUVs (2.5 mM lipid); inside 100 mM NaCl, 10 mM HEPES, 1 mM HPTS, pH 7.0; outside: 100 mM NaCl, 10 mM HEPES, pH 7.0. Vesicles for the sodium gluconate assay were prepared by the same procedure, substituting NaCl for NaGluconate in the buffer solution.

Transport assays with HPTS

In a typical experiment, the LUVs containing HPTS (40 µL, final lipid concentration 31.3 µM) were added to buffer (2910 µL of 100 mM NaCl, 10 mM HEPES, pH 7.0) at 25°C under gentle stirring. A pulse of NaOH (30 µL, 0.5 M) was added at 20 s to initiate the experiment. At 100 s the test transporter was added, followed by detergent (40 µL of Triton X-100 in 7:1 (v/v) H_2O-DMSO) at 300 secs to calibrate the assay. The fluorescence emission was monitored at λ_{em} = 510 nm (λ_{ex} = 460/405 nm). The fractional fluorescence intensity (I_{rel}) was calculated from equation (S1), where R_t is the fluorescence ratio at time t, (ratio of intensities 460 nm /

405 nm excitation) R_0 is the fluorescence ratio at time 77 s, and R_d is the fluorescence ratio after the addition of detergent.

$$I_{rel} = \frac{R_t - R_0}{R_d - R_0} \qquad \textbf{(S1)}$$

The fractional fluorescence intensity (I_{rel}) at 290 s just prior to lysis, defined as the fractional activity y, was plotted as a function of the ionophore concentration (x / μM). Hill coefficients (n) and EC_{50} values were calculated by fitting to the Hill equation (S2) equation,

$$y = y_0 + (y_{max} - y_0).\frac{x^n}{EC50^n + x^n} \qquad \textbf{(S2)}$$

where y_0 is the fractional activity in the absence of transporter, y_{max} is the fractional activity in with excess transporter, and x is the transporter concentration in the cuvette.

For all compounds, each individual concentration was repeated at least three times and averaged; error bars represent standard deviations.

Experiments with DPPC lipids were conducted in the same way. For elevated temperature studies, the sample was equilibrated at 45°C (using the Peltier temperature controller) for 5 minutes prior to initiating the experiment.

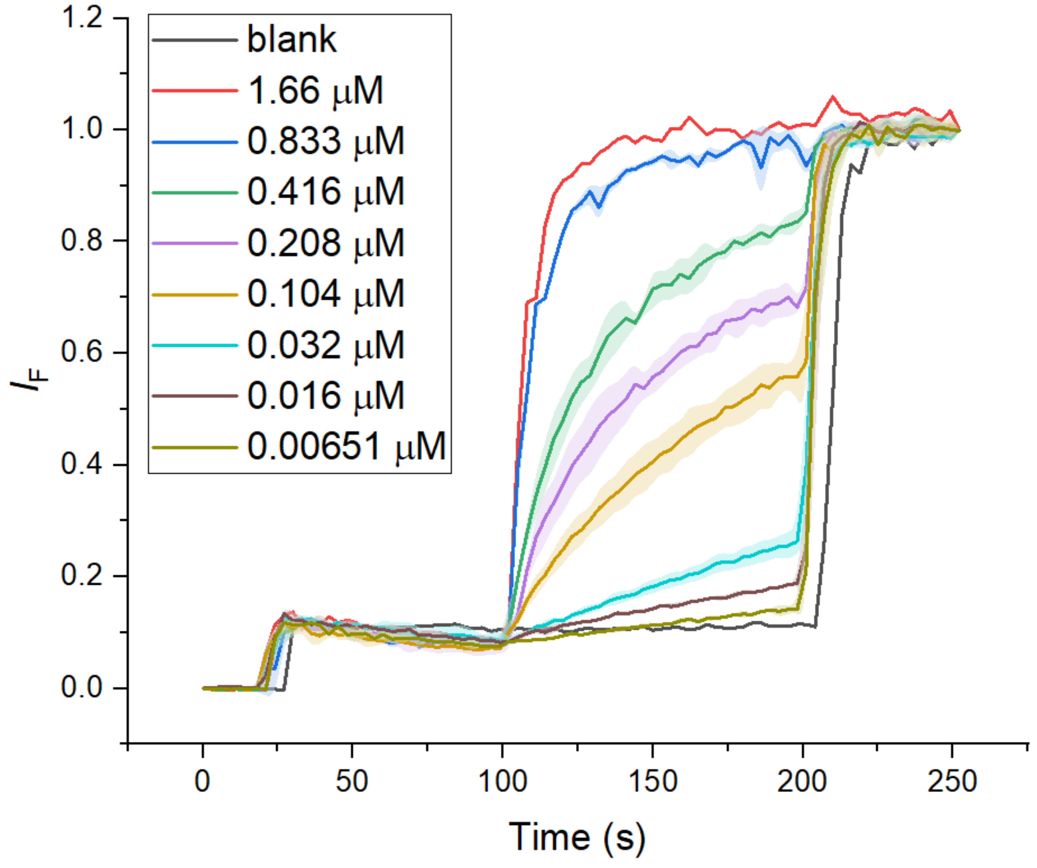

Figure S62. Ion transport HPTS assay data for **1** in POPC LUVs.

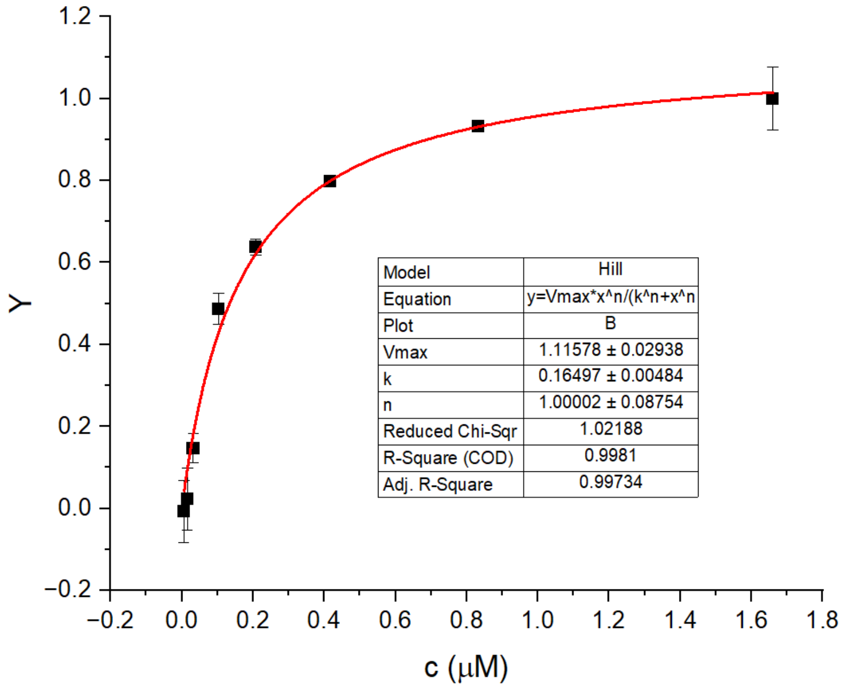

Figure S63. Hill plot for **1**.

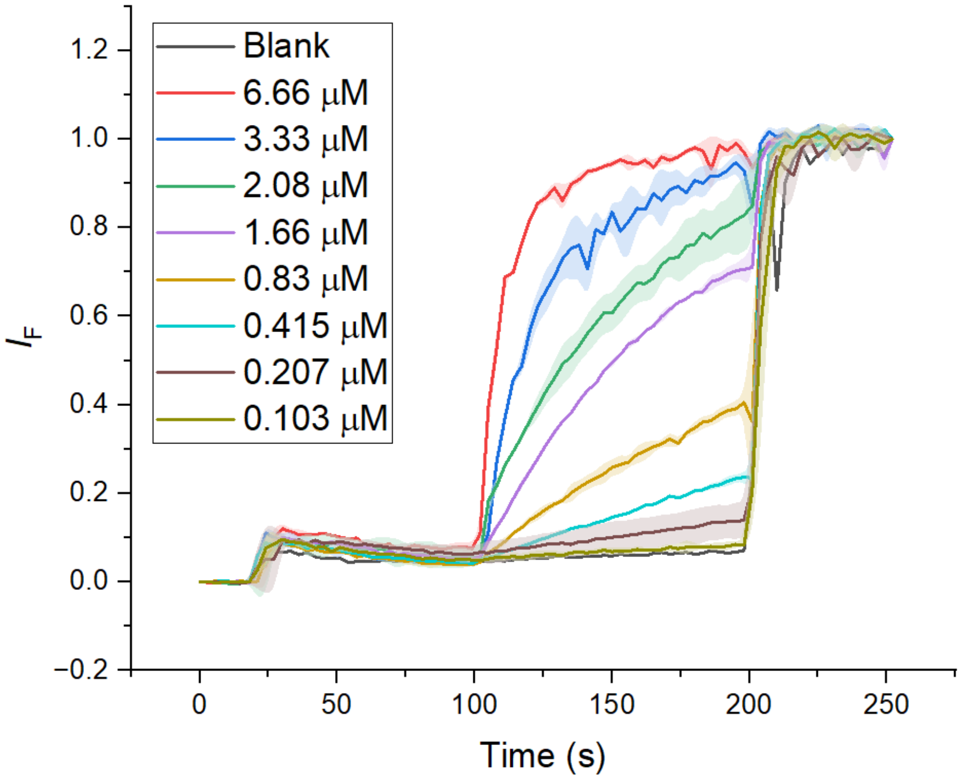

Figure S64. Ion transport HPTS assay data for **2** in POPC LUVs.

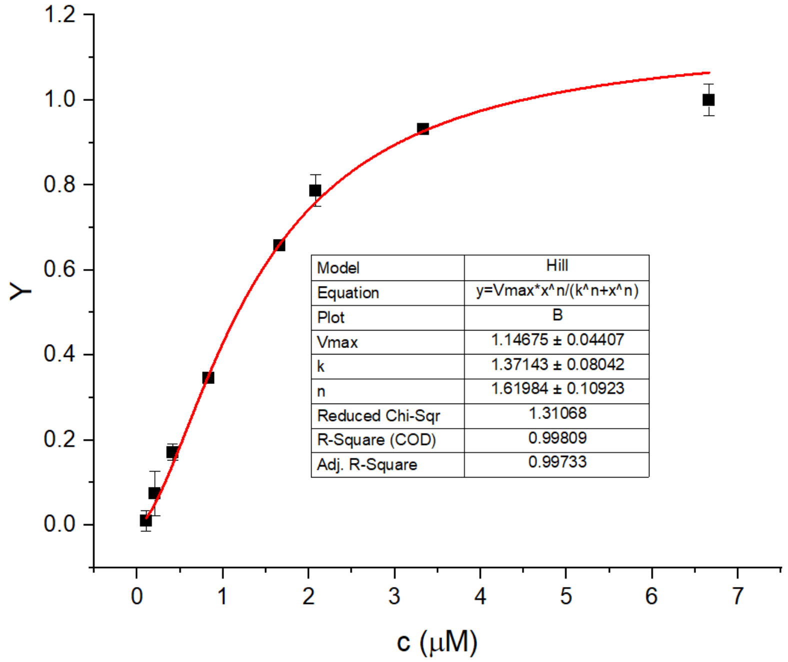

Figure S65. Hill plot for **2**.

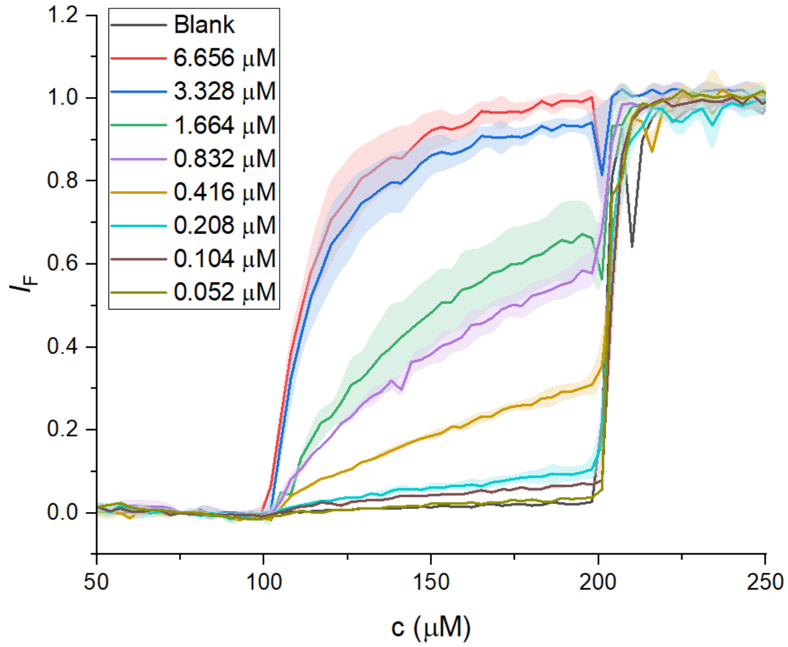

Figure S66. Ion transport HPTS assay data for **3** in POPC LUVs.

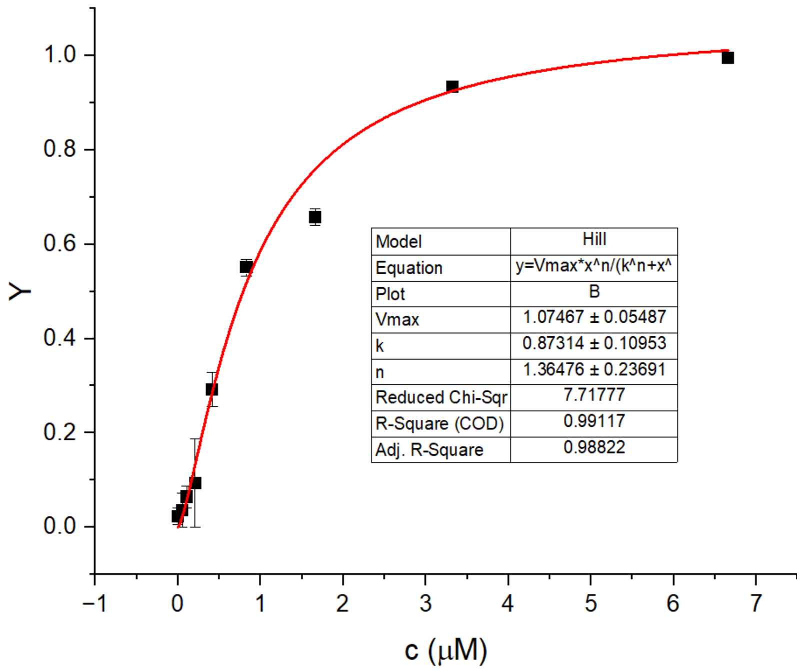

Figure S67. Hill plot for **3**.

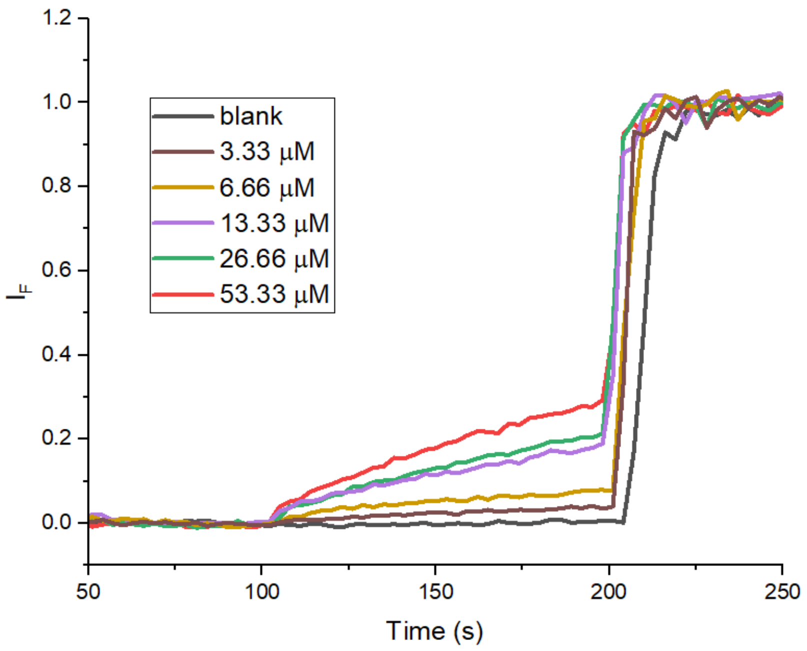

Figure S68. Ion transport HPTS assay data for **4** in POPC LUVs.

Effect of external ion exchange

The effect of external ion exchange was explored by using a previously reported external ion exchange assay.[5-7] These experiments were carried out by adding the POPC vesicle solution (prepared as above) to buffer (100 mM MX, 10 mM HEPES, pH 7.0), where M = Li, Na, K, Rb, Cs (X = Cl), and X = Cl, Br, I, NO_3, and ClO_4 (M = Na).

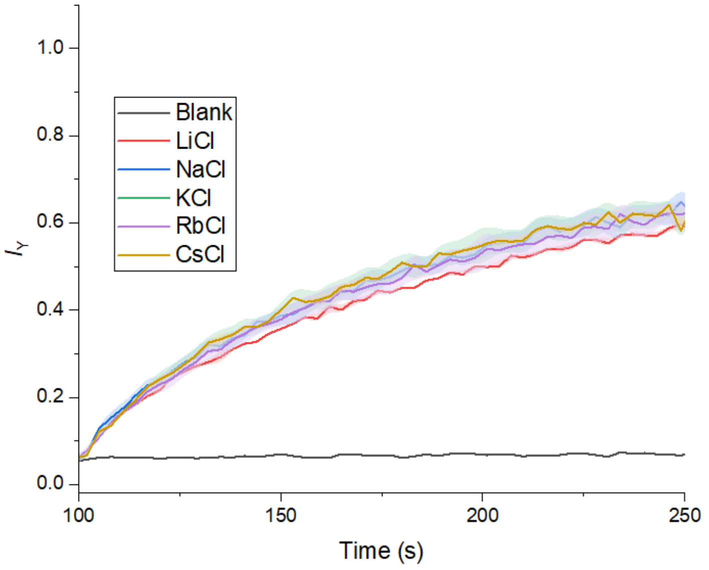

Figure S69. HPTS assay of **1** at 3 mol% carrier concentration and varying external anions (external buffer: 100 mM MCl (M = Li, Na, K, Rb, and Cs, 10 mM HEPES).

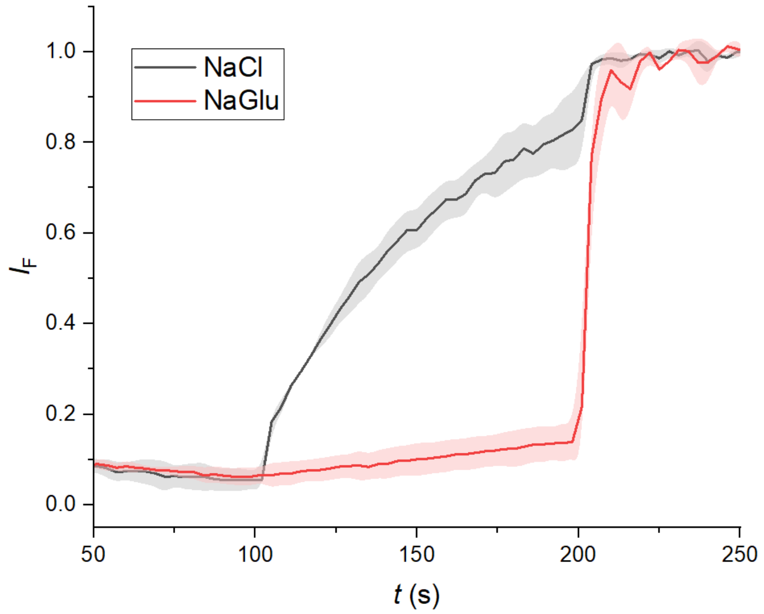

Figure S70. HPTS transport activity of **1** (0.414 mM) in presence of either internal NaCl or Na-Gluconate solutions.

Chloride transport activity across POPC-LUVs⊂lucigenin vesicles

Preparation of POPC-LUVs⊂lucigenin vesicles: In a 10 mL clean and dry round-bottom flask, the thin transparent film of egg yolk phosphatidylcholine (POPC) was formed by drying 1.0 mL egg yolk phosphatidylcholine (POPC, 25 mg/mL in $CHCl_3$) whilst providing continuous rotation and purging nitrogen. The transparent thin film was kept under a high vacuum for 4 hours to remove all traces of $CHCl_3$, before it was hydrated with 1.0 mL aqueous $NaNO_3$ (200 mM, 1.0 mM Lucigenin) with a pH of 6.5 using 10 mM phosphate buffer with occasional vortexing at 10 min intervals for 1 h. The resulting suspension was subjected to freeze and thaw cycles (≥ 10 liquid nitrogen, 55 °C water bath) and 21 times extrusion through a 200 nm pore size polycarbonate membrane. Size exclusion chromatography (using Sephadex G-50) was performed to remove extravesicular dye using a 200 mM $NaNO_3$ solution as eluent. The collected vesicle suspension was diluted to 4 mL. Final conditions: ~ 5 mM POPC; inside: 200 mM $NaNO_3$, 1 mM lucigenin, pH = 6.5; outside: 200 mM $NaNO_3$, pH = 6.5.

Ion transport activity by Lucigenin assay

In a clean and dry fluorescence cuvette, 200 mM $NaNO_3$ (2890 µL), POPC-LUVs⊃lucigenin (40 µL, 109.6 µM), and ion transporter **1** (20 µL DMF solution, at t = 50 s) were added. This suspension was placed in a slowly stirring condition in a fluorescence instrument equipped with a magnetic stirrer (at t = 0 s). The fluorescence intensity of lucigenin was monitored at λ_{em} = 535 nm (λ_{ex} = 455 nm) over time. The chloride gradient was created by the addition of 2.0 M NaCl (50 µL) at t = 50 s between intra- and extra-vesicular solutions. Finally, vesicles were lysed by adding 10% Triton X-100 (40 µL) at t = 150 s for the complete destruction of the chloride gradient.

The time-dependent data were normalized to percent change in fluorescence intensity using Equation S3:

$$I_F = [(I_t - I_0) / (I_\infty - I_0)] \times (-1) \qquad \text{Equation S3}$$

where, I_0 is the initial intensity, I_t is the intensity at time t, and I_∞ is the final intensity after addition of Triton X-100.

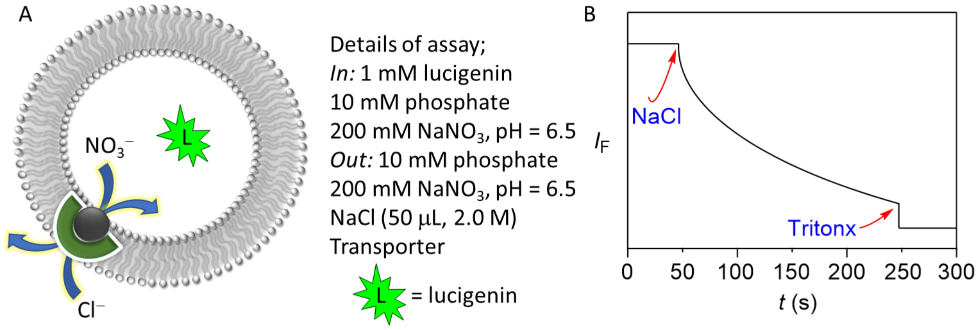

Figure S71. Representation of fluorescence-based ion transport activity assay using POPC-LUVs⊃Lucigenin (A), and illustration of ion transport kinetics showing normalization window (B).

Dose-response activity: The activity of each transporter at different concentrations was studied. The concentration profile data were evaluated from the fluorescence intensity at t = 240 s to afford the effective concentration, EC_{50} (i.e., the concentration of transporter needed to achieve 50% chloride efflux) using the Hill equation (Equation S4):

$$Y = Y_\infty + (Y_0 - Y_\infty) / [1 + (c/EC_{50})^n] \qquad \text{Equation S4}$$

where Y_0 = fluorescence intensity just before the transporter addition (at t = 0 s), Y_∞ =fluorescence intensity with excess transporter concentration, c = concentration of transporter compound, and n = Hill coefficient (i.e., indicative of the number of monomers needed to form an active supramolecule).

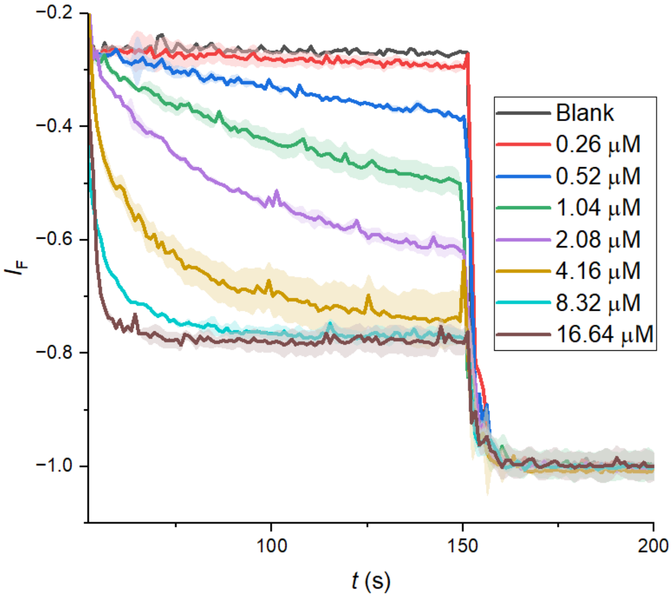

Figure S72: Concentration dependent activity of **1** across POPC-LUVs⊃lucigenin

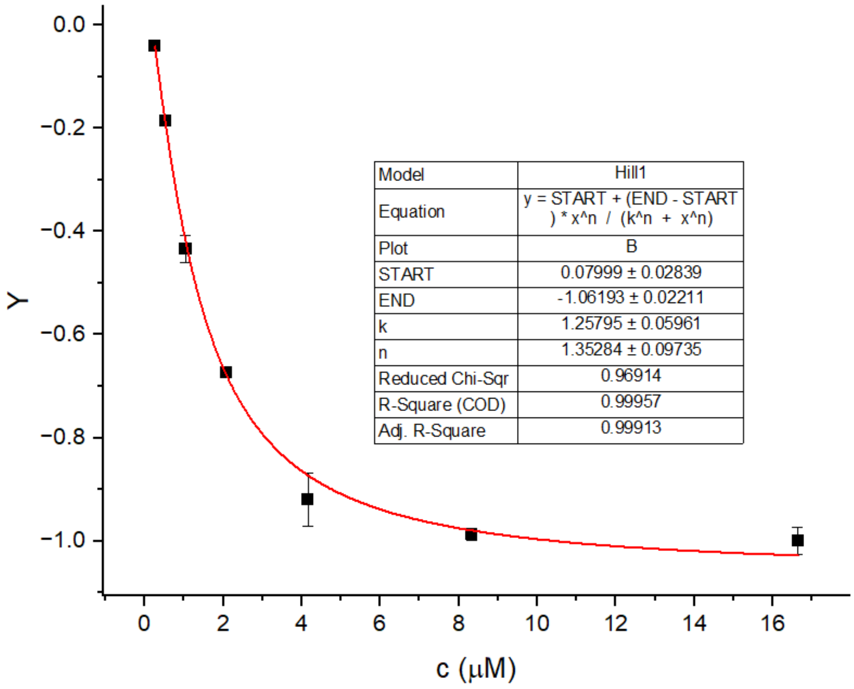

Figure S73: Dose response plot of **1** at 240 s.

Lucigenin assay with external SO_4^{2-} and NO_3^- anions

Preparation of POPC-LUVs⊃lucigenin vesicles: In a 10 mL clean and dry round-bottom flask, the thin transparent film of egg yolk phosphatidylcholine (POPC) was formed by drying 1.0 mL egg yolk phosphatidylcholine (POPC, 25 mg/mL in $CHCl_3$) while providing continuous rotation and purging nitrogen. The transparent thin film was kept under a high vacuum for 4 hours to remove all traces of $CHCl_3$. Then the transparent thin film was hydrated with 1.0 mL aqueous NaCl (200 mM, 1.0 mM Lucigenin) buffered at pH of 6.5 with occasional vortexing at 10 min intervals for 1 h. The resulting suspension was subjected to freeze and thaw cycles (≥ 10 liquid nitrogen, 55 °C water bath) and 21 times extrusion through a 200 nm pore size polycarbonate membrane. The size exclusion chromatography (using Sephadex G-50) was performed to remove extravesicular dye using a 200 mM NaCl solution as eluent. The collected vesicle suspension was diluted to 4 mL. Final conditions: ~ 5 mM POPC; inside: 200 mM NaCl, 1 mM lucigenin, pH 6.5; outside: either 200 mM $NaNO_3$ or 200 mM Na_2SO_4.

Ion transport assay

In a clean and dry fluorescence cuvette, either 200 mM of $NaNO_3$ or 200 mM of Na_2SO_4 (2890 µL), and POPC-LUVs⊃lucigenin (20 µL, 54.8 µM) was added. This suspension was placed in a slowly stirring condition in a fluorescence instrument equipped with a magnetic stirrer (at t = 0 s). The fluorescence intensity of lucigenin was monitored at λ_{em} = 535 nm (λ_{ex} = 455 nm) as a course of time. The transporter molecule **1** (7.63 mol%) was added at t = 50 s. Finally, vesicles were lysed by adding 10% Triton X-100 (40 µL) at t = 150 s for the complete destruction of the chloride gradient.

The time-dependent data were normalized to percent change in fluorescence intensity using Equation S5.

$$I_F = [(I_t - I_0) / (I_\infty - I_0)] \times (1) \qquad \text{Equation S5}$$

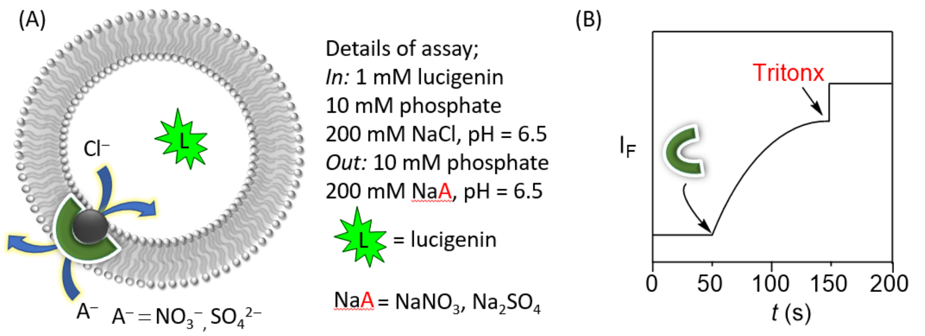

Figure S74. (A) Representation of fluorescence-based antiport assay using POPC-LUVs⊃lucigenin. (B) Representation of ion transport kinetics showing normalization window

VII. Photoresponsive studies

UV-Vis absorption studies

The UV-Vis absorption studies of compounds **1a** and **1b** were carried out in CH_3CN. Initially, stock solutions of these compounds (1 mM in CH_3CN) were prepared in different glass vials and covered with an aluminum foil. In a 3 mL UV cuvette, 2900 µL of CH_3CN was placed, and either **1a** or **1b** was added to a final concentration of 100 µM. The cuvette was placed in a UV-Vis spectrometer, and the UV-Vis spectrum was recorded.

Assessment of triggered release of **1** from **1a** or **1b** using photoirradiation:
In a clean and dry NMR tube, the solution of **1a** was taken in DMSO-d_6 (1 mM in 0.5 mL). The ^{1}H NMR spectrum of the sample was recorded first ($t = 0$ min). Then, the NMR tube containing the compound **1a** was photoirradiated using a 530 nm LED (480 mW) for different time intervals. The ^{1}H NMR spectrum of the irradiated samples was recorded at the end of each irradiation. All ^{1}H NMR spectra were processed using MestReNova 6.0 by considering the residual solvent peak as an internal reference. Finally, the NMR spectra of **1a** and the photoirradiated samples were stacked and compared with that of synthesized **1** (recorded in DMSO-d_6). Upon photoirradiation, the appearance and disappearance of the different proton peak signals of the protransporter **1a** indicated the release of the as-synthesized active transporter **1**.

For protransporter **1b**, the sample was photoirradiated in DMSO-d_6 (1 mM in 0.5 mL) with 625 nm (920 mW) or 730 nm (1130 mW) LEDs.

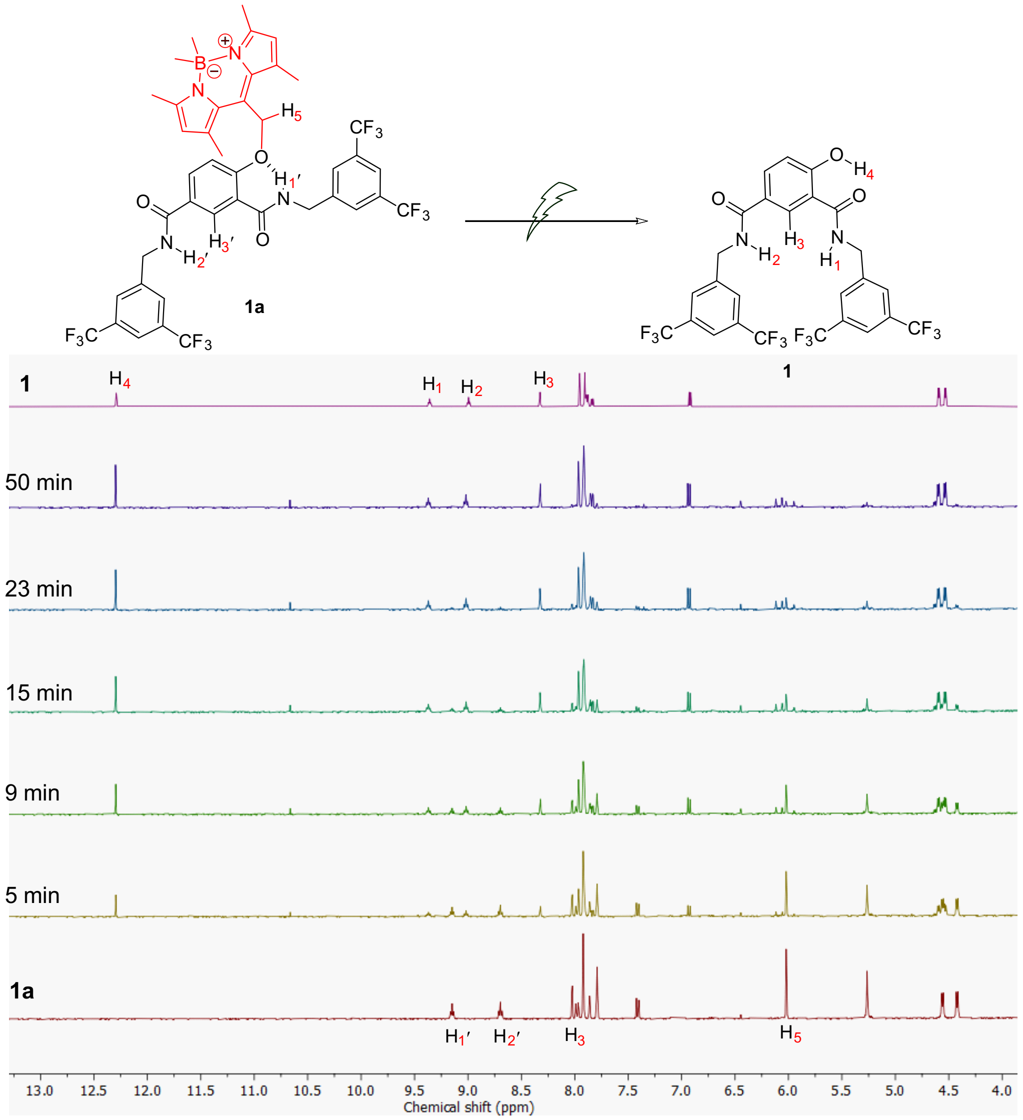

Figure S75. Photo release of active transporter **1** from protransporter **1a** upon photoirradiation using 530 nm LED (480 mW) recorded in DMSO-d_6.

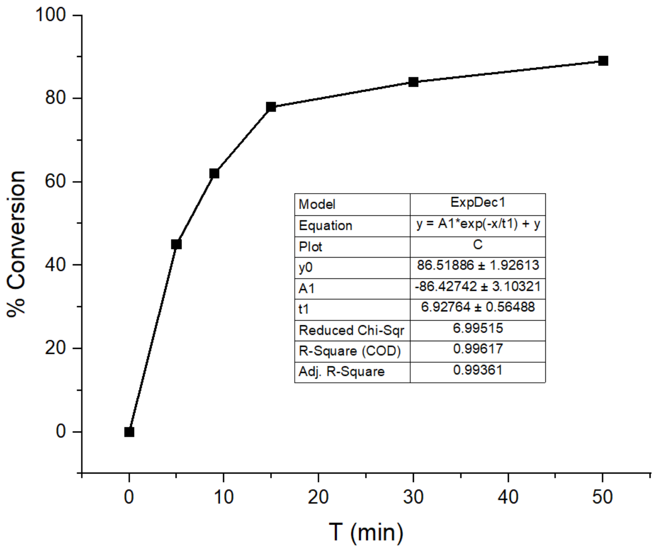

Figure S76: % deprotection of **1a** under 530 nm irradiation.

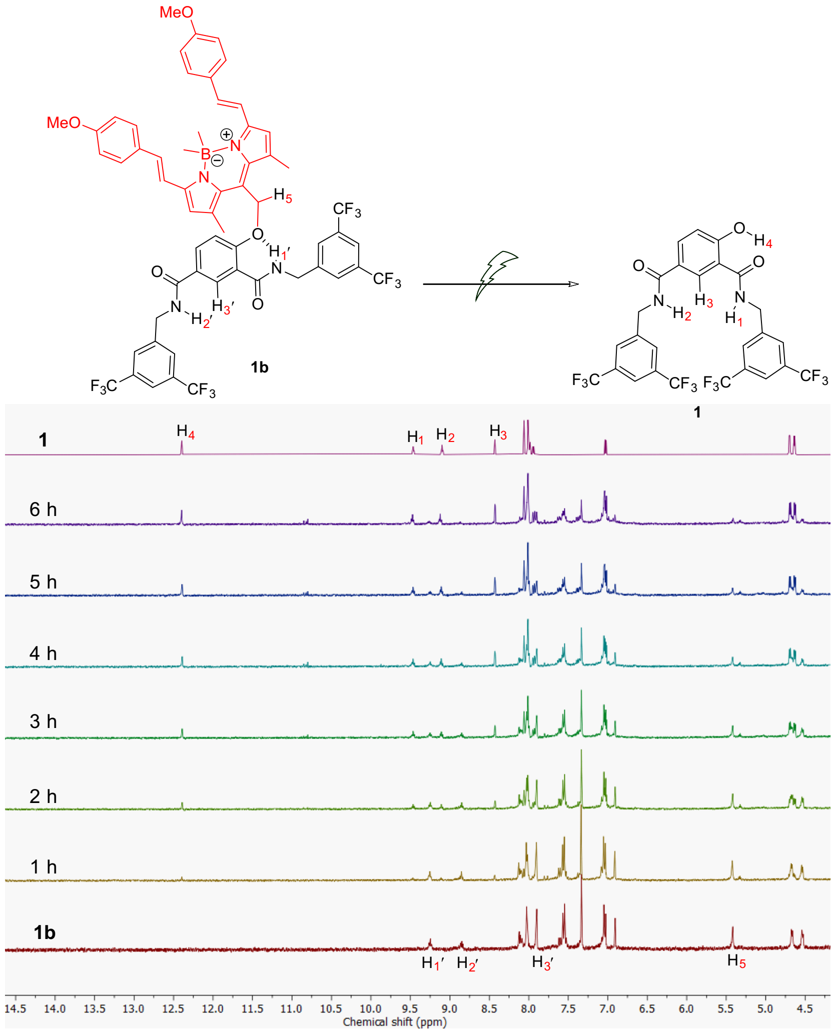

Figure S77. Photo release of active transporter **1** from protransporter **1b** upon photoirradiation using a 625 nm LED (920 mW) recorded in DMSO-d_6.

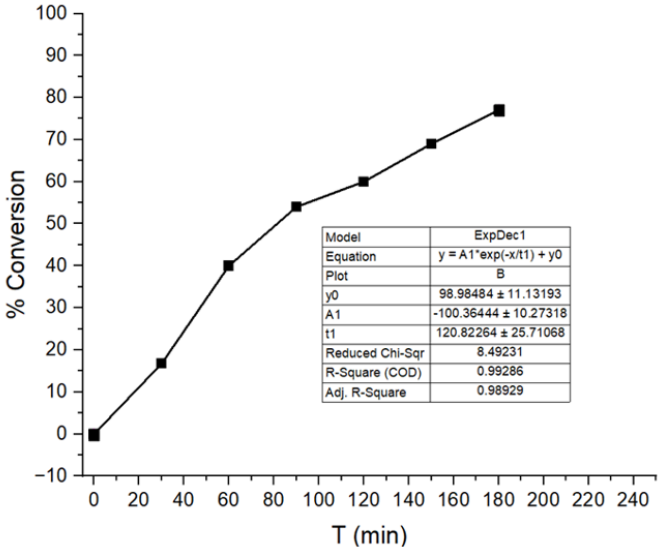

Figure S78: % deprotection of **1b** under 625 nm irradiation.

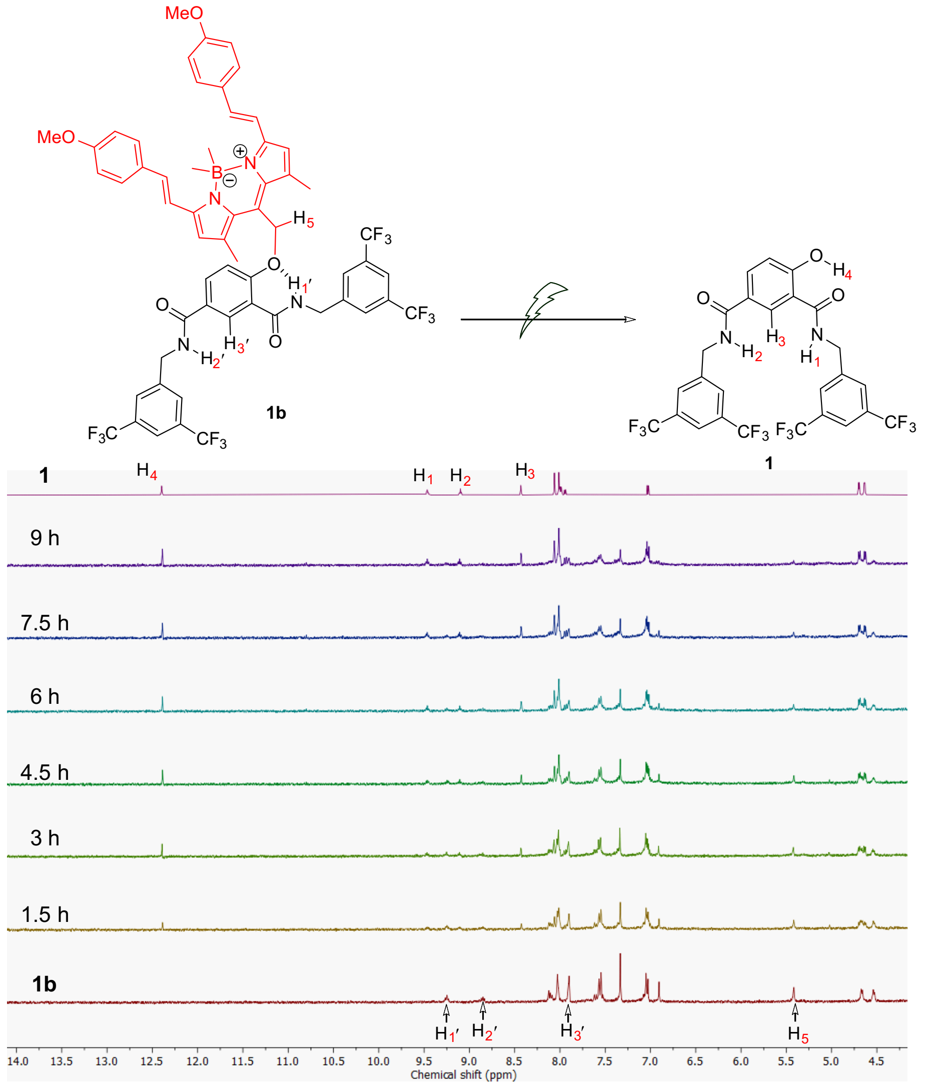

Figure S79. Photo release of active transporter **1** from protransporter **1b** upon photoirradiation using 730 nm LED (1130 mW) recorded in DMSO-d_6.

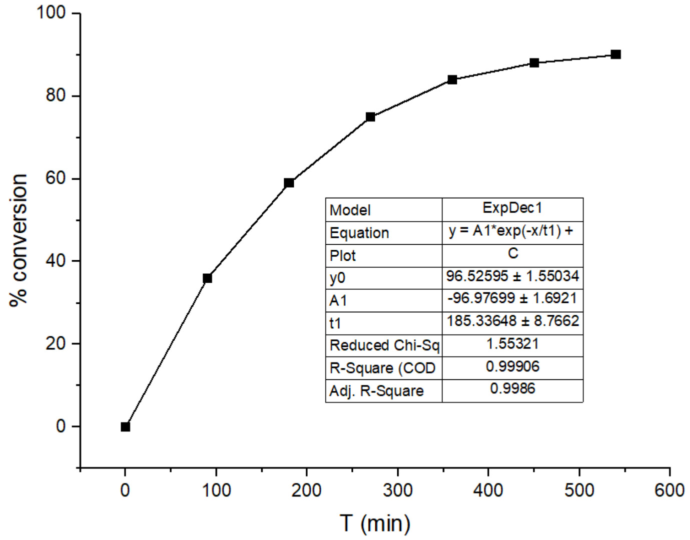

Figure S80: % deprotection of **1b** under 730 nm irradiation.

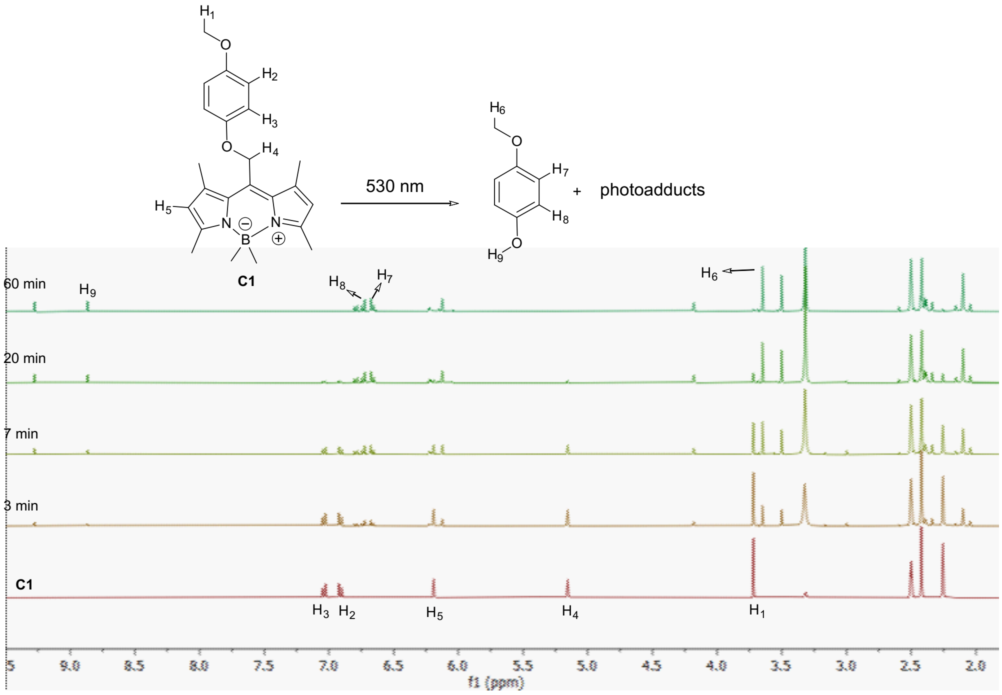

Figure 81: Photoirradiation of **C1** at 530 nm.

VIII. Stimulus-responsive ion transport activation

Activation of **1a** and **1b** with light in HPTS-based vesicles:

Preparation of POPC-LUVs⊃HPTS vesicles: These vesicles were prepared using the procedure as described above.

Experiment: In a clean and dry fluorescence cuvette, LUVs containing HPTS (40 μL, final lipid concentration 31.3 μM), and either **1a** (1.3 mol%), **1b** (0.67 mol%), or **C1** (1.5 mol%) were taken. A pulse of NaOH (30 μL, 0.5 M) was added at 20 s to generate the pH gradient across the membrane to initiate the ion transport, and finally, detergent (40 μL of Triton X-100 in 7:1 (v/v) H_2O-DMSO) at 300 secs to calibrate the assay. Negligible transport activity was observed for **1a**, **1b,** or **C1**. Samples containing HPTS-bound vesicles (40 μL) with **1a**, **1b**, or **C1** were photoirradiated with LEDs at either 530 nm (425 mW), 625 nm (920 mW), or 730 nm (1130 mW) for different time intervals, and ion transport was monitored after each photoirradiation process. A significant enhancement in the ion transport activity was observed upon photoirradiation for **1a** and **1b**, indicative of the formation of active transporter **1**. On the other hand, no change in the transport activity was observed for **C1** indicating no role of photoadducts in an overall transport process.

The time-dependent data were normalized to percent change in fluorescence intensity using Equation S1.

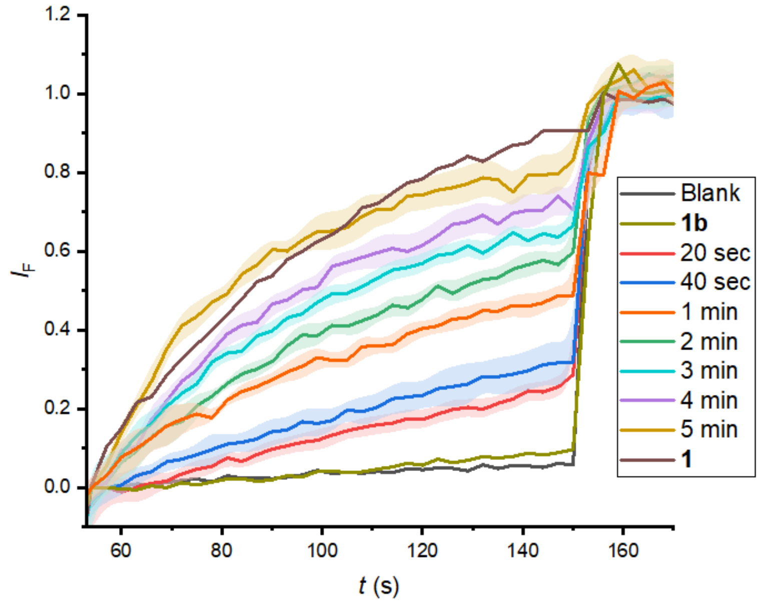

Figure 82: Transport activity of **1b** (1.3 mol%) upon photoirradiation at 625 nm.

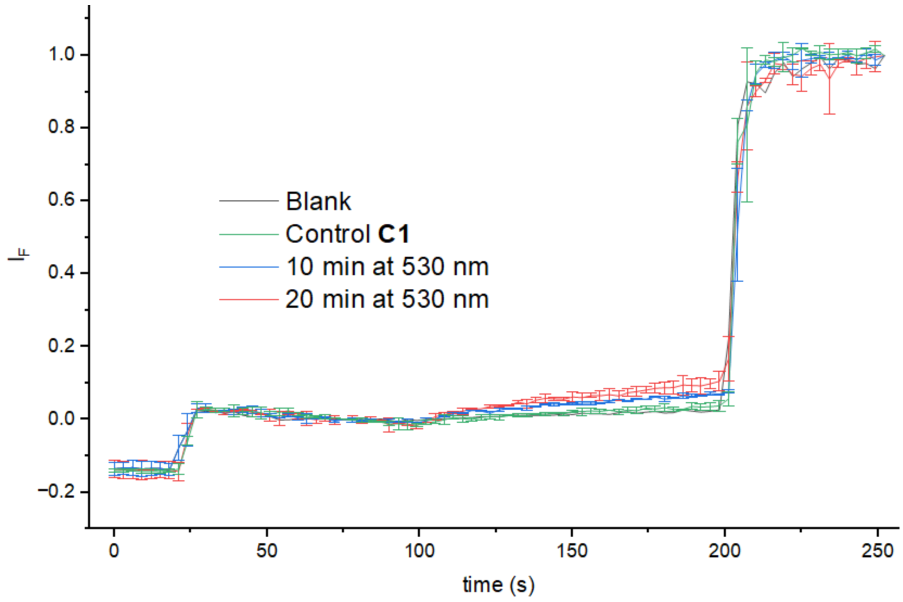

Figure 83: Transport activity of **C1** (1.5 mol%) upon photoirradiation at 530 nm.

IX. Biological studies

alamarBlue® cell viability assay. *MDA-MB-231* cells (ATCC, HTB-26) were seeded in 96-well plates and allowed to adhere and grow for approximately 24 hours in maintenance media (100 µL) at 37 °C in a 5% CO_2 incubator. The cells were then treated with varying concentrations of compounds in media (90 µL) for 1 h (final DMSO concentration <0.3%). Cells were then exposed to 730 nm LED (Thorlabs 730 nm mounted LED, M730L6, 1.1 W) for 5 or 30 minutes. alamarBlue® reagent (Thermo Fisher Scientific, Cat #DAL1025) (10 µL) was added directly to the cell culture media, and the cells were incubated in the dark for an additional 4 h at 37°C and 5% CO_2. The fluorescence (λ_{ex} = 560 nm, λ_{em} = 590 nm) was then measured using a FLUOstar Omega plate reader.

MQAE assay. Intracellular chloride concentration was measured using the chloride-sensitive fluorescent dye MQAE (N-(ethoxycarbonylmethyl)-6-methoxyquinolinium bromide, Biotium, Cat #52011). *MDA-MB-231* cells (ATCC, HTB-26) were seeded in 96-well plates and grown to approximately 80% confluence at 37°C in a 5% CO_2 incubator. For the experiments, a 5 mM working solution was freshly prepared in Hank's Balanced Salt Solution (HBSS) (Thermo Fisher Scientific, Cat #88284). After washing with DPBS, cells were incubated with 5 mM MQAE at 37 °C for 30 minutes in the dark. Following incubation, cells were washed three times with DPBS to remove extracellular dye. Cells were then incubated with various concentrations of compounds **1** or **1b** (final DMSO concentration <0.3%), with or without 30 min irradiation, and then fluorescence was measured over 2 hours. Fluorescence was measured using a FLUOstar Omega plate reader (λ_{ex} 350 nm, λ_{ex} 460 nm) at different time points (0, 15, 30, 45, 60, 90, 105 and 120 min). Fluorescence intensities were background-subtracted and normalised to the mean fluorescence of untreated control wells.

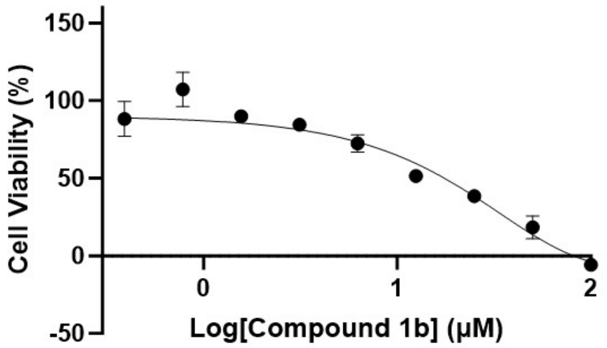

Fig. S84: Cell viability of compound **1b** in the absence of irradiation in *MDA-MB-231* cells assessed using the alamarBlue® assay following 4 h treatment. Data represent mean ± SEM (n = 4).

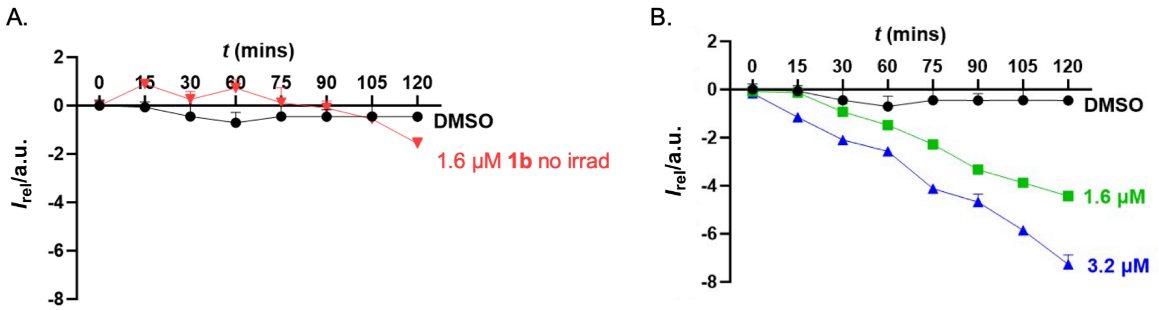

Fig. S85. (A) Chloride transport activity of pro-transporter **1b** (1.6 µM) measured using the MQAE fluorescence assay in *MDA-MB-231* cells following 4 h incubation. (B) Chloride transport activity of **1b** following irradiation at 730 nm for 30 mins, measured using the MQAE fluorescence assay in *MDA-MB-231* cells following 4 h incubation. Cells were treated with 1.6 µM (green), 3.2 µM (blue), or DMSO (black). Decreasing fluorescence corresponds to chloride influx. Data represent mean ± SEM (n = 4).

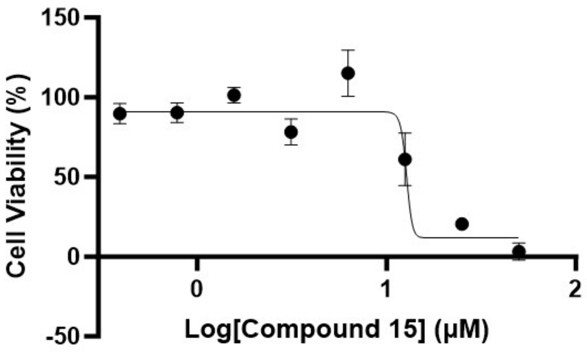

Fig. S86. Cell viability of compound **15** in *MDA-MB-231* cells assessed using the alamarBlue® assay following 4 h treatment. Data represent mean ± SEM (n = 4).

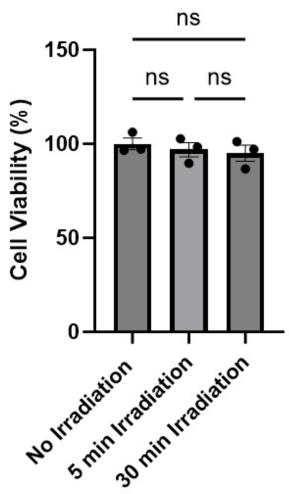

Fig. S87: Cell viability of *MDA-MB-231* cells following NIR irradiation (730 nm) in the absence of any compound. Cells were exposed to no light, 5 min, or 30 min of irradiation and assessed after 4 h using the AlamarBlue assay. No significant differences (ns) in viability were observed across conditions. Data represent mean ± SEM (n = 4).

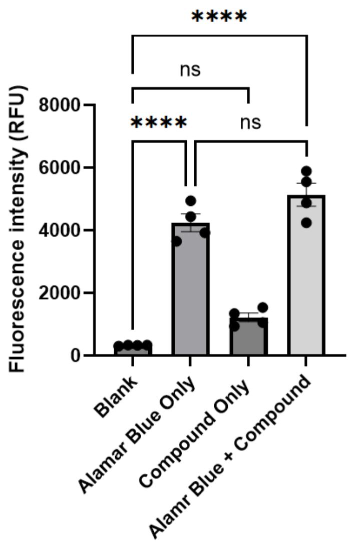

Figure 88. Assessment of spectral interference between compound 1 and Alamar Blue: Fluorescence intensity in the Alamar Blue detection channel was measured under four conditions: blank (medium only), Alamar Blue only (medium + Alamar Blue), compound only (medium + compound) and Alamar Blue + Compound. Bars represent mean fluorescence values ± SEM (n=4). P-value calculated using one-way ANOVA analysis: **** ≤ 0.0001, ns > 0.05.

X. References

(1) Da Lama, A.; Pérez Sestelo, J.; Sarandeses, L. A.; Martínez, M. M. Microwave-assisted direct synthesis of BODIPY dyes and derivatives. *Org. Biomol. Chem.* **2022**, *20* (46), 9132–9137.
(2) Shrestha, P.; Mukhopadhyay, A.; Dissanayake, K. C.; Winter, A. H. Efficiency of Functional Group Caging with Second-Generation Green- and Red-Light-Labile BODIPY Photoremovable Protecting Groups. *J. Org. Chem.* **2022**, *87* (21), 14334–14341.
(3) Gartland, S. A.; Johnson, T. G.; Walkley, E.; Langton, M. J. Inter-Vesicle Signal Transduction Using a Photo-Responsive Zinc Ionophore. *Angew. Chem. Int. Ed.* **2023**, *62* (38), e202309080.
(4) http://app.supramolecular.org/bindfit/
